# Restoration of Saccadic Eye Movements and Visually Guided Behavior in Ambient White Light with Photoswitchable Small Molecules

**DOI:** 10.1021/jacs.5c18611

**Published:** 2026-07-15

**Authors:** Rosalba Sortino, Aleix González-Díez, Santiago Milla-Navarro, Joaquín Martínez-Tambella, Víctor Paleo-García, Eric Calatayud, Paula de Saralegui, Ekin Opar, Àlvar Claparols, Josecarlo A. Quintanilla, Xavier Martínez-Soler, Fabio Riefolo, Carlo Matera, Jordi Hernando, Alexandre M. J. Gomila, Gerard Pérez-Batlle, Carles Pereira, Núria Camarero, Carme Serra, Xavier Gómez-Santacana, Amadeu Llebaria, Xavier Rovira, Pedro de la Villa, Pau Gorostiza

**Affiliations:** † Institute for Bioengineering of Catalonia (IBEC), Barcelona Institute for Science and Technology, Carrer de Baldiri Reixac, 10, 12, 08028 Barcelona, Spain; ‡ University of Barcelona Doctorate Program, 08028 Barcelona, Spain; § CIBER-BBN, Avenida Monforte de Lemos, 3-5, Pabellón 11, Planta 0, 28029 Madrid, Spain; ∥ Medicinal Chemistry and Synthesis (MCS) Laboratory, Institute for Advanced Chemistry of Catalonia (IQAC−CSIC), Carrer de Jordi Girona, 20, 08034 Barcelona, Spain; ⊥ University of Alcalá de Henares (UAH), Pl. de San Diego, s/n, Alcalá de Henares, Madrid 28801, Spain; # Institute Ramón y Cajal of Health Research (IRYCIS), Ctra. Colmenar Viejo, Fuencarral-El Pardo, Madrid 28034, Spain; ¶ Autonomous University of Barcelona (UAB), Plaça Cívica, Bellaterra, Barcelona 08193, Spain; ∇ Fundació Eduard Soler, Avinguda Eduard Soler, 1, Ripoll, Girona 17500, Spain; ○ Synthesis of High Added Value Molecules (SIMChem), Institut de Química Avançada de Catalunya (IQAC−CSIC), Carrer de Jordi Girona, 20, Barcelona 08034, Spain; ⧫ Catalan Institution for Research and Advanced Studies (ICREA), Barcelona 08010, Spain

## Abstract

Blinding diseases due to the degeneration of photoreceptors (PhRs), such as geographic atrophy (GA) secondary to dry age-related macular degeneration and retinitis pigmentosa (RP), leave the rest of the retinal circuitry largely intact, albeit unable to respond to light. Gene therapy has been able to revert PhR degeneration, but it can be applied only to a rare mutation affecting a small subset of RP patients. Alternatively, implanted electronic retinal prostheses aim at a larger population by electrically stimulating surviving neurons. However, the treatment is invasive and costly and provides limited resolution. Photopharmacology can develop photoswitchable small molecules to restore vision impairment by conferring light sensitivity to ion channels that are widely expressed in the remaining inner retinal neurons, and a first-in-human clinical trial is ongoing. Here, we have developed novel photoswitchable small-molecule ligands of metabotropic glutamate 6 (mGlu_6_) receptors, which are located exclusively at the dendrites of ON bipolar cells (postsynaptic to PhRs) and can leverage a privileged position to mimic physiological signals in the remnant retinal circuit. These photoswitchable ligands (prosthe6) thus act as “molecular prostheses” that can restore the light input to the retina via upstream-targeted control of the circuit after PhR degeneration. Prosthe6 compounds are allosteric, drug-like, water-soluble, and display outstanding *in vitro* properties including full efficacy, nanomolar potency, fast deactivation in ambient white light, and fast reactivation in the dark. *In vivo* experiments show that they readily recover the saccadic eye movements of blinded zebrafish larvae and restore the innate light-avoidance behavior in the mouse models of blindness (GA and RP). These effects are mediated by mGlu_6_ receptors *in vivo*. In addition, at least two compounds (prosthe6-**12** and -**15**) can restore sight by topical administration and display promising safety properties to become potential drug candidates for sight restoration in patients with degenerative blinding diseases.

## Graphical abstract

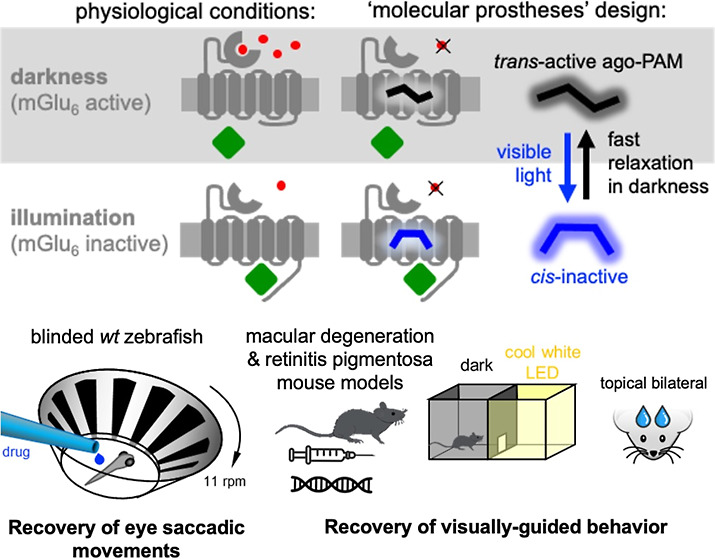

## Introduction

Most animals are diurnal and take advantage of daylight to interact with their environment by means of their visual system (eyes and visual brain regions). Sight allows us to sense our surroundings from a distance, and its importance is underlined by the large fraction of the brain that is involved in visual processing.^1^ It is by far the most valued sense in humans,^2^ and many languages have an over-represented visual vocabulary. Correspondingly, sight loss deeply impacts the ability to move, work, and socially interact. It causes delayed development and lower educational achievement in children as well as lower quality of life in adults.

Cataracts, refractive error, and geographic atrophy (GA) secondary to dry age-related macular degeneration (AMD) are the leading causes of visual impairment and blindness. The latter affects 200 million people worldwide,^3^ and, overall, they involve an estimated global yearly cost above US$ 400 billion.^4^ Addressing the unmet need of vision impairment from cataract and refractive error would cost much less, about US$ 25 billion.^5^ Yet, the cause of dry AMD, which accounts for 90% of all AMD cases, remains unknown, and current treatments consist of antioxidants, food supplements, and recently approved complement inhibitors that attempt to slow disease progression without significant improvements in best-corrected visual acuity.^6^ In rare inherited retinal dystrophies caused by biallelic *RPE65* mutations, typically diagnosed as Leber congenital amaurosis or retinitis pigmentosa (RP), gene therapy can restore visual function.^7^ In the case of RP, this treatment is applicable to only a small subset of patients (<1%)^8^ and costs nearly US$1 million per eye.

Since the process of photoreceptor (PhR) degeneration under these conditions spares all other neurons in the retina, visual functions can be restored in principle by controlling the electrical activity of the remnant neurons in a light-dependent manner using different approaches. Electronic retinal prostheses can be implanted to electrically stimulate the retina with spatiotemporal patterns acquired with a camera.^9,10^ However, they so far provide limited vision and usability, as they require training to interpret the new perceived image and to direct the gaze instead of moving the eye. They involve advanced surgical procedures that cost hundreds of thousands of US$, and their maintenance and discontinuation are ethically uncertain.^11^ Alternative approaches under development include photovoltaic materials^12^ and optogenetics,^10,13^ the latter being currently tested in several clinical trials^14–17^ fueled by promising results published in a patient in 2021.^18^

The electrical activity of retinal neurons can also be controlled with light using photoswitchable compounds targeting ion channels and receptors (photopharmacology).^19–24^ A clinical trial^25^ started in 2022 to restore sight with a photoswitchable blocker of hyperpolarization-activated cyclic nucleotide-gated channels (HCN channels)^26^ and preliminary results suggest good safety/tolerability and efficacy, which may open the way for a novel therapeutic avenue.^27^ HCN channels are highly expressed and play a key role in regulating neuronal excitability across the central nervous system, including retinal neurons, but their broad distribution hinders constraining drug action to specific locations in the circuit.

Targeting photoswitchable drugs to bipolar neurons, which are located at the top of the retinal circuit, has been postulated to offer unique advantages for restoring vision.^28,29^ By preserving the signal flow, this approach strictly preserves the encoding of the retinal circuitry and its extensive image-processing functions (orientation, motion, contrast, and color). Such “upstream targeting” is expected to offer a close-to-native retinal output.^24^ This powerful principle has been demonstrated with optogenetics^30,31^ but remains to be shown with photopharmacology.^32^

Here, we have targeted metabotropic glutamate 6 (mGlu_6_) receptors, which are exclusively expressed in ON bipolar cells (OBCs) and are localized postsynaptically to PhR cells,^33^ thereby leveraging a privileged position to drive physiological visual circuits^34^ (Figure 1). Pharmaceuticals are reversible and can be discontinued or upgraded when improved drugs are approved, in contrast to genetic manipulations. Pharmacotherapy is generally preferred by patients, clinicians, and public healthcare systems.^35^ In addition, the drug development, manufacturing, and clinical assessment processes are well established by the pharmaceutical industry and the regulatory agencies. To take advantage of all these benefits, we devised photoswitchable ligands of the mGlu_6_ receptor aiming at high clinical usability and odds of drug development success by means of the following requisites: (1) the mGlu_6_ photoswitches should be small drug-like molecules to facilitate development, efficacy and safety assays with conventional procedures, acceptance of patients and clinicians, and future production at affordable costs. (2) They should be water-soluble in <1% DMSO to facilitate biodistribution and formulation. (3) They should be active in the dark and deactivated with visible light (preferably white) to mimic physiological photoresponses of mGlu_6_ under sunlight. (4) They should relax in the dark to obtain rapid on–off photoresponses (temporal sensitivity) and thus high visual acuity. (5) The compounds should activate the target receptor with full efficacy to mimic physiological photoresponses of the mGlu_6_ receptor. (6) They should consist of a single chemical entity that produces both intrinsic activity and potentiation of mGlu_6_ (i.e., agonist and positive allosteric modulator, ago-PAM) to be effective in the absence of PhR-released glutamate and to facilitate regulatory processes. (7) High potencies in the nanomolar range are required to minimize the effective dose and off-target effects. (8) Compounds should display high photoswitching sensitivity (photosensitivity) under ambient lighting conditions, to avoid using light-enhancing devices as in optogenetic vision restoration.^18^ (9) They should restore photosensitivity and visual acuity independently of the cause of retinal degeneration (disease-agnostic). (10) They should have good bioavailability, preferably topical, which is associated with a higher success rate than systemic neurological drugs and to stronger patient adherence.

**Figure 1:**
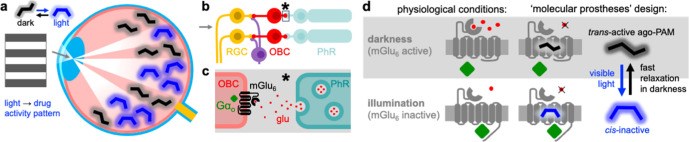
Concept of vision restoration based on upstream-targeted photoswitchable drugs. (a) Photoswitchable drugs isomerize under illumination, reversibly changing their activity (represented by black and blue isomers). When present in the eye, these compounds transduce external light stimuli projected to the posterior segment of the eye into patterns of pharmacological activity. Gray arrows mark the direction of light. (b) When PhRs (teal) are degenerated, drugs can thus photoswitch the activity of the surviving neural circuitry: RGCs, yellow; amacrine cells, purple; OBCs, red. PhR–OBC synapses, marked with a black box and asterisk (*), are located upstream in the circuit and constitute privileged cellular sites to mimic native activity with photoswitchable drugs. (c) mGlu_6_ receptor is an ideal molecular target for this purpose because it is exclusively localized in OBC dendrites and governs their excitability. Under physiological conditions in the dark, PhR-secreted glutamate (red dots) activates mGlu_6_ receptors, promoting GDP–GTP exchange on the associated heterotrimeric *G*_i/o_ protein. This triggers dissociation of the Gα_o_–GTP subunit (green rhombus) from the Gβγ complex and initiates downstream intracellular signaling. (d) Activated mGlu_6_ is shown again in dark conditions (top left). Under illumination (bottom left), PhRs reduce their glutamate secretion, and mGlu_6_ is inactive (G protein bound). To mimic physiological photoresponses when PhRs are degenerated, photoswitchable drugs should activate mGlu_6_ in the dark (top middle) and deactivate it under illumination (bottom middle), both in the absence of the agonist glutamate. Thus, photoswitchable molecular prostheses for upstream-targeted vision restoration should have agonistic intrinsic activity (allosteric agonist or ago-PAMs, agonist and positive allosteric modulators) of mGlu_6_ that are inactive under visible light and rapidly relax to become active in the dark (respectively blue and black on the right).

We have developed a set of photoswitchable compounds targeting mGlu_6_ that match this target product profile and functionally replace PhR activity (“prosthe6”). They allow restoration of saccadic eye movements and visually guided behavior in animal models of blindness, including RP and GA. They are amenable to further development as drugs and include promising candidates for future vision restoration therapies that will be generally applicable to retinal dystrophies, easy to use, and widely accessible to patients.

## Results

### Design and Photochemistry of Photoswitchable mGlu_6_ Ligands (Prosthe6) for Upstream-Targeted Vision Restoration

The activity of photoswitchable drugs can be reversibly controlled with light by chemical groups like azobenzene. The *trans*–*cis* photoisomerization of such a group changes the geometrical shape and electronic properties of the compound, altering the binding properties to the target protein and thus its pharmacological potency and/or efficacy.^36^ We previously developed small molecules with photoswitchable allosteric modulator activity in metabotropic glutamate receptors,^37^ including a PAM of mGlu_4_ and mGlu_6_ (named prosthe6-**1** in Figure 2).^38^ In contrast to other mGluRs with wider applications, the allosteric pharmacology of mGlu_6_ is largely unknown, and no structure of the full receptor has been reported to date.

**Figure 2:**
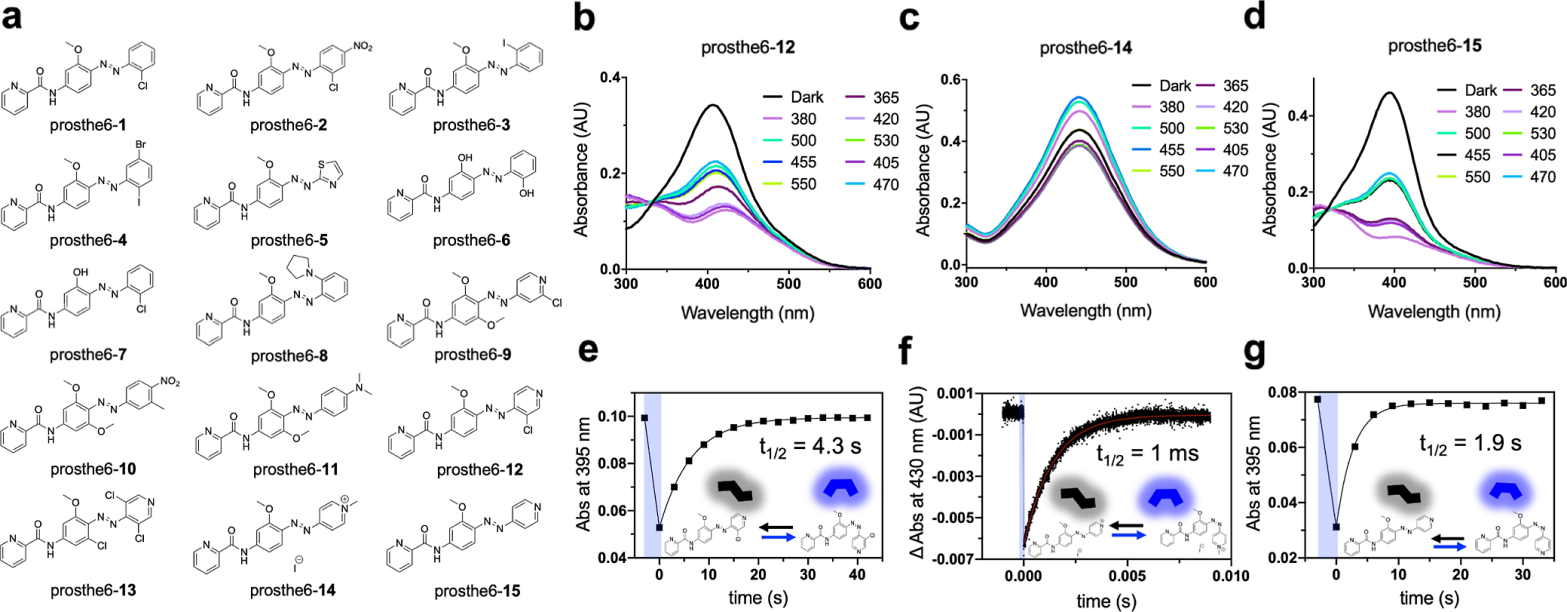
Design and characterization of photoswitchable mGlu_6_ ligands (prosthe6) with visible light absorption and fast relaxation in the dark. (a) Chemical structure of prosthe6-**1** ^37^ and 14 structural analogues (prosthe6-**2** to -**15**) designed to optimize the photopharmacological properties for vision restoration. (b–d) Example UV–visible light absorption spectra of prosthe6-**12**, -**14,** and -**15** in the dark and after illumination at different wavelengths indicate that they can be isomerized with visible light. Note that the DMSO solvent slows down thermal relaxation and allows measuring the absorption of the *cis* isomer after illumination. (e,g) Time course of the photoactivation and *cis*-state thermal relaxation of prosthe6-**12** and -**15** in DMSO/PBS 1:99 used in subsequent biological assays. The absorbance was measured at 395 and 381 nm, respectively, for prosthe6-**12** and -**15** after *in situ* irradiation of 10 μM solutions at 22 °C with a 395 nm LED at *t* = 0. The monoexponential fits (solid lines) yield half-lives of 4.3 and 1.9 s, respectively, for *cis* -prosthe6-**12** and -**15**. (f) Time course of the photoactivation and *cis*-state thermal relaxation of prosthe6-**14** in DMSO/PBS 1:99 used in subsequent biological assays. Transient absorbance was measured at 430 nm after a nanosecond laser pulse of 355 nm at *t* = 0. The monoexponential fit yields a *cis*-state thermal half-life of 1 ms.

Using fluorometry assays, we tested the activity of prosthe6-**1** in wild-type (wt) mouse and human mGlu_6_, as well as in the T171A mutant of mGlu_6_ (Figure S7), which is insensitive to orthosteric agonists such as glutamate and l-2-amino-4-phosphonobutyric acid (l-AP4; see Methods in the Supporting Information).^39,40^ The results of Figures S8–S11 show that prosthe6-**1** is a full ago-PAM with a potency around 800 nM in the dark or under 500 nm illumination (*trans*-isomer) and that it is deactivated by ultraviolet (UV) light of 380 nm (*cis*).^38^ These properties fulfill several conditions set above (1, 2, 5, 6, and partially 3 and 7), which prompted us to use it as a scaffold for further modifications.

The requisites of visible-light deactivation (3) and fast thermal relaxation in the dark (4) can be rationally addressed at once by introducing push–pull substituents that cause the destabilization of the highest occupied π-molecular orbital (electron-donating substituent) and the stabilization of the lowest unoccupied π*-molecular orbital (electron-withdrawing substituent).^41^ However, these may also alter the pharmacological activity if they affect the substitution pattern interacting with the mGlu_6_ allosteric binding site. Alternatively, the potency of prosthe6-**1** could be optimized prior to introducing electron-withdrawing substitutions, but these may eventually affect the pharmacological properties.

To solve this dilemma, we designed 14 analogues (prosthe6-**2** to -**15**, Figure 2) that explored a variety of structural modifications aiming at improving the potency and efficacy and/or refining the photoisomerization properties; i.e, achieving red-shifted absorption and/or faster *cis* relaxation kinetics through the introduction of strong electron-withdrawing and -donating groups (prosthe6-**2**, -**6**, **-7**, -**8**, -**10**, -**11**)^42^ and replacing the azobenzenic core by an azoheteroaromatic photochrome (prosthe6-**5**, -**9**, -**12**, -**13**, -**14**, -**15**).^43^ All designed compounds are drug-like^44^ (Table S3), and their computationally predicted toxicity^45^ is better than that of the drug candidate foliglurax,^46^ providing optimal safety scores for prosthe6-**11**, -**14**, and -**15** (Figures S15 and S16).

Nonpyridine derivatives prosthe6-**2** to -**5** and prosthe6-**10** to -**11** were obtained following standard diazotization and amination synthetic steps. In the case of prosthe6-**6** to -**7**, an extra stage was performed consisting of a demethylation reaction of the corresponding methoxy groups using BBr_3_ (Supporting Information and Figure S1). The same methodology was used to obtain 4-aminopyridine prosthe6-**9**, but no attempt to perform the diazotization of other 4-aminopyridines (prosthe6-**12**, prosthe6-**13**, and prosthe6-**15**) using the same methodology afforded the desired result. For this set of compounds, we used an alternative methodology that involves the use of HNO_3_ and H_3_PO_4_ and a different order of addition of reagents (see Supporting Information and Figure S2). Compound prosthe6-**14** was obtained by the chemoselective *N*-methylation of the *p*-substituted pyridine ring in compound prosthe6-**15**. The synthesis of compound prosthe6-**8** was approached differently (Supporting Information and Figure S3). Pyrrolidine was introduced in the scaffold by means of nucleophilic aromatic substitution, which is promoted by the electron-withdrawing nature of the azo bond and the fluorine atom as the leaving group. The subsequent aromatic amine acylation of the corresponding intermediate was accomplished by using picolinoyl chloride.

The identity and purity of all prosthe6 compounds were characterized by nuclear magnetic resonance (NMR) spectroscopy and high-performance liquid chromatography–mass spectrometry (see Methods in Supporting Information). UV–vis absorption spectroscopy showed that all prosthe6 compounds absorb in the visible range (Table S1 and Figure S4), thus fulfilling condition (3). Prosthe6 thermal relaxation half-lives ranged between milliseconds and hours (see Table S1 and Figure S4). Among others, the relaxation of prosthe6-**12**, -**14**, and -**15** is faster with half-lives of 4.3 s, 1 ms, and 1.9 s, respectively (Figure 2). The photostability of prosthe6-**12** was also assessed (Figure S5). Overall, these results indicate that prosthe6-**12**, -**14**, and -**15** also comply with condition (4).

### Prosthe6 are Nanomolar, Full ago-PAMs of Human mGlu_6_ and Can Be Deactivated with Visible Light

We next used fluorometry to characterize *in vitro* the pharmacological activity of the library in mGlu_6_ receptors (Figures 3 and S8–S14; see Methods in Supporting Information). We set up a protocol to screen the 14 photoswitchable allosteric ligands using a microplate reader. This method enables us to determine changes in intracellular calcium concentrations upon receptor activation. To validate the assay, both positive and negative controls were conducted (Figure S6). Initially, efficacy was tested across three concentrations (0.1, 1, and 10 μM) for the entire compound library in human embryonic kidney (HEK) cells co-expressing mouse wt mGlu_6_, a chimeric *G*_q/i_ protein,^47^ and the genetically encoded calcium indicator GCaMP6s (excitation 488 nm, emission 510 nm). *G*_q/i_ allows to couple mGlu_6_ activation to the phospholipase C pathway, thus inducing inositol 1,4,5-triphosphate (IP_3_) production and subsequent intracellular calcium release from the endoplasmic reticulum. This assay showed that all 14 compounds activate mGlu_6_ in their dark-adapted *trans*-state and about half display greater activity than prosthe6-**1** at all concentrations used (Figure S8a). In these experiments, cells were preincubated with DMEM GlutaMAX medium (which stabilizes glutamine concentration). In addition, the culture medium was aspirated immediately before the assay and replaced with a defined recording solution that does not contain glutamate, suggesting that the compounds may not require glutamate to activate mGlu_6_.

**Figure 3:**
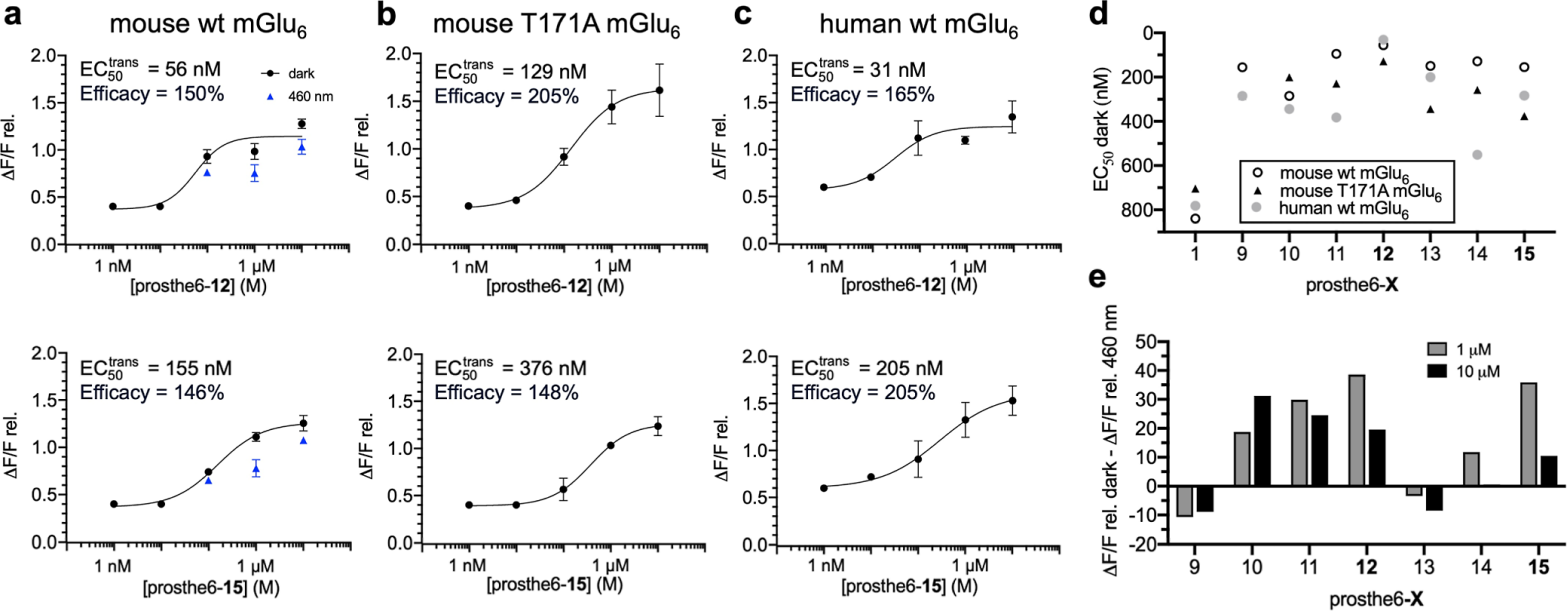
*In vitro* photopharmacological characterization of prosthe6 compounds. Dose–response curves of selected compounds prosthe6-**12** and -**15** (top and bottom rows, respectively) in (a) mouse wt mGlu_6_ including responses in the dark and after 2 min illumination at 460 nm (black circles and blue triangles, respectively), (b) mouse mutant T171A mGlu_6_ (which is insensitive to orthosteric agonists like glutamate and l-2-amino-4-phosphonobutyric acid; l-AP4), and (c) human wt mGlu_6_. Each curve is plotted as Δ*F*/*F* and relative to 10 μM prosthe6-**1** responses (see Supporting Information for details and Figures S8–S10 for results of other prosthe6). Responses are shown as the mean of at least three independent experiments (*n* = 3 replicates for each; error bars corresponding to the standard error of the mean, SEM) and were fitted to sigmoidal concentration–response curves with variable slope using GraphPad. (d) EC_50_ values of seven selected prosthe6 compounds tested in the three mGlu_6_ receptors. (e) Photoswitching of the seven selected prosthe6 compounds in mouse wt mGlu_6_ (difference in photoresponse amplitude between dark and illuminated compounds, Δ*F*/*F*_dark_ – Δ*F*/*F*_illum_) was evaluated at 1 and 10 μM concentrations (gray and black bars, respectively) and represented as %. Data are relative to 10 μM prosthe6-**1** responses. Positive values indicate higher mGlu_6_ activation in the dark than that after illumination, as required by condition (3) to mimic the physiological responses of ON bipolar cells (OBCs) to light (Figure 1d).

To confirm such ago-PAM activity, compounds were tested in glutamate-insensitive T171A mGlu_6_ receptors (Figure S7).^40,48^ The results of Figure S8b demonstrate that all 14 compounds can activate the mutant receptor, confirming their intrinsic agonistic activity and satisfying condition (6). The seven best-performing compounds in the set were selected for efficacy tests in HEK cells overexpressing human wt mGlu_6_ receptor and displayed positive results (Figure S8c). Notably, prosthe6-**12** and -**15** exhibited efficacies nearly twice as high as prosthe6-**1** in these experiments.

Dedicated concentration response curves (CRCs) were obtained to quantitatively evaluate the agonistic activity of these two prominent compounds in HEK cells overexpressing the mouse wt mGlu_6_ receptor (Figure 3a), T171A mGlu_6_ (Figure 3b), or the human wt mGlu_6_ receptor (Figure 3c). Their potency and efficacy were characterized, respectively, by calculating the compound concentration required to produce 50% of receptor activation (EC_50_) and the maximum response relative to that of prosthe6-**1**. Both compounds demonstrate high potency under all experimental conditions, as shown by EC_50_ values in the nanomolar range. The EC_50_ values for all seven compounds are summarized in Figure 3d along with the CRC depicted in Figures 3a–c and S9–S11. Prosthe6-**12** stands out as the most potent ligand in human mGlu_6_ receptors (31 nM), exhibiting a 25-fold increase compared to prosthe6-**1** (Figures 3c and S11). Both prosthe6-**12** and -**15** fully activate the receptor and show 1.5- to 2-fold higher responses than prosthe6-**1**. Figure S12 shows the correlation between EC_50_ values in mouse wt versus mutant T171A mGlu_6_ receptors, which is lower between EC_50_ values in mouse wt versus human wt mGlu_6_ receptors and between the efficacy values of these receptors.

We proceeded to evaluate the ability of the seven compounds to reversibly photoswitch mGlu_6_ receptor activity. We tested in mouse wt mGlu_6_ both the *trans* and *cis* forms of each compound (dark-relaxed and pre-irradiated for 2 min with blue light, respectively) at two concentrations (1 and 10 μM). Differences between the two photoisomers were detectable despite the fast *cis*-to-*trans* relaxation in the dark (Figures 3e and S13). Note that these end-point values in the plate reader assay underestimate real-time changes upon photoswitching due to the fast back-isomerization and the transient nature of calcium responses. The results are represented as the difference in response amplitude between dark-relaxed and blue-irradiated compounds (corresponding to *trans* and *cis* forms) and show that two compounds (prosthe6-**9** and -**13**) are more active in *cis*, whereas five out of seven (prosthe6-**10**, -**11**, -**12**, -**14,** and -**15**) are more active in *trans*. The latter thus meet prerequisite (3) set earlier. Notably, prosthe6-**12** and -**15** show the largest differences in the group (Figures 3e and S13). The CRCs of their *cis* isomers are depicted with blue triangles for mouse wt mGlu_6_ (Figures 3a and S9) and confirm a rightward shift of the plot upon illumination. All data presented in Figures 3, S8–S11,S13 are expressed relative to prosthe6-**1** (10 μM). Two representative examples of Δ*F*/*F* data sets (not relative to prosthe6-**1**) are shown in Figure S14. Overall, the *in vitro* characterization indicates that the two best-performing compounds, prosthe6-**12** and -**15,** entirely fulfill the photopharmacological criteria (3, 5–7 previously outlined) necessary for vision restoration purposes.

### Prosthe6 Compounds Enable the Restoration of Eye Saccadic Movements of Blinded Zebrafish Larvae by Light-Dependent Activation of mGlu_6_ Receptors

The promising efficacy, potency, and functionality of prosthe6 compounds upon visible-light irradiation *in vitro* prompted us to assess their effectiveness to restore visual acuity *in vivo* in a blindness model. Zebrafish larvae (*Danio rerio*) are suitable to study human visual disorders due to their cone-dominant vision and conserved physiology, visual neuronal circuit, and signal processing.^49^ Moreover, their retina is rapidly developed and shows visually guided behavioral responses from the fifth day post-fertilization (dpf).^50^ In addition, zebrafish larvae are susceptible to transient acute blindness, which can be induced by brief exposure to high-intensity light (100 W mercury lamp, see Supporting Information) that causes partial PhR death but spares the rest of the neural circuit.^51^

Zebrafish larvae display optokinetic reflex (OKR) response as humans (reflex saccadic movement to track moving lines), which provides a quantitative measurement of their visual acuity, i.e., their ability to detect edges in the field of view. This aptitude is complementary to photosensitivity, i.e., the ability to detect incoming light. We measured the saccadic movements performed by non-blinded and blinded larvae in an OKR assay. We employed a custom-built OKR device consisting of a rotating conic carousel with interspersed mirrors and windows, placed below a microscope coupled to a “cool white” light-emitting diode (LED) ring. This common light source offers wide emission including 460 and 550 nm bands from the blue dye and yellow phosphor, respectively. When the carousel rotates at an adjustable speed (6–60 rpm), white light reflected on the mirrors generates a moving pattern of highly contrasted light/dark stripes. Larvae are immobilized in the center of the carousel, and their eye movements are tracked with a camera (Figure 4a,b and Supporting Information Movies M1 and M2). They spontaneously track the rotating lines with slow eye movements and suddenly turn their eyes to the opposite side of their visual field (saccade), which produces a characteristic sawtooth pattern in intact animals (Figure 4c, black trace). We evaluated the optimal rotational speed under our experimental conditions among the ranges reported in previous reports, beginning at 6 rpm, which was the minimum rotational speed available in our device.^52^ All experiments were conducted at 11 rpm, which produced the maximal frequency of saccades in non-blinded larvae (Figure S17). Blinded larvae (Figure 4c, red trace) do not respond to rotating lines. Strikingly, they almost totally recover the wt reflex right after adding a 10 μL droplet of a solution of the tested compound (1 μM prosthe6-**12** or 50 μM prosthe6-**15**) directly on top of the larva. This indicates not only high efficacy (respectively reaching 75% and 40% of the OKR response in non-blinded larvae, Figure 4d,e) but also remarkable bioavailability and simplicity of use. We characterized the seven selected prosthe6 compounds using this assay with white light, and all of them significantly improved the OKR response (saccades/min) of blinded larvae (Figures 4e and S18). Prosthe6-**12** stood out among them, showing the highest recovery of edge detection ability at bath concentrations as low as 100 nM, in agreement with the high efficacy and potency demonstrated *in vitro* (Figures 3 and S19).

**Figure 4:**
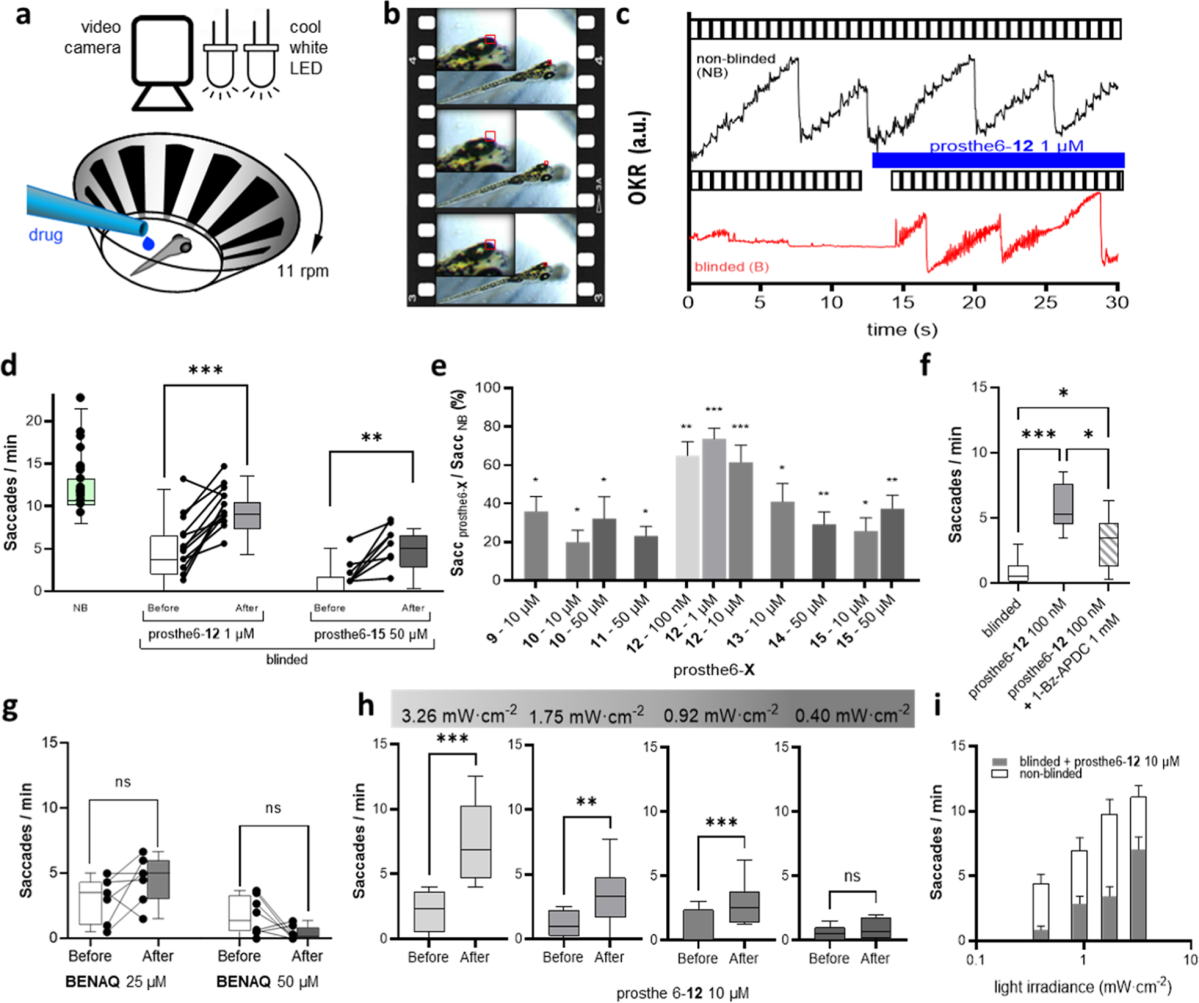
*In vivo* efficacy of prosthe6 compounds in restoring eye saccadic movements in a zebrafish larva blindness model. (a) Scheme of the setup to evaluate the OKR in zebrafish larvae. Larvae were immobilized (6% methylcellulose) in the center of a 45° truncated conic carousel with alternating mirrors and windows that rotated at a rotational speed of 11 rpm, as shown in the figure. A cool white LED and a video-camera were placed above the zebrafish to record 1 min videos. White light reflected on the mirrors projects a rotating pattern of vertical bright and dark stripes at the center of the carousel to photoswitch the compounds. (b) Three example frames show the eye rotating to focus its visual field on a point of the stimulus. As the carousel goes around, the larvae follow the point initially with a smooth movement, and when it exits the visual field, larvae recover their eye’s initial orientation with a characteristic fast movement (saccade).^53^ Mean grayscale intensity is measured in a region of interest at the edge of one eye (red box) to quantify eye movements as OKR signal (arbitrary units). (c) Representative example plots of eye movement during OKR experiments with intact (non-blinded (NB), top black trace) and blinded larvae (B, bottom red trace). During carousel rotation (striped bands), NB animals display a slow change in OKR signal, corresponding to the smooth eye movement during stimuli pursuit, followed by an abrupt change to recover the initial value after the saccade. B larvae change from irregular or no eye movement to control-like responses upon prosthe6–12 application in response to the rotating pattern. See also Supporting Information Movies M1 and M2. (d) Saccades per minute performed by NB larvae and B larvae before and after addition of either 1 μM prosthe6-**12** or 50 μM prosthe6-**15**. Connecting lines correspond to individual animals. Light irradiance was 3.26 mW·cm^–2^. Results are presented as mean ± SEM and individual responses. (**) *p* < 0.01, (***) *p* < 0.001 paired *t* test. (e) Performance of different prosthe6-**X** and concentrations in restoring saccadic eye movements of B larvae, represented here as saccades per minute after drug administration, normalized to the average saccades per minute of NB larvae (100%). Light irradiance was 3.26 mW·cm^–2^. Results are presented as mean ± SEM (*) *p* < 0.05; (**) *p* < 0.01; (***) *p* < 0.001 paired *t* test before and after drug administration (shown in panel d for 1 μM prosthe6-**12** and 50 μM prosthe6-**15** and in Figure S18 for the rest of molecules and concentrations). (f) Saccades per minute of B larvae before and after addition of 100 nM prosthe6-**12,** followed by the subsequent addition of selective mGlu_6_ agonist (2R, 4R)-4-amino-1-benzylpyrrolidine-2,4-dicarboxylic acid (1-Bz-APDC) (1 mM), indicating that the effect is mediated by mGlu_6_ receptors (see Figure S20 for individual larva responses). Light irradiance was 3.26 mW·cm^–2^. Results presented as mean ± SEM (*) *p* < 0.05, (***) *p* < 0.001, one-way ANOVA with Dunnett’s multiple comparisons test. (g) Saccades per minute of B larvae before and after addition of BENAQ 25 μM or 50 μM do not produce significant recovery of eye saccadic movements. Light irradiance was 3.26 mW·cm^–2^. ns = not significant, paired *t* test. (h) Effect of light irradiance on the recovery of eye saccadic movements of B larvae treated with prosthe6–12 (frequency of saccades before and after addition of 10 μM prosthe6-**12**; see Figure S21 for individual larva responses). Results presented as mean ± SEM (**) *p* < 0.01; (***) *p* < 0.001; (ns) = not significant, paired *t* test. (i) Effect of light irradiance on the recovery of saccadic eye movements with 10 μM prosthe6-**12** compared to the performance of NB larvae under the same illumination conditions. Results are presented as mean ± SEM.

To test whether the restoration of saccadic eye movements is due to photoswitchable regulation of mGlu_6_ by prosthe6, we performed an OKR assay, first applying prosthe6-**12** to blinded larvae and then adding the mGlu_6_ selective agonist (2*R*,4*R*)-4-amino-1-benzylpyrrolidine-2,4-dicarboxylic acid (1-Bz-APDC).^39,54^ Saccadic responses were significantly reduced in the presence of the nonphotoswitchable agonist, proving that mGlu_6_ mediates the prosthe6 mechanism of action (Figures 4f and S20 for paired behavior of individual larvae).

To benchmark the *in vivo* efficacy of prosthe6, we used BENAQ,^26^ the photoswitchable HCN blocker that is currently in clinical trials.^25,27^ We tested the OKR performance of BENAQ in blinded larvae around its reported working concentration. BENAQ did not significantly restore the restoration of eye saccadic movements of blinded larvae at 25 μM and 50 μM but caused apparent toxicity in the latter (Figure 4g). Thus, we used a reported zebrafish embryonic toxicity assay^55^ to test the safety of prosthe6-**12** and BENAQ. Embryos were incubated with the compounds from 6 to 120 h post-fertilization (hpf). At 120 hpf, 100 nM prosthe6-**12** caused a similar percentage of developmental abnormalities and deaths to those of the vehicle (DMSO 0.1%, Figure S22), whereas incubation in 10 μM BENAQ at 24 and 120 hpf produced the death of 96% and 100% of the embryos, respectively (Figure S22). These results can be attributed to the widespread expression of the HCN target and weak potency of BENAQ and to the systemic administration in our OKR assay. Thus, they do not contradict the preclinical and clinical safety studies in which BENAQ is locally administered through intravitreal injection without producing toxicity or being exposed to plasma in pharmacokinetic studies.^26,27^

We next assessed the light sensitivity of prosthe6-**12** by performing the OKR assay at different conditions of light irradiance, ranging from 0.40 mW·cm^–2^ to 3.26 mW·cm^–2^. Remarkably, prosthe6-**12** can still produce a significant increase in restoration of eye saccadic movements at 0.92 mW·cm^–2^, up to nearly 40% of the wt response at this irradiance condition (Figures 4h,i and S21).

Thus, prosthe6 compounds meet requirements (7) and (8) stated above. On the basis of these results, we retained for subsequent assays the compounds prosthe6-**12** (the most potent and efficacious in the library, considering *in vitro* and *in vivo* assays) and prosthe6-**15** (displaying the highest *in vitro* efficacy and best predicted toxicity profile). In particular, we carried out *in vitro* safety pharmacology profiles in a panel of 87 targets. The results demonstrate that prosthe6-**12** and -**15** interact with only one and three targets, respectively (Figures S23 and S24; see Supporting Information for details) and thus offer promising safety.

### Prosthe6 Compounds Restore Visually Guided Behavior in Mouse Models of GA and RP by Light-Dependent Activation of mGlu_6_ Receptors

We next investigated whether light-guided behavior could be restored in the established mouse models of GA and RP^56,59^ with the shortlisted compounds. For the GA model, we induced acute chemical damage of the PhRs with a sodium iodate intraperitoneal injection in *Opn4* ^–/–^ mice (P30–40) that do not express melanopsin. This approach yields completely blind animals after 28 days (P60–70; see Supporting Information Section S8) and is characterized by severe degeneration of the outer segment of PhRs, sparing the cell bodies, and by maintenance of the synaptic connections between bipolar neurons and the remaining PhR cell bodies.^56^ For the RP model, we used *pde6b* ^*rd10/rd10*^ × *Opn4* ^–/–^ mice (P95; see Supporting Information Section S8) that undergo progressive retinal degeneration leading to complete blindness in 60 days. This genetic model presents a degenerative process that mimics the death of PhRs in human RP. In addition, in advanced stages, there is a process of retinal remodeling in which the dendrites of the bipolar cells retract, due to the loss of synapses with the PhRs.^28,58^

Behavioral assays (light avoidance or negative phototaxis) were based on the innate preference of mice for dark spaces and used a box with two equally sized rooms connected by an opening, as shown in Figure 5a,c,e (see Supporting Information for details).^57^ One room was dark, and the other was lit either with blue LEDs (470 ± 10 nm emission, 0.095 mW·cm^–2^) or with cool white LEDs (including 460 and 550 nm bands, 0.018 mW·cm^–2^), both of which can induce *trans*-to-*cis* isomerization of prosthe6-**12** and -**15**. These illuminance values are comparable to those reported for this assay (see Figure 5 caption for irradiance values).^60^ Animals were released in the dark room and allowed to roam freely for 3 min. Every animal was tested three times to average the results. The time spent in each room was measured in each experiment and is shown in Figure 5 using dark and blue/white bars. Significant preference for the dark room indicates that the animal can perceive light.

**Figure 5:**
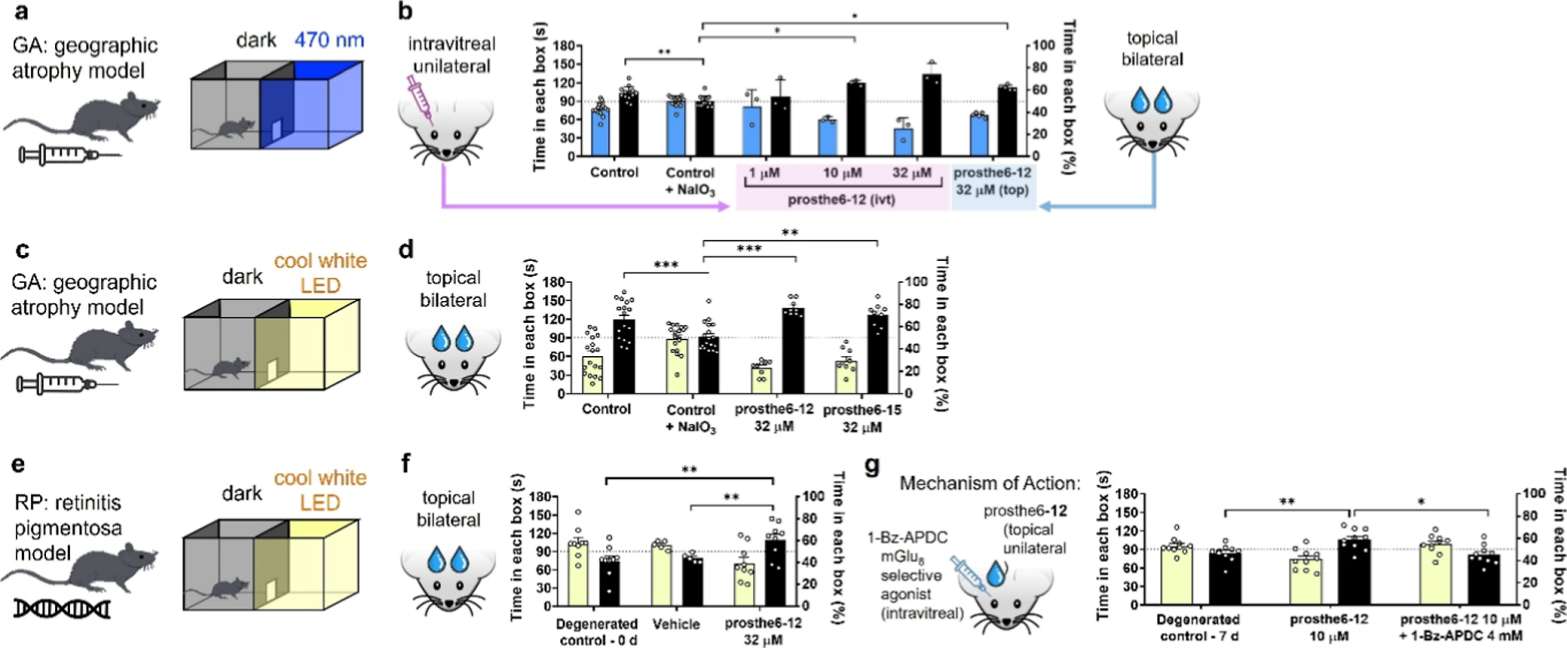
Prosthe6 compounds restore light-driven behavior in mouse models of GA and RP. (a) Effect of prosthe6-**12** in the mouse model of GA (*Opn*4^–/–^ mice not expressing melanopsin and whose PhRs were damaged by intraperitoneal injection of sodium iodate, >30 days after injection (P60–70, see Supporting Information Section S8)^56^ was evaluated using a light avoidance behavioral assay^57^ with rooms that are either dark (0.2 lx) or illuminated with 470 nm blue LEDs (385 lx, 0.095 mW·cm^–2^). The time spent in the dark and lit rooms of the box was quantified during 3 min, and results were averaged over three repetitions. (b) Healthy mice display an innate preference for the dark room in the box (predamage condition, *N* = 9), and GA mice cannot distinguish between dark and lit rooms after 28 days of blindness induction (postdamage, *N* = 9). Blind mice were injected intravitreally in the right eye 1 μL of prosthe6-**12** at concentrations 1, 10, or 30 μM and tested again after 2.5 h. They displayed significant preference for the dark room in all cases (*N* = 3, 3, 3, respectively). The experiment was repeated using topical application of 30 μM prosthe6-**12** in both eyes, and blind mice also recovered significant preference for the dark room (*N* = 9). (c) Using the same blindness model (GA), we tested the effect of cool white LED light in the behavioral assay (400 lx, 0.018 mW·cm^–2^, dimmer than daylight/office illumination). (d) Topical bilateral administration of 30 μM prosthe6-**12** produced a significant recovery of the dark room preference (*N* = 9). Similar results were obtained by topical bilateral administration of 30 μM prosthe6-**15** (*N* = **9**). (e) Genetic model of RP (*pde6b^rd10/rd10^* x *Opn4* ^–/–^ mice, >P90)^28,58,59^ undergoes progressive retinal degeneration leading to complete blindness in 60 days and was also tested with the light avoidance behavioral assay using cool white LEDs. (f) Topical bilateral administration of 30 μM prosthe6-**12** to degenerated blind animals (control 0d) produced a significant recovery of the dark room preference (*N* = 10), while the vehicle group did not show light sensitivity. (g) To test whether the restoration of light avoidance behavior is due to photoswitchable activation of mGlu_6_, we used the selective mGlu_6_ agonist (2*R*,4*R*)-4-amino-1-benzylpyrrolidine-2,4-dicarboxylic acid (1-Bz-APDC)^39,54^ administered only unilaterally, with an intravitreal injection. Topical unilateral administration of 10 μM prosthe6-**12** in the right eye still produced significant recovery of the dark room preference (*N* = 9). Intravitreal unilateral injection of 1 μL of 20 mM 1-Bz-APDC (final concentration in the eye: 4 mM) in the same eye significantly hindered the recovery of light avoidance by topical administration of 10 μM prosthe6-**12** (*N* = **10**). Statistical differences across b–d panels are determined by comparing the time recorded for each animal in the dark box, using mixed-effects ANOVA analysis (Tukey’s multiple comparisons). Statistical differences across f–g panels are determined by comparing the time recorded for each animal in the dark box, using repeated measures one-way ANOVA analysis, respectively (Tukey’s multiple comparisons) ((*) *p* < 0.05, (**) *p* < 0.01, and (***) *p* < 0.001). Data are presented as mean ± SEM of the average times recorded for the dark and light boxes, to facilitate comparison of the results. Although there is no equal distribution of sexes in all groups, no significant differences between sexes were found for those groups with a homogeneous distribution (Figure S24). None of the animals showed signs of pain, distress, or morphological alterations in the eye by either route or compound.

Assays were carried out at several time points: before and after PhR damage and after drug application. We recently showed that photoswitchable small molecules can be readily bioavailable using eye drops applied to the cornea.^61^ Thus, we tested intravitreal and topical routes with the selected compounds prosthe6-**12** and -**15**. For the first study, we used unilateral intravitreal injections of different concentrations (1, 10, and 30 μM in 1.6% DMSO) of prosthe6-**12**. Subsequent studies shown in Figure 5 used bilateral or unilateral topical application of 10 μL prosthe6-**12** or -**15** at 30 μM (1.6% DMSO) without washout in both animal models.

Prior to sodium iodate injection (GA model), animals display a preference for the dark room, but 28 days after retinal damage, they cannot distinguish between the rooms. This effect is similar to that observed in the retinal degeneration animal model (*pde6b* ^*rd10/rd10*^ × *Opn4* ^–/–^ up to 90 days old, RP) (“post-damage” condition, Figure 5b,d,f, respectively), while no changes were observed in the RP animals treated with the vehicle (1.6% DMSO in PBS). Preference for the dark room was significantly restored 2.5 h after administration of prosthe6-**12** or -**15**, either by intraocular injection or topical routes, with blue or white light, and in both animal models of blindness (see Figure 5 for statistical analysis and Supporting Information for technical details). None of the animals showed signs of pain, distress, or morphological alterations in the eye by either route or administered compound.

We complemented these results with pharmacokinetic studies after intravitreal and topical administration of prosthe6-**12** in White New Zealand rabbits (Supporting Information, Figure S26 and Table S6). The unformulated compound was detected in the retina up to 6 and 12 h post-topical and intravitreal administration, respectively, and it was not found in plasma by either route, indicating promising bioavailability and safety profiles.

To test whether the restoration of light avoidance behavior is due to photoswitchable activation of mGlu_6_, we repeated experiments with topical unilateral 32 μM prosthe6-**12** and white light in RP mice before and after unilateral intravitreal injection of 1 μL of 20 mM 1-Bz-APDC (final concentration in the eye: 4 mM; note that binocular injection of the mGlu_6_ agonist was not possible due to ethical concerns). The results of Figure 5g show that preference for the dark room can still be restored by unilateral topical administration of prosthe6-**12** and that this behavior is significantly reduced with 1-Bz-APDC, confirming our hypothesis in a mammalian animal model.

Overall, the results of Figures 5 and S25 demonstrate that prosthe6-**12** and -**15** meet requirements (9) and (10) and constitute suitable photopharmacological compounds for upstream-targeted vision restoration.

## Discussion

Twenty years ago, the restoration of visual function seemed to be a daunting task, all but impossible. Breakthroughs in retinal prostheses and gene therapy (clinically approved ten and seven years ago, respectively) and in stem cell transplantation and optogenetics have produced promising results and good reasons for optimism. However, each of these strategies has its own shortcomings.

Prosthetic electronic implants require invasive surgery, extensive training to direct the gaze and to interpret the retinal stimuli, and some of them have been discontinued due to the reduced visual resolution provided.^62^ Direct electronic stimulation of the visual cortex has been recently reported in primates^63^ and humans^64^ as an alternative to retinal prostheses. Stem cell-derived PhR grafting is also an invasive and irreversible intervention, and it is prone to neoplastic changes.^65^ Optogenetic approaches introduce microbial opsins (e.g., ChR2 and its variants) with viral vectors to turn light-responsive the neurons that are spared in degenerative retinal diseases (e.g., OBCs or retinal ganglion cells (RGCs)).^66,67^ Three clinical trials^16,17,68^ are underway to test them, and one of them has reported partial functional recovery in a blind patient.^17^ However, microbial opsin photosensitivity is lower than that of mammalian opsins and requires using goggles to deliver a high-intensity image to the retina,^14^ which can damage the surviving PhRs. Moreover, microbial opsins are exogenous proteins that can cause immunogenic adverse effects. Optogenetic strategies that use mammalian opsins with higher photosensitivity have also been developed.^69,70^ They can restore basic perception, but their slow kinetics does not support motion vision and provides low resolution.^71^ The development of melanopsin-mGlu_6_ chimeras (Opto-mGlu_6_) overcame this shortfall by riding the physiological Gα_o_-mediated mGlu_6_ pathway.^31,72^ Using modified adeno-associated virus capsids and OBC-specific promoters to overexpress Opto-mGlu_6_ in the retina of blind mice allowed to recover their visual acuity.^31,73^ However, all these protein engineering approaches require irreversible genetic manipulation, which has unknown long-term impact and raises moral, regulatory, safety, and tolerability issues that call to consider other methods also to restore visual functions.

Regarding photoswitchable compounds, covalently tethered ligands have been shown to enhance photosensitivity^20^ and visual acuity^74^ in an animal model of retinal degeneration. Yet, they require a second component for tethering the photoswitch (a target protein introduced by genetic manipulation) that makes this approach irreversible, like optogenetics, and poses the same safety and regulatory concerns. Alternatively, one-component photoswitches that covalently attach to endogenous protein targets can rapidly photosensitize degenerated retina *ex vivo* ^21^ and cochlea *in vivo*,^75^ but none of these tethered compounds are drug-like due to their large mass and big number of hydrogen-bond donors and acceptors.

In contrast, one-component photoswitches that freely diffuse in tissue are simple and offer the same advantages as those of small-molecule drugs. They are reversible, easy to administer, and potentially less immunogenic than two-component chemical optogenetic interventions.^23,29^ Once they prove to be clinically useful, they will be more affordable and widely applicable than other therapies. A first-in-human trial of a photoswitchable drug (BENAQ) is currently ongoing.^25–27^ This and other compounds have shown efficacy for vision restoration, but they target proteins that are widely expressed throughout the central nervous system (potassium and HCN channels)^19,22,32,76,77^ which might cause adverse effects. Nevertheless, the safety results reported recently for BENAQ are promising and will spark new developments.^78^

Despite these advances, the restoration of eye saccadic movements with photoswitchable drugs has never been reported. To achieve such an optimized performance, compounds should act at specific locations in the retinal circuit, ideally at bipolar cells and next to the PhR signal source (upstream targeted photopharmacology).^24,28,29^ This is where image processing is carried out, including the encoding of orientation, motion, and contrast. BENAQ and its analogues DENAQ, PhENAQ, and DAQ act downstream on RGCs, and DAA acts on amacrine cells.^26,76,77^ DAD is a photoswitchable analogue of lidocaine (a local anesthetic and sodium channel blocker) that effectively photosensitizes degenerated retinae with white light. It photoswitches the activity of K_V_ and hyperpolarization-activated cyclic nucleotide-gated (HCN) channels, but its main target in bipolar cells remains unknown.^32^ Although blind animals recover photosensitivity upon intravitreal injection of 5 mM DAD, they prefer the light side of the box, in contrast to their innate behavior and to the effect of prosthe6 shown in Figure 5. In addition, the working concentration of prosthe6-**12** and -**15** in mice is more than 100-fold lower, both intravitreal and topical. Besides, to the best of our knowledge, the activity of BENAQ in the retina has only been reported *ex vivo*.^26,76^

Thus, conferring light sensitivity to mGlu_6_ receptors in OBCs might produce more complex and native-like output signals, which maintain the inner retinal processing and preserves the RGC-receptive fields.^28,79^ This likely enables prosthe6 to restore higher quality vision compared to other small-molecule photoswitches and may also offer a more intuitive vision restoration experience than camera-based electronic prostheses. Prosthe6 photocontrols mGlu_6_ receptors that are exclusively expressed in OBCs and are postsynaptic to PhRs under physiological conditions. They can be envisaged as synthetic PhRs or molecular prostheses, allowing one to retain the functionality and physiological signal flow of the circuit when PhRs are degenerated. Moreover, these compounds bind to allosteric sites, which are less conserved across receptor subtype families and are more advantageous than orthosteric ligands.^80^

To achieve these results, we investigated the mGlu_6_ pharmacology, which was practically unexplored, and obtained high success rates *in vitro* (seven out of 14 full ago-PAMs with nanomolar potencies) and *in vivo* (restoration of eye saccadic movements and visually guided behavior), demonstrating that the target is druggable. Prosthe6 compounds offer a first glimpse at the structure–activity relationship of the mGlu_6_ allosteric binding site (Figures 3 and S9). The most prominent trend is that *trans* isomers display enhanced efficacy (up to 150%) and potency (down to 50 nM) if a nitrogen atom is introduced in the *para* position in the right ring, which also increases compound polarity. Other substitutions cause minor changes, except occupying all *ortho* positions, which reduces efficacy to 80%. The *cis* isomer is generally less active, as intended, but two behaviors can be distinguished: a potency photoswitching^81^ when nitrogen is in the ring (prosthe6-**9**, -**12**, -**14**, and -**15**) and an efficacy photoswitching^82^ for bulkier substituents like nitro and dimethylamine (prosthe6-**10** and -**11**, respectively).

We have also determined the mechanism of action of prosthe6-**12** using mGlu_6_ selective ligands in two animal models, which secures target engagement and eliminates uncertainty for future drug development. Demonstrating the activity of prosthe6 in human mGlu_6_ receptors is also important for derisking because some mGluR allosteric ligands display a disconnect between rat and human mGlu_6_ activities.^83^ Our results confirm a weak correlation between them, both in terms of potency and efficacy (Figure S12), but prosthe6-**12** has outstanding scores in human mGlu_6_ (respectively 30 nM and 200% of prosthe6-**1** efficacy), and other excellent candidates like prosthe6-**11** are available. Other key risk factors for prosthe6’s progress toward clinical trials are their metabolism and wide pharmacological profile, which are under study but out of the scope of this basic research report.

We have shown that at least seven out of 14 prosthe6 compounds restore eye saccadic movements in a zebrafish model of blindness. Remarkably, they act right after adding them to water, do not require eye injection, and are not toxic to the larvae up to 5 dpf. These results demonstrate that prosthe6 are good lead compounds and that their efficacy and safety can be further optimized following established medicinal chemistry and pharmacological procedures for drug development.

The bibliography showed no evidence that intravitreal or topical administration of low doses of DMSO (<2%) could cause behavioral alterations in animals, such as photophobia.^84^ Therefore, it was decided to perform controls with vehicle alone for the RP animal model (Figure 5f). Prosthe6-**12** and prosthe6-**15** compounds restore visually guided behavior by topical administration without formulation in the mouse models of GA and RP.^84^ In particular, prosthe6-**12** is exposed to the retina up to 6 h after administration by either route in rabbits, which are commonly used as predictors of human ocular pharmacokinetics.^85^ The preference for darkness elicited by prosthe6 in blind mice mirrors the innate behavior and is in contrast with the preference for light produced by DAD.^32^ Exposure to the retina in larger eyes (e.g., pig, dog, human) may be lower than that in mice and requires formulation. Clinically approved intraocular sustained-release implants can maintain therapeutic drug levels in the posterior segment for months to years, and formulation advances may enable topical approaches to sustain effective concentrations in the retina in the future.^86^ Yet, if topical administration is not possible, intraocular injection (as used with BENAQ)^25^ remains a feasible and acceptable option, given the lack of therapeutic alternatives.

According to the principle of upstream targeting,^28,29^ the privileged localization of mGlu_6_ receptors at bipolar cell dendrites supports the high efficacy of prosthe6 to restore eye saccadic movements and visually guided behaviors. The partial retraction of bipolar dendrites upon degeneration (retinal remodeling)^28^ is a potential weak spot of this strategy compared to broadly and highly expressed targets like K_V_ or HCN channels. However, the excellent recovery of vision observed in 90 day old RP model mice (Figure 5f,g), whose retinas are fully remodeled,^87^ suggests that the remnant expression and localization of mGlu_6_ receptors may suffice to provide the advantages of upstream targeting.

The absence of apparent adverse effects in fish and mice requires in-depth characterization but agrees with the extremely localized expression and specialized function of mGlu_6_ in animals, and with the general advantageous properties of allosteric modulators,^88^ which are observed in the pharmacological profiles of prosthe6-**12** and -**15** (Figures S23 and S24). Moreover, the lack of plasma exposure observed in rabbit pharmacokinetics by both routes suggests that the drug might be confined to the eye, offering promising safety (Table S6).

The irradiance values employed for the light avoidance behavioral assay in mice are 0.018 and 0.095 mW·cm^–2^ for the white light and blue light boxes, respectively, corresponding to a dark overcast day. Moreover, the threshold for the restoration of eye saccadic movements in zebrafish larvae is 0.9 mW·cm^–2^, within the range of common indoor irradiance, which is sufficient for a physiological retina to enable appropriate visual function in the photopic range.^89^ Results from both models confirm the high photoswitching sensitivity of prosthe6-**12** and -**15**, showing that these compounds can be effectively stimulated under physiological irradiance values. Thus, unlike optogenetic assays, prosthe6 compounds do not require high-irradiance devices that can lead to phototoxicity. When comparing with other reported thresholds for restoration of visual responses, in most cases, they were determined in *ex vivo* experiments with isolated retinae and are in the same range of our results in mice (although the actual retina irradiance through the pupil is likely lower).^19,26,32,77^ The irradiance values employed in our light avoidance experiments are similar to those used in optogenetics assays in animals (learned visually guided behavior with rhodopsin^70^ or optomotor reflex with melanopsin chimeras),^31,79^ which are 1000-fold lower than those used for DAD in the same assay (18 mW·cm^–2^)^32^ and 500-fold lower than that used for DENAQ in open-field experiments (9.9 mW·cm^–2^).^77^ Compared to the light avoidance assay with optogenetics (MW-opsin and ReaChR)^66,90^ and with tethered photoswitch-based strategies (SNAG-mGluR2),^74^ prosthe6 afford more than 5-, 80-, and 60-fold improvement, respectively. In addition, prosthe6 use 700-fold dimmer light than in an optogenetic clinical trial (NCT03326336).^18^ The greater light sensitivity of prosthe6 compared to other photoswitchable small molecules may be due to G protein amplification and/or to the high potencies of prosthe6 (i.e., photoswitching fewer molecules is enough to activate the target protein and causes weaker absorbance through the vitreous).

Besides, relaxation half-lives in the dark can be as short as 1 ms (Figure 2), which is 2 orders of magnitude faster than the recovery rate of rod PhRs.^91^ At equivalent potency and efficacy, fast relaxing prosthe6 are expected to deliver superior visual acuity (lower image latency). However, this comes at the expense of photosensitivity, due to the higher intensity required to reach the photostationary state.^92^ A trade-off can be set at relaxation half-lives around the PhR recovery rate (approximately 200 ms), which suggests that faster relaxing compounds than prosthe6-**12** and -**15** may afford even higher photosensitivity without compromising their efficacy *in vivo*.

Unlike (opto)­genetic manipulations and surgically implanted retinal electronic prostheses, pharmacotherapy is noninvasive, readily reversible, and can be upgraded when new drugs are approved. Medicines are preferred by patients, clinicians, and public healthcare systems; they can be developed and manufactured at lower costs than other approaches and assessed by conventional regulatory procedures and clinical assays.

From a fundamental perspective, prosthe6 constitute new tools for ophthalmology to study the physiopathology of mGlu_6_ receptors and retinal circuits *in vitro* and *in vivo* and contribute to the medicinal chemistry of allosteric modulators. They also achieve the prediction that upstream-targeted photopharmacology can deliver nearly native output signals, taking full advantage of the retinal circuit for high-quality vision restoration.^28,29^

Thus, this contribution constitutes a disruptive step toward a vision restoration therapy based on drugs. Prosthe6 compounds may help recover a high level of sight regardless of the cause of retinal degeneration (mutation agnostic) and by means that are noninvasive, widely available to patients, and affordable for public health systems.

## Conclusions

We have devised a strategy to restore sight with photoswitchable small-molecule ligands of mGlu_6_ receptors, which are located exclusively in OBCs (postsynaptic to PhRs) and exert an advantageous role in the retinal circuit. For that purpose, we explored the uncharted allosteric pharmacophore of mGlu_6_ and identified drug-like, water-soluble ligands affording ago-PAM full efficacy and potency of tens of nanomolar. Their activity is reduced under ambient visible light, and they rapidly revert to the mGlu_6_-active conformation in the dark. Thus, prosthe6 compounds mimic the effect of PhRs on OBCs under physiological conditions and can replace their function when PhRs are absent due to retinal degeneration. As a result, prosthe6 compounds restore eye saccadic movements in blinded zebrafish larvae and innate light avoidance behavior in two mouse models of retinal degeneration, thereby offering good prospects for disease agnostic therapies based on drugs. In addition, at least two compounds (prosthe6-**12** and -**15**) appear to be devoid of adverse effects and restore sight by topical administration, which is linked to a higher overall clinical success rate than systemic routes for neurological drugs, and to stronger patient adherence.

## Supplementary Material







## Notes

Detailed synthetic procedures and full chemical characterization of all prosthe6 compounds, along with their photochemical properties (absorption spectra, relaxation kinetics, and photostability) comprehensive *in vitro* pharmacological data on mGlu_6_ receptors along with control experiments; toxicity and drug-likeness predictions; *in vivo* methodologies and results in zebrafish larvae and mouse models of blindness; and safety profiling and pharmacokinetic studies supporting the efficacy and translational potential of the lead compounds (PDF)
Saccadic movements performed by non-blinded larvae (MP4)
Saccadic movements performed by blinded larvae (MP4)

## ††

A.G.D.: AkzoNobel company, Carrer de la Feixa Llarga, 14–20, Sants-Montjuïc, 08040 Barcelona

## ‡‡

F.R.: Teamit Institute, Carrer de Sant Antoni Maria Claret, 167, Horta-Guinardó, 08025 Barcelona.

## §§

C.M.: University of Milan, Via Luigi Mangiagalli, 25, 20133 Milan, Italy.

## ∥∥

A.M.J.G.: Forschungszentrum Jülich, Wilhelm-Johnen-Straße, 52428 Jülich, Germany.
