# Supplementary material for: Restoration of Saccadic Eye Movements and Visually Guided Behavior in Ambient White Light with Photoswitchable Small Molecules
Source: J Am Chem Soc. 2026 Jul 15;148(30):31701–15. doi: 10.1021/jacs.5c18611 (PMC13450387; doi:10.1021/jacs.5c18611)
Supplement: Supplementary file 1 [file ja5c18611_si_001.pdf]

SUPPORTING INFORMATION

Restoration of saccadic eye movements and visually guided behavior in ambient white light with photoswitchable small molecules

Rosalba Sortino, Aleix González-Díez, Santiago Milla-Navarro, Joaquín Martínez-Tambella, Víctor Paleo-García, Eric Calatayud, Paula de Saralegui, Ekin Opar, Àlvar Claparols, Josecarlo A. Quintanilla, Xavier Martínez-Soler, Fabio Riefolo, Carlo Matera, Jordi Hernando, Alexandre M. J. Gomila, Gerard Pérez-Batlle, Carles Pereira, Núria Camarero, Carme Serra, Xavier Gómez-Santacana, Amadeu Llebaria, Xavier Rovira, Pedro de la Villa, Pau Gorostiza

Table of Contents

1. Synthetic procedures......2

Materials and general methods......3

Synthesis of Intermediates......4

Synthesis of Final Compounds......12

Synthesis of (2R, 4R)-4-amino-1-benzylpyrrolidine-2,4-dicarboxylic acid (1-Bz-APDC)......19

2. Photochemical characterization of prosthe6 compounds (absorption, slow/fast relaxation and photostability)......19

Prosthe6 UV-Vis absorption spectra, aqueous solubility and relaxation time measurement......19

Prosthe6-12 and 15 relaxation time measurement in PBS: DMSO 99:1......19

Prosthe6-14 relaxation time measurement in PBS: DMSO 99:1......19

Assessment of prosthe6-12 photostability......26

3. In vitro photopharmacological characterization in overexpressed metabotropic glutamate 6 (mGlu$_6$) receptors......27

4. Toxicity and drug-likeness prediction of seven prosthe6 compounds.......33

Drug-likeness calculations......35

5. In vivo photopharmacological characterization in zebrafish larvae......36

Zebrafish housing......36

Blinding Procedure......36

Optokinetic reflex (OKR) assay......36

6. In vivo toxicity in zebrafish larvae......40

7. In vitro pharmacology of prosthe6-12 and 15......41

8. Light driven behavior in mouse models of macular degeneration (GA) and retinitis pigmentosa (RP)......46

Animals......46

Intravitreal Injection......47

Topical Application......47

Experimental Procedure 47
9. Pharmacokinetic profile of prosthe6-12 .... *49*
10. Supplementary References *51*

1. Synthetic procedures

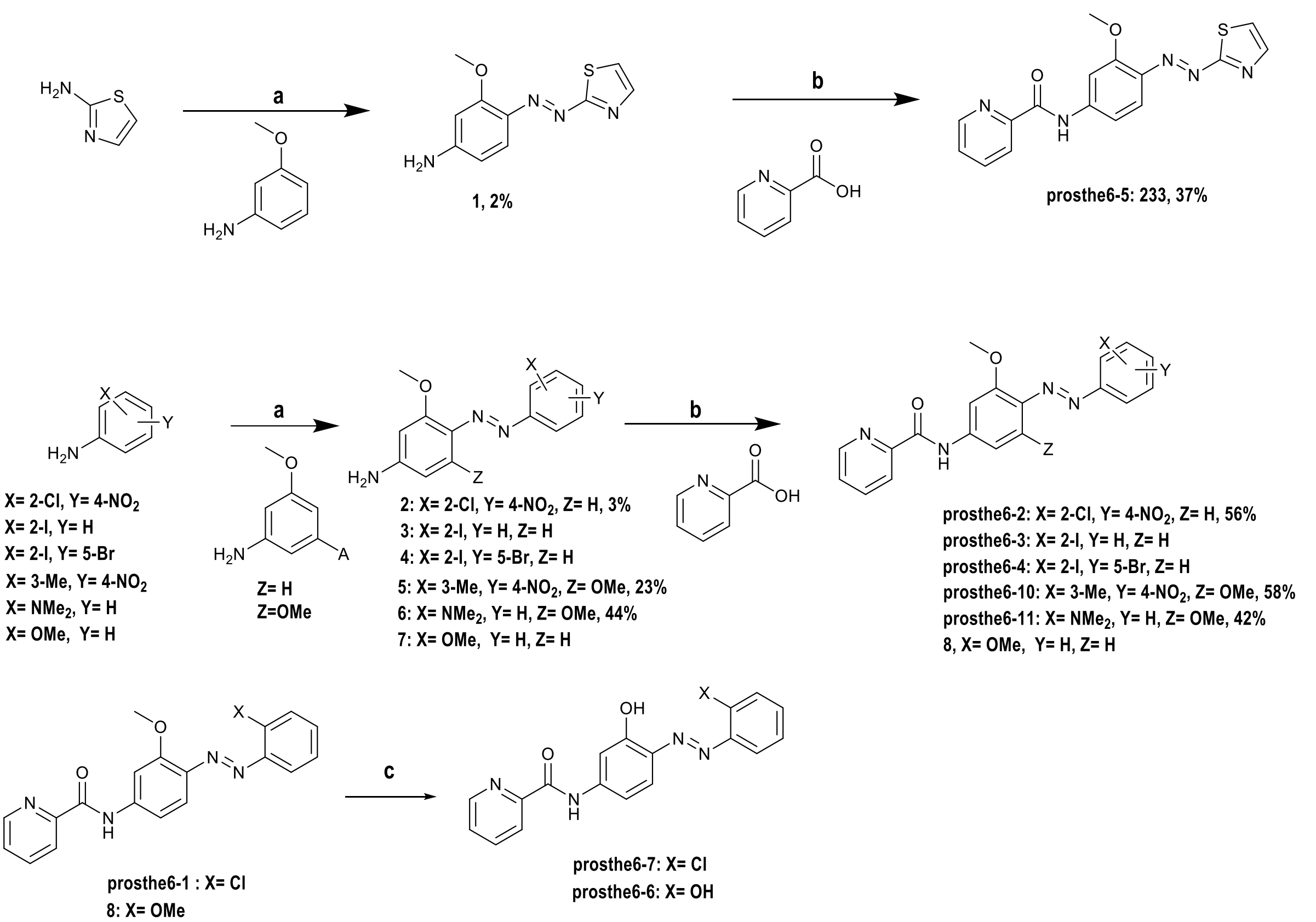

Figure S1. (**a**) (I) $NaNO_2$, aq HCl, 0 ºC, 10-120 min; (II) NaOAc, 0 ºC to rt, 30 min; (**b**) HATU, TEA, DMF, room temperature - 40 ºC, 18-72 h; (**c**) BBr_3, -78 ºC for 1 h then rt, 18 h.

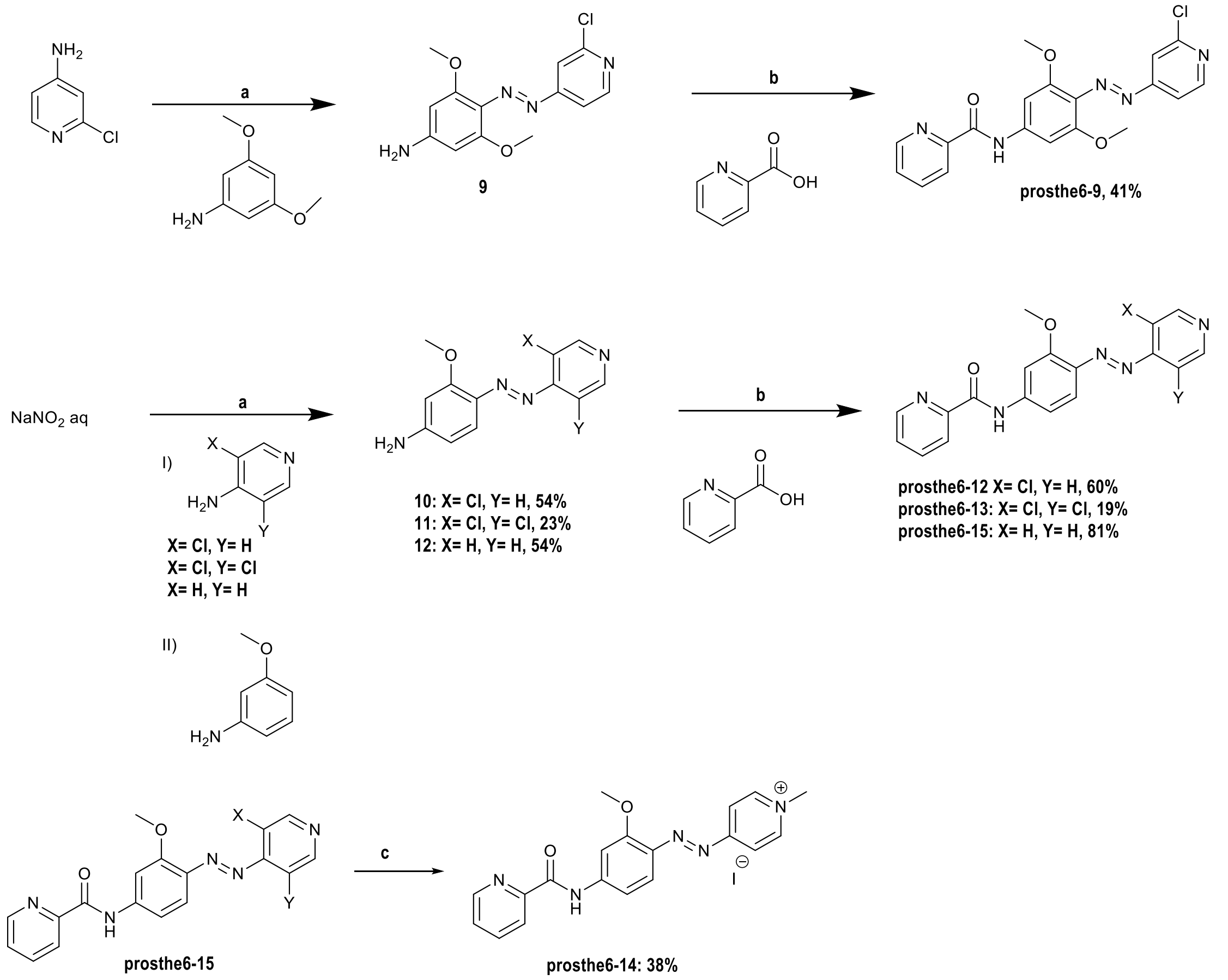

Figure S2. (**a**) (I) $H_3PO_{4conc}/HNO_{3conc}$ 2:1, 0-5 ºC, 45 min; (II) water/H_3PO_{4conc} 2:1, 0-5 ºC, 1 h; (**b**) HATU, TEA, DMF, 40 ºC, 18 h; (**c**) CH_3I, rt, 18 h.

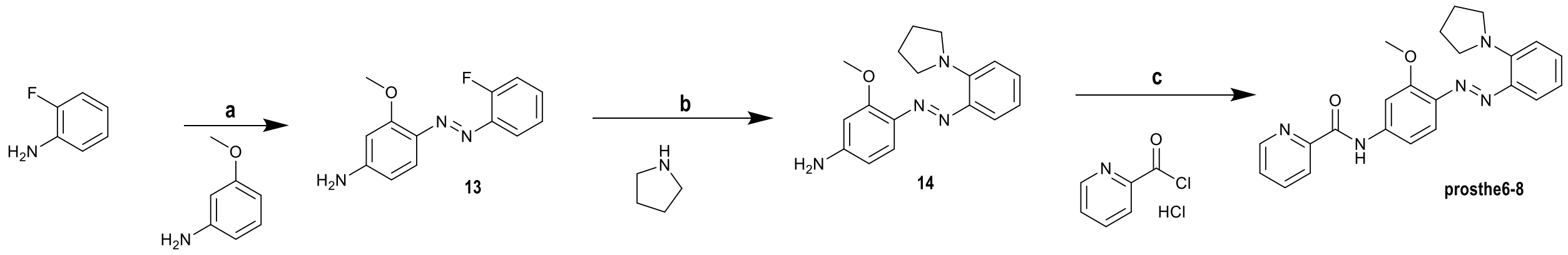

Figure S3. (**a**) (I) $NaNO_2$, aq HCl, 0 ºC, 10-120 min; (II) NaOAc, 0 ºC to rt, 30 min (**b**) 50 ºC, 48 h, 54 %; (**c**) DIPEA, dry DMF, 0 ºC to rt, 18 h, 94 %.

Materials and general methods

Abbreviations: ACN, Acetonitrile; AcOEt, Ethyl acetate; DCM, Dichloromethane; DMF, Dimethylformamide; ESI, Electrospray Ionization; FIA, Flux Injected Analysis; HATU, (1-[Bis(dimethylamino)methylene]-1H-1,2,3-triazolo[4,5-b]pyridinium 3-oxide hexafluorophosphate; HPLC, High Performance Liquid Chromatography; MeOH, Methanol; MS, Mass Spectrometry; m/z, mass/charge ratio; ND, Not determined; RT, retention time.

Chemicals and solvents: All the chemicals and solvents were provided from commercial suppliers and used without purification. Anhydrous solvents were obtained from solvent purification system (PureSolv-EN^{TM}) and preserved under inert atmosphere (nitrogen or argon).

Reaction monitoring: The reactions described below were monitored by thin layer chromatography (TLC) on silica gel (60F, 0.2 mm, ALUGRAM Sil G/UV_{254} *Macherey-Nagel*) and visualized under 254 nm Ultraviolet (UV) light.

Purification of compounds: Two different purification system were employed: silica-gel 60 (*Panreac,* 40-63 µm mesh) or Biotage Isolera One automated system with Biotage SNAP KP-C18-HS 50 µm columns and UV-Vis detection.

Melting points (mp): Melting points were measured with *Melting Point B-545* by *Büchi* set with auto detection and range modes.

Infrared-spectra (IR): IR spectra were recorded neat using *Thermo Nicolet Avatar 360 FT-IR* Spectrometer.

Nuclear magnetic resonance (NMR): Nuclear magnetic resonance (NMR) was performed on a 400 MHz Variant Mercury (*Agilent Technologies*) or on a 400MHz Brüker Avance NEO instrument. Data was processed through *Mestre Nova V. 8.1* software (*Mestrelab Research*). Chemical shifts are reported in δ (ppm) relative to the residual non-deuterated solvent signal (Chloroform-*d* δ = 7.26 ppm (^{1}H), δ = 77.2 ppm (^{13}C); DMSO-*d6* δ = 3.33 ppm (^{1}H), δ = 39.5 ppm (^{13}C); Methanol-*d4* δ = 4.87, 3.31 ppm (^{1}H), δ = 49.3 ppm (^{13}C). The abbreviations to label multiplicities are the following: s=singlet, d=doublet, t=triplet, q=quadruplet, m=multiplet, br=broad signal, dd=doublet of doublet, ddd=doublet of doublet of doublet, dt=doublet of triplet, qd=quadruplet of doblet. Coupling constants (*J*) are reported in Hz.

High performance liquid chromatography (HPLC) and mass spectrometry (MS): Compounds were analysed by analytical High-Performance Liquid Chromatography (HPLC) on a *Waters 2795 Alliance* coupled to a DAD detector (*Agilent* 1100) and an *ESI Quattro Micro* MS detector (*Waters*). HPLC column used was *ZORBAX Extend-C18* (2.1 x 50 mm, 3.5 µm). HPLC purity was determined using the following binary solvent system (named Method A from hereafter): 5 % acetonitrile in 0.05 % formic acid for 0.5 min, from 5 to 100 % acetonitrile in 5 min, 100 % acetonitrile for 1.5 min, from 100 to 5 % acetonitrile in 2 min and 5 % acetonitrile for 2 min. The flow rate was 0.5 mL/min, column temperature was fixed to 35 ºC and wavelengths from 210-600 nm were registered. Data from mass and UV-Vis spectra were analysed using *MassLynx* 4.1 software (*Waters*). The calculated purities from the compounds refer to the percentage peak area of the analysed compound by UV detection at 254 nm.

High-resolution mass spectrometry (HRMS): High-resolution mass spectra (HRMS) and elemental compositions were performed on a FIA (Flux Injected Analysis) with ultra-high-performance liquid chromatography (UPLC) *Aquity* (*Waters*) coupled to LCT Premier Orthogonal Accelerated Time of Flight Mass Spectrometer (TOF). Data from mass spectra was analysed by electrospray ionization in positive and negative modes using *MassLynx* 4.1. software (*Waters*). Spectra was scanned between 50 and 1500 Da with values every 0.2 s, peaks are reported as m/z. The required masses were obtained in the elemental composition analysis tool in the aforementioned software.

Synthesis of Intermediates

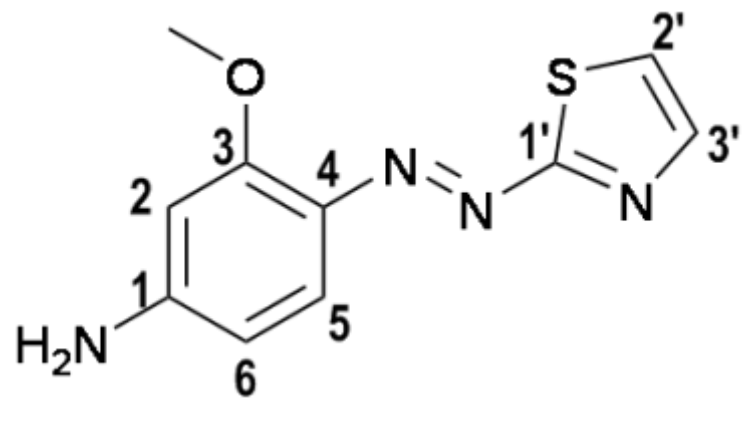

(*E*)-3-Methoxy-4-(thiazol-2-yldiazenyl)aniline 1. To a stirred solution of thiazol-2-amine (500 mg, 4.99 mmol) in MeOH (15.0 mL) in an ice bath, HCl_{conc} (1.30 mL, 15.5 mmol) was added dropwise while keeping the temperature between 0-5 ºC. Then, an aqueous solution of sodium nitrite (344 mg, 4.99 mmol) in water (1.8 mL) was added dropwise to the mixture to generate the azonium intermediate and it was left stirring for 2 h. In a separate reaction vessel, a solution of 3-methoxyaniline (0.68 mL, 5.99 mmol) and sodium acetate (1.270 g, 15.5 mmol) in MeOH (15.0 mL) was prepared and cooled in an ice bath. The diazonium solution was added to the 3-methoxyaniline solution in dropwise fashion while keeping the temperature between 0-5 ºC and then the resulting mixture was left stirring for 18

h. Afterwards, the reaction mixture was partitioned with EtOAc and saturated aqueous $NaHCO_3$. The aqueous phase was back extracted with EtOAc and the combined organic layers were washed with water (2x) and then brine. The organic phase was dried over anhydrous $MgSO_4$, filtered and concentrated under reduced pressure. The crude was purified by *flash* column chromatography (Hexanes/EtOAc 50:50 to 50:50) and reverse phase automated column chromatography (Water/ACN 95:5 to ACN + 0.05 % HCOOH) to give the title compound as a dark red solid (27.8 mg, 0.119 mmol, 2 %). ν_{max}/cm^{-1} 3327 (N-H), 3094 (C-H), 2919 (C-H), 1593 (N=N). ^{1}H NMR (400 MHz, Chloroform-*d*) δ 7.93 - 7.88 (m, 2H, C(3')*H*, C(5)*H*), 7.28 - 7.21 (m, 1H, C(2')*H*), 6.29 (dd, *J* = 8.9, 2.3 Hz, 1H, C(6)*H*), 6.24 (d, *J* = 2.3 Hz, 1H, C(2)*H*), 4.34 (br s, 1H, NH_2), 3.98 (s, 3H, OCH_3). ^{13}C NMR (101 MHz, Chloroform-*d*) δ 179.1 (*C*(1')), 160.7 (*C*(3)), 153.9 (*C*(1)), 143.1 (*C*(3')), 134.4 (*C*(4)), 120.4 (*C*(5)), 119.3 (*C*(2')), 108.3 (*C*(6)), 97.4 (*C*(2)), 56.5 (OCH_3). HPLC-PDA-MS: method A was used; RT: 3.00 min, *m/z* (ESI^+) 235 (MH^+), λ_{max} = 486 nm (*trans* isomer); purity 92 % (254 nm). HRMS (ESI^+) found 235.0653, $C_{10}H_{11}N_4OS$ (MH^+) requires 235.0654.

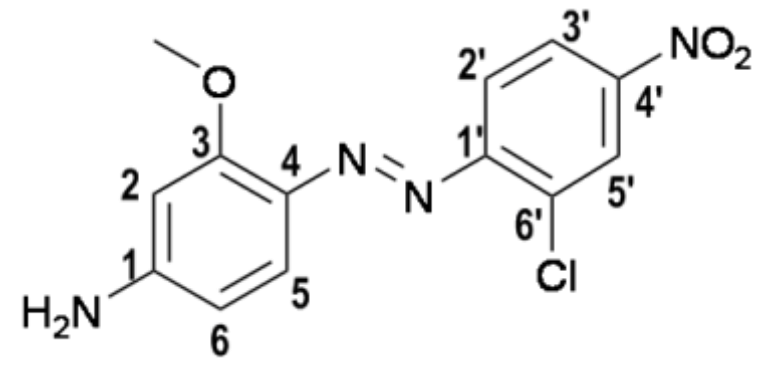

(*E*)-4-((2-Chloro-4-nitrophenyl)diazenyl)-3-methoxyaniline 2. To a stirred solution of 2-chloro-4-nitroaniline (500 mg, 2.90 mmol) in MeOH (15.0 mL) in an ice bath, HCl_{conc} (0.74 mL, 8.98 mmol) was added dropwise while keeping the temperature between 0-5 ºC. Then, an aqueous solution of sodium nitrite (200 mg, 2.90 mmol) in water (1.8 mL) was added dropwise to the mixture to generate the azonium intermediate and it was left stirring for 45 min. In a separate reaction vessel, a solution of 3-methoxyaniline (0.40 mL, 3.48 mmol) and sodium acetate (737 mg, 8.98 mmol) in MeOH (15.0 mL) was prepared and cooled in an ice bath. The diazonium solution was added to the 3-methoxyaniline solution in dropwise fashion while keeping the temperature between 0-5 ºC and then the resulting mixture was left stirring for 18 h. Afterwards, the reaction mixture was partitioned with EtOAc and saturated aqueous $NaHCO_3$. The aqueous phase was backextracted with EtOAc and the combined organic layers were washed with water (2x) and then brine. The organic phase was dried over anhydrous $MgSO_4$, filtered and concentrated under reduced pressure. The crude was purified by *flash* column chromatography (Hexanes/EtOAc 80:20) and reverse phase automated column chromatography (Water/ACN 95:5 to ACN + 0.05 % HCOOH) to give the title compound as a dark red solid (30.1 mg, 0.098 mmol, 3 %). Mp: 204 - 205 ºC. ν_{max}/cm^{-1} 3338 (N-H), 2916 (C-H), 2849 (C-H), 1621 (N-H), 1596 (N=N). ^{1}H NMR (400 MHz, Chloroform-*d*) δ 8.36 (d, *J* = 2.4 Hz, 1H, C(5')*H*), 8.13 (dd, *J* = 8.9, 2.4 Hz, 1H, C(3')*H*), 7.83 (d, *J* = 8.7 Hz, 1H, C(5)*H*), 7.74 (d, *J* = 8.9 Hz, 1H, C(2')*H*), 6.32 - 6.27 (m, 2H, C(2)*H*, C(6)*H*), 4.34 (br s, 2H, NH_2), 3.99 (s, 3H, OCH_3). ^{13}C NMR (101 MHz, Chloroform-*d*) δ 161.1 (*C*(3)), 154.0 (*C*(1)), 153.7 (*C*(4')), 147.2 (*C*(1')), 136.0 (*C*(4)), 133.8 (*C*(6')), 126.0 (*C*(5')), 122.8 (*C*(3')), 119.9 (*C*(5)), 118.7 (*C*(2')), 108.1 (*C*(6)), 97.1 (*C*(2)), 56.3 (OCH_3). HPLC-PDA-MS: method A was used; RT: 3.61 min, *m/z* (ESI^+) 307 (MH^+), λ_{max} = 466 nm (*trans* isomer); purity 97 % (254 nm). HRMS (ESI^+) found 307.0594, $C_{13}H_{12}N_4O_3Cl$ (MH^+) requires 307.0598.

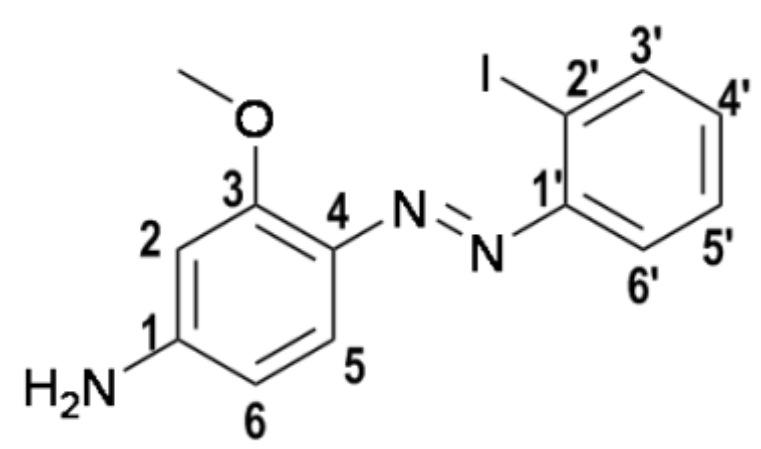

(*E*)-4-((2-Iodophenyl)diazenyl)-3-methoxyaniline 3. To a stirred solution of 2-iodoaniline (500 mg, 2.28 mmol) in MeOH (15.0 mL) in an ice bath, HCl_{conc} (0.58 mL, 7.08 mmol) was added dropwise while keeping the temperature between 0-5 ºC. Then, an aqueous solution of sodium nitrite (158 mg, 2.28 mmol) in water (1.8 mL) was added dropwise to the mixture to generate the azonium intermediate and it was left stirring for 45 min. In a separate reaction vessel, a solution of 3-methoxyaniline (0.30 mL, 2.74 mmol) and sodium acetate (581 mg, 7.08 mmol) in MeOH (15.0 mL) was prepared and cooled in an ice bath. The diazonium solution was added to the 3-methoxyaniline solution in dropwise fashion while keeping the temperature between 0-5 ºC and then the resulting mixture was left stirring for 18 h. Afterwards, the reaction mixture was partitioned with EtOAc and saturated aqueous $NaHCO_3$. The aqueous phase was backextracted with EtOAc and the combined organic layers were washed with water (2x) and then brine. The organic phase was dried over anhydrous $MgSO_4$, filtered, and concentrated under reduced pressure. The crude was purified by *flash* column chromatography (Hexanes/EtOAc 90:10 to 70:30) to give the title compound as a red solid (345 mg, 0.977 mmol, 43 %). Mp: 201 – 202 ºC. ν_{max}/cm^{-1} 3473 (N-H), 2969 (C-H), 2932 (C-H), 1621 (N-H), 1588 (N=N). ^{1}H NMR (400 MHz, Chloroform-*d*) δ 7.95 (dd, J = 7.9, 1.3 Hz, 1H, C(3')*H*), 7.83 (dd, *J* = 8.3, 0.7 Hz, 1H, C(5)*H*), 7.58 (dd, *J* = 8.0, 1.6 Hz, 1H, C(6')*H*), 7.41 - 7.34 (m, 1H, C(5')*H*), 7.05 (ddd, *J* = 7.9, 7.2, 1.6 Hz, 1H, C(4')*H*), 6.34 - 6.27 (m, 2H, C(2)*H*, C(6)*H*), 4.16 (br s, 2H, N*H₂*), 3.98 (s, 3H, OC*H₃*). ^{13}C NMR (101 MHz, Chloroform-*d*) δ 159.8 (*C*(3)), 152.3 (*C*(1')), 152.2 (*C*(1)), 139.5 (*C*(3')), 135.2 (*C*(4)), 130.7 (*C*(4')), 129.0 (*C*(5')), 119.7 (*C*(5)), 117.9 (*C*(6')), 107.8 (*C*(6)), 101.3 (*C*(2')), 97.7 (*C*(2)), 56.3 (O*C*H₃). HPLC-PDA-MS: method A was used; RT: 3.61 min, *m/z* (ESI⁺) 354 (MH⁺), λ_{max} = 460 nm (*trans* isomer); purity >99 % (254 nm). HRMS (ESI⁺) found 354.0089, $C_{13}H_{13}N_3OI$ (MH⁺) requires 354.0103.

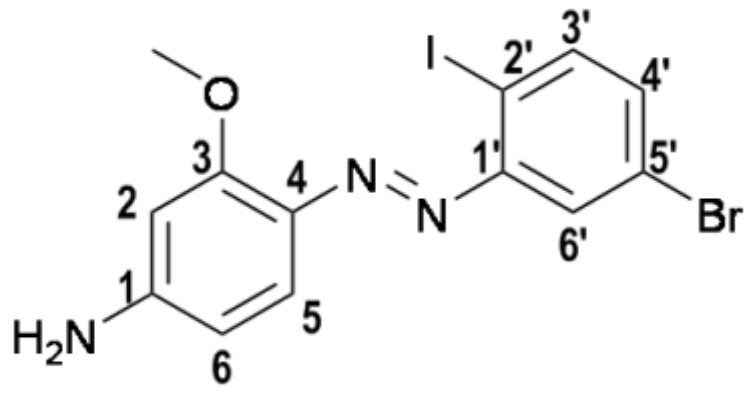

(*E*)-4-((5-Bromo-2-iodophenyl)diazenyl)-3-methoxyaniline 4. To a stirred solution of 4-bromo-2-iodoaniline (500 mg, 1.67 mmol) in MeOH (15.0 mL) in an ice bath, HCl_{conc} (0.42 mL, 5.20 mmol) was added dropwise while keeping the temperature between 0-5 ºC. Then, an aqueous solution of sodium nitrite (116 mg, 1.68 mmol) in water (1.8 mL) was added dropwise to the mixture to generate the azonium intermediate and it was left stirring for 45 min. In a separate reaction vessel, a solution of 3-methoxyaniline (0.26 mL, 2.01 mmol) and sodium acetate (427 mg, 5.20 mmol) in MeOH (15.0 mL) was prepared and cooled in an ice bath. The diazonium solution was added to the 3-methoxyaniline solution in dropwise fashion while keeping the temperature between 0-5 ºC and then the resulting mixture was left stirring for 18 h. Afterwards, the reaction mixture was partitioned with EtOAc and saturated aqueous $NaHCO_3$. The aqueous phase was backextracted with EtOAc and the combined organic layers were washed with water (2x) and then brine. The organic phase was dried over anhydrous $MgSO_4$, filtered and concentrated under reduced pressure. The crude was purified by *flash* column chromatography (Hexanes/EtOAc 90:10 to 70:30) to give the title compound as a dark red solid (199 mg, 0.460 mmol, 27 %). Mp: 155 – 156 ºC. ν_{max}/cm^{-1} 3460 (N-H), 2922 (C-H), 1621 (N-H), 1588 (N=N). ^{1}H NMR (400 MHz, Chloroform-*d*) δ 8.09 (d, *J* = 1.7 Hz, 1H, C(6')*H*), 7.82 (d, *J* = 8.6 Hz, 1H, C(5)*H*), 7.50 - 6.63 (m, 2H, C(3')*H*, C(4')*H*), 6.33 - 6.25 (m, 2H, C(2)*H*, C(6)*H*), 4.20 (br s, 2H, N*H₂*), 3.98 (s, 3H, OC*H₃*). ^{13}C NMR (101 MHz, Chloroform-*d*) δ 160.0 (*C*(3)), 152.6 (*C*(1')), 151.2 (*C*(1)), 141.4 (*C*(6')), 135.1 (*C*(4)), 132.1 (*C*(4')), 123.6 (*C*(5')), 119.8 (*C*(5)), 118.7 (*C*(3')), 107.9 (*C*(6)), 101.9 (*C*(2')), 97.6 (*C*(2)), 56.3 (O*C*H₃). HPLC-PDA-MS: method A was used; RT: 4.31 min, *m/z* (ESI⁺) 432 (MH⁺), λ_{max} = 432 nm (*trans* isomer); purity >99 % (254 nm). HRMS (ESI⁺) found 431.9221, $C_{13}H_{12}N_3OBrI$ (MH⁺) requires 431.9209.

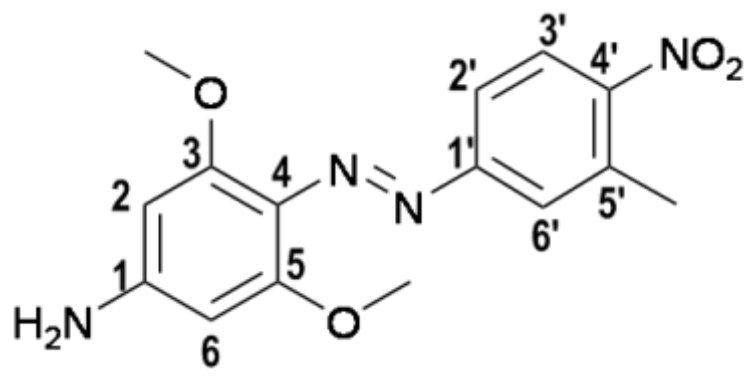

(*E*)-3,5-Dimethoxy-4-((3-methyl-4-nitrophenyl)diazenyl)aniline 5. To a stirred solution of 3-methyl-4-nitroaniline (210 mg, 1.38 mmol) in water (7.5 mL) in an ice bath, HCl_{conc} (0.36 mL, 4.39 mmol) was added dropwise while keeping the temperature between 0-5 ºC. Then, an aqueous solution of sodium nitrite (95.0 mg, 1.38 mmol) in water (1.8 mL) was added dropwise to the mixture to generate the azonium intermediate and it was left stirring for 45 min. In a separate reaction vessel, a solution of 3,5-dimethoxyaniline (254 mg, 1.66 mmol) and sodium acetate (351 mg, 4.28 mmol) in water (7.5 mL) was prepared and cooled in an ice bath. The diazonium solution was added to the 3,5-dimethoxyaniline solution in dropwise fashion while keeping the temperature between 0-5 ºC and then the resulting mixture was left stirring for 18 h. Afterwards, the reaction mixture was partitioned with EtOAc and saturated aqueous $NaHCO_3$. The aqueous phase was backextracted with EtOAc and the combined organic layers were washed with water (2x) and then brine. The organic phase was dried over anhydrous $MgSO_4$, filtered and concentrated under reduced pressure. The crude was purified by *flash* column chromatography (Hexanes/EtOAc 50:50 to EtOAc) to give the title compound as a dark red solid (100 mg, 0.316 mmol, 23 %). Mp: 184 - 185 ºC . ν_{max}/cm^{-1} 3435 (N-H), 3333 (N-H), 2918 (C-H), 2848 (C-H), 1650 (N-H), 1598 (N=N). ^{1}H NMR (400 MHz, DMSO-*d6*) δ 8.09 (d, *J* = 8.8 Hz, 1H, C(3')*H*), 7.58 (dd, *J* = 2.1, 0.9 Hz, 1H, C(6')*H*), 7.52 (dd, *J* = 8.8, 2.1 Hz, 1H, C(2')*H*), 6.53 (br s, 2H, N*H*$_2$), 5.97 (s, 2H, C(2)*H*, C(6)*H*), 3.77 (s, 6H, 2·OC*H*$_3$), 2.60 (s, 3H, C*H*$_3$). ^{13}C NMR (101 MHz, DMSO-*d6*) δ 157.5 (*C*(3), *C*(5)), 157.0 (*C*(1')), 155.6 (*C*(1)), 146.7 (*C*(4')), 134.6 (*C*(5')), 126.0 (*C*(3')), 125.5 (*C*(6')), 123.6 (*C*(4)), 118.3 (*C*(2')), 89.7 (*C*(2), *C*(6)), 55.6 (2·O*C*H$_3$), 20.4 (*C*H$_3$). HPLC-PDA-MS: method A was used; RT: 3.05 min, *m/z* (ESI^+) 317 (MH^+), λ_{max} = 448 nm (*trans* isomer); purity 91 % (254 nm). HRMS (ESI^+) found 317.1250, $C_{15}H_{17}N_4O_4$ (MH^+) requires 317.1250.

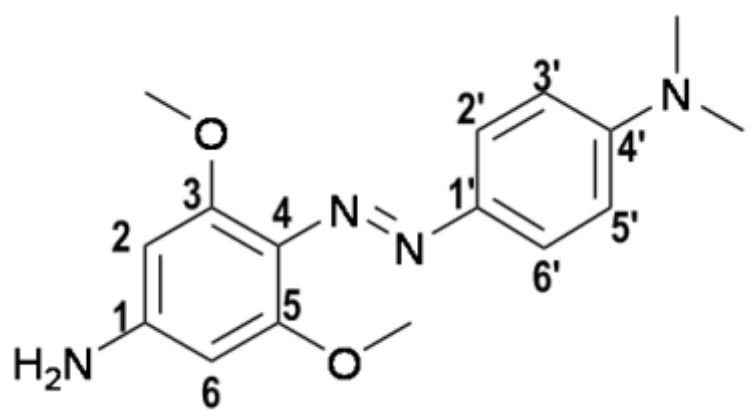

(*E*)-4-((4-Amino-2,6-dimethoxyphenyl)diazenyl)-N,N-dimethylaniline 6. To a stirred solution of *N1*,*N1*-dimethylbenzene-1,4-diamine (200 mg, 1.47 mmol) in water (7.5 mL) in an ice bath, HCl_{conc} (0.36 mL, 4.39 mmol) was added dropwise while keeping the temperature between 0-5 ºC. Then, an aqueous solution of sodium nitrite (107 mg, 1.56 mmol) in water (1.8 mL) was added dropwise to the mixture to generate the azonium intermediate and it was left stirring for 45 min. In a separate reaction vessel, a solution of 3,5-dimethoxyaniline (270 mg, 1.76 mmol) and sodium acetate (373 mg, 4.55 mmol) in water (7.5 mL) was prepared and cooled in an ice bath. The diazonium solution was added to the 3,5-dimethoxyaniline solution in dropwise fashion while keeping the temperature between 0-5 ºC and then the resulting mixture was left stirring for 18 h. Afterwards, the reaction mixture was partitioned with EtOAc and saturated aqueous $NaHCO_3$. The aqueous phase was backextracted with EtOAc and the combined organic layers were washed with water (2x) and then brine. The organic phase was dried over anhydrous $MgSO_4$, filtered and concentrated under reduced pressure. The crude was purified by *flash* column chromatography (Hexanes/EtOAc 50:50 to 20:80) to give the title compound as a dark red solid (196 mg, 0.653 mmol, 44 %). ν_{max}/cm^{-1} 3348 (N-H), 3219 (N-H), 2967 (C-H), 2900 (C-H), 1593 (N=N). ^{1}H NMR (400 MHz, DMSO-*d6*) δ 7.53 (d, *J* = 9.1 Hz, 2H, C(2')*H*, C(6')*H*), 6.77 (d, *J* = 9.1 Hz, 2H, C(3')*H*, C(5')*H*), 5.95 (s, 2H, C(2)*H*, C(6)*H*), 5.70 (br s, 2H, N*H*$_2$), 3.69 (s, 6H, 2·OCH$_3$), 2.99 (s, 6H, 2·*C*H$_3$). ^{13}C NMR (101 MHz, DMSO-*d6*) δ 154.8 (*C*(3), *C*(5)), 151.0 (*C*(1)), 150.9 (*C*(4')), 144.7 (*C*(1')), 123.7 (*C*(4)), 122.9 (*C*(2'), *C*(6')), 111.6 (*C*(3'), *C*(5')), 87.2 (*C*(2), *C*(6)), 55.6 (2·O*C*H$_3$), 40.3 (2·*C*H$_3$).

HPLC-PDA-MS: method A was used; RT: 2.94 min, *m/z* (ESI^+) 301 (MH^+), λ_{max} = 442, 570 nm (*trans* isomer); purity 90 % (254 nm). HRMS (ESI^+) found 301.1659, $C_{16}H_{21}N_4O_2$ (MH^+) requires 301.1655.

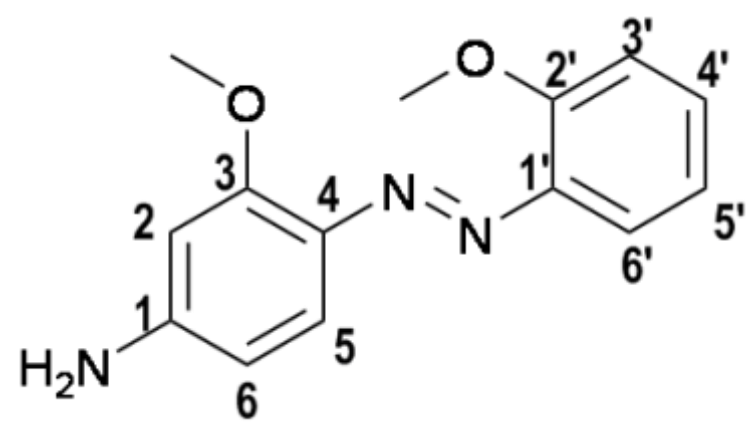

(*E*)-3-Methoxy-4-((2-methoxyphenyl)diazenyl)aniline 7. To a stirred solution of 2-methoxyaniline (0.46 mL, 4.1 mmol) in MeOH (7.5 mL) in an ice bath, HCl_{conc} (1.00 mL, 12.2 mmol) was added dropwise while keeping the temperature between 0-5 ºC. Then, an aqueous solution of sodium nitrite (336 mg, 4.87 mmol) in water (1.8 mL) was added dropwise to the mixture to generate the azonium intermediate and it was left stirring for 10 min. In a separate reaction vessel, a solution of 3-methoxyaniline (0.55 mL, 4.98 mmol) and sodium acetate (1.03 g, 12.0 mmol) in MeOH/H_2O (2:1 v/v, 12 mL) was prepared and cooled in an ice bath. The diazonium solution was added to the 3-methoxyaniline solution in dropwise fashion while keeping the temperature between 0-5 ºC and then the resulting mixture was left stirring for another 30 min. Afterwards, water was added dropwise to induce the precipitacion of a red solid which was isolated by filtration and washed with water. The red solid was disolved using EtOAc and then dried over anhydrous $MgSO_4$, filtered, and concentrated under reduced pressure. The crude was purified by *flash* column chromatography (Hexanes/EtOAc 60:40 to 20:80) to give the title compound as a dark red solid (630 mg, 2.45 mmol, 60 %). Mp: 202 - 203 ºC. ν_{max}/cm^{-1} 3355 (N-H), 3221 (C-H), 2921 (C-H), 1625 (N-H),1600 (N=N). ^1H NMR (400 MHz, Chloroform-*d*) δ 7.69 (d, *J* = 8.4 Hz, 1H, C(5)*H*), 7.61 (d, *J* = 7.8 Hz, 1H, C(6')*H*), 7.34 (td, *J* = 7.8, 5.2 Hz, 1H, C(5')*H*), 7.08 - 6.94 (m, 2H, C(3')*H*, C(4')*H*), 6.33 - 6.24 (m, 2H, C(2)*H*, C(6)*H*), 4.06 (s, 3H, OCH_3), 3.99 (s, 3H, OCH_3'). ^{13}C NMR (101 MHz, Chloroform-*d*) δ 159.9 (*C*(2')), 156.1 (*C*(3)), 151.2 (*C*(1)), 142.7 (*C*(1')), 136.7 (*C*(4)), 130.7 (*C*(5')), 120.9 (*C*(4')), 119.0 (*C*(5)), 117.5 (*C*(6')), 112.3 (*C*(3')), 107.4 (*C*(6)), 97.9 (*C*(2)), 56.30 (OCH_3), 56.15 (OCH_3'). HPLC-PDA-MS: method A was used; RT: 2.75 min, *m/z* (ESI^+) 258 (MH^+), λ_{max} = 486 nm (*trans* isomer); purity 94 % (254 nm). HRMS (ESI^+) found 258.1237, $C_{14}H_{16}N_3O_2$ (MH^+) requires 258.1243.

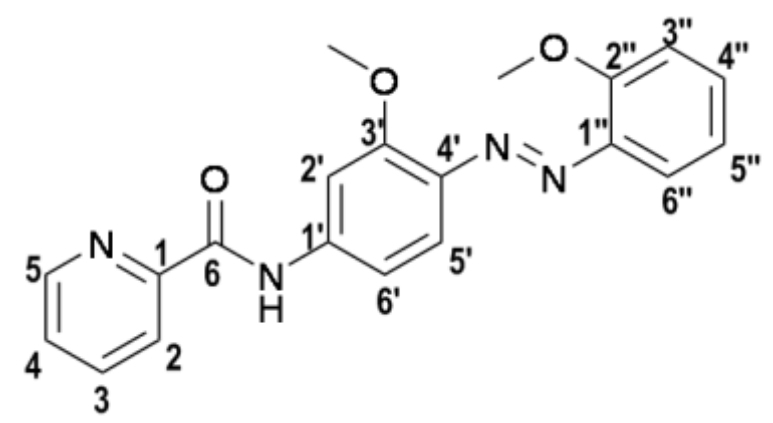

(*E*)-N-(3-Methoxy-4-((2-methoxyphenyl)diazenyl)phenyl)picolinamide 8. To a stirred solution of azocompound 7 (25.0 mg, 0.097 mmol), picolinic acid (17.9 mg, 0.146 mmol) and HATU (111 mg, 0.292 mmol) in DMF (2.0 mL), TEA (81 µL, 0.583 mmol) was added and then left stirring 17 h at 40 ºC. Afterwards, the reaction mixture was partitioned with EtOAc and saturated aqueous Na_2CO_3. The aqueous phase was backextracted with EtOAc and the combined organic layers were washed with water, saturated aqueous $NaHCO_3$, water (10x) and then brine. The organic phase was dried over anhydrous $MgSO_4$, filtered and concentrated under reduced pressure. The crude consisting of an orange solid (35.0 mg, 0.097 mmol, 99 %) was obtained pure and no purification was required. Mp: 164 - 165 ºC. ν_{max}/cm^{-1} 3323 (N-H), 3131 (C-H), 2919 (C-H), 1683 (C=O),1584 (N=N). ^1H NMR (400 MHz, Chloroform-*d*) δ 10.23 (s, 1H, C(6)ON*H*), 8.62 (d, *J* = 4.7 Hz, 1H, C(5)*H*), 8.30 (dt, *J* = 7.7, 1.2 Hz, 1H, C(2)*H*), 8.05 (d, *J* = 2.1 Hz, 1H, C(2')*H*), 7.93 (td, *J* = 7.7, 1.7 Hz, 1H, C(3)*H*), 7.76 (d, *J* = 8.7 Hz, 1H, C(5')*H*), 7.65 (dd, *J* = 8.0, 1.7 Hz, 1H, C(6'')*H*), 7.50 (ddd, *J* = 7.7, 4.7, 1.2 Hz, 1H, C(4)*H*), 7.39 (ddd, *J* = 8.4, 8.0, 1.7 Hz, 1H, C(5'')*H*), 7.11 (dd, *J* = 8.7, 2.1 Hz, 1H, C(6')*H*), 7.06 (dd, *J* = 8.4, 1.7 Hz, 1H, C(3'')*H*), 7.03 -

6.97 (m, 1H, C(4'')*H*), 4.09 (s, 3H, OCH_3), 4.01 (s, 3H, OCH_3'). ^{13}C NMR (101 MHz, Chloroform-*d*) δ 162.1 (*C*(6)), 158.0 (*C*(2'')), 156.6 (*C*(3')), 149.4 (*C*(1)), 148.0 (*C*(5)), 143.0 (*C*(1')), 141.6 (*C*(1'')), 139.3 (C(4')), 137.8 (*C*(3)), 131.8 (*C*(5'')), 126.7 (*C*(4)), 122.4 (*C*(2)), 120.8 (*C*(4'')), 118.1 (*C*(5')), 117.5 (*C*(6'')), 112.4 (*C*(3'')), 111.6 (*C*(6')), 103.4 (*C*(2')), 56.3 (OCH_3), 56.3 (OCH_3'). HPLC-PDA-MS: method A was used; RT: 3.51 min, *m/z* (ESI$^+$) 363 (MH$^+$), λ_{max} = 434 nm (*cis* isomer) ; RT: 3.87 min, *m/z* (ESI$^+$) 363 (MH+), λ_{max} = 386 nm (*trans* isomer); purity >99 % (254 nm). HRMS (ESI$^+$) found 363.1429, $C_{20}H_{19}N_4O_3$ (MH$^+$) requires 369.1457.

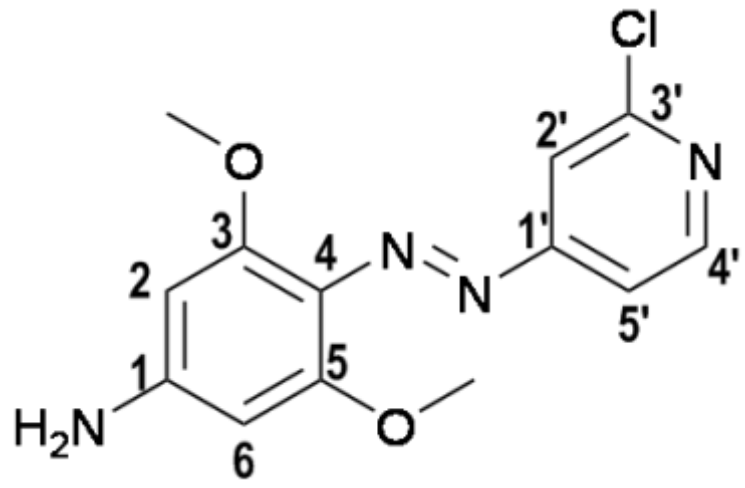

(*E*)-4-((2-Chloropyridin-4-yl)diazenyl)-3,5-dimethoxyaniline 9. To a stirred solution of 2-chloropyridin-4-amine (200 mg, 1.56 mmol) in water (7.5 mL) in an ice bath, HCl_{conc} (0.40 mL, 4.82 mmol) was added dropwise while keeping the temperature between 0-5 ºC. Then, an aqueous solution of sodium nitrite (107 mg, 1.56 mmol) in water (1.8 mL) was added dropwise to the mixture to generate the azonium intermediate and it was left stirring for 45 min. In a separate reaction vessel, a solution of 3,5-dimethoxyaniline (286 mg, 1.87 mmol) and sodium acetate (396 mg, 4.82 mmol) in water (7.5 mL) was prepared and cooled in an ice bath. The diazonium solution was added to the 3,5-dimethoxyaniline solution in dropwise fashion while keeping the temperature between 0-5 ºC and then the resulting mixture was left stirring for 18 h. Afterwards, the reaction mixture was partitioned with EtOAc and saturated aqueous $NaHCO_3$. The aqueous phase was backextracted with EtOAc and the combined organic layers were washed with water (2x) and then brine. The organic phase was dried over anhydrous $MgSO_4$, filtered and concentrated under reduced pressure. The crude was purified by *flash* column chromatography (Hexanes/EtOAc 50:50 to 20:80) to give the title compound as a red solid (149 mg, 0.509 mmol, 33 %). ν_{max}/cm^{-1} 3325 (N-H), 3094 (C-H), 2921 (C-H), 2850 (C-H), 1606 (N-H), 1577 (N=N). ^{1}H NMR (400 MHz, DMSO-*d6*) δ 8.39 (d, *J* = 5.4 Hz, 1H, C(4')*H*), 7.45 (dd, *J* = 5.4, 1.7 Hz, 1H, C(5')*H*), 7.40 (d, *J* = 1.7 Hz, 1H, C(2')*H*), 6.76 (br s, 2H, NH_2), 5.97 (s, 2H, C(2)*H*, C(6)*H*), 3.78 (s, 6H, 2·OCH_3). ^{13}C NMR (101 MHz, DMSO-*d6*) δ 162.7 (*C*(1')), 158.2 (*C*(3), *C*(5)), 156.8 (*C*(1)), 151.1 (*C*(3')), 150.6 (*C*(4')), 123.4 (*C*(4)), 115.6 (*C*(5')), 114.2 (*C*(2')), 89.6 (*C*(2), *C*(6)), 55.6 (2·OCH_3). HPLC-PDA-MS: method A was used; RT: 2.75 min, *m/z* (ESI$^+$) 293 (MH$^+$), λ_{max} = 416 nm (*trans* isomer); purity 91 % (254 nm). HRMS (ESI$^+$) found 293.0797, $C_{13}H_{14}N_4O_2Cl$ (MH$^+$) requires 293.0805.

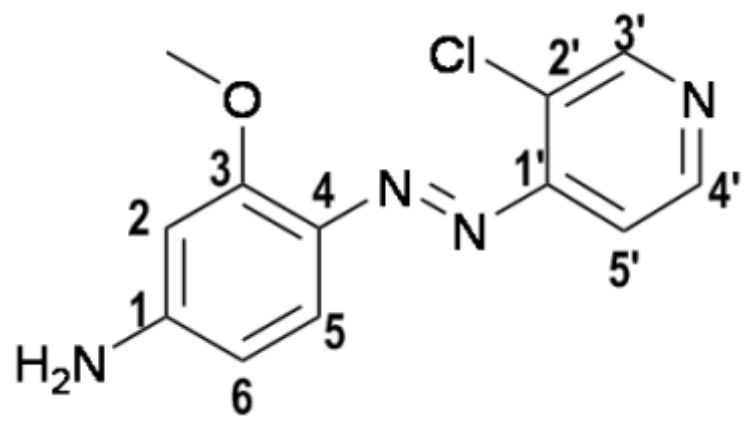

(*E*)-4-((3-Chloropyridin-4-yl)diazenyl)-3-methoxyaniline 10. To a stirred solution of sodium nitrite (129 mg, 1.87 mmol) in water (4.0 mL) at 0 ºC, an ice-cold solution of 3-chloropyridin-4-amine (200 mg, 1.56 mmol) in H_3PO_{4conc}/HNO_{3conc} 2:1 (20.0 mL) was added slowly keeping the temperature between 0-5 ºC. The solution was stirred at 0-5ºC for 45 min. Then, it was added dropwise into a solution of 3-methoxyaniline (0.22 mL, 1.96 mmol) in water/H_3PO_{4conc} 2:1 (20.0 mL) keeping temperature of the reaction at 0-5 ºC. It was left 1h from 0-5ºC to room temperature. Afterwards, the reaction mixture was partitioned with EtOAc and saturated aqueous Na_2CO_3. The aqueous phase was backextracted with EtOAc and the combined organic layers were washed with water, saturated aqueous $NaHCO_3$, water (10x) and then brine. The organic phase was dried over anhydrous $MgSO_4$,

filtered, and concentrated under reduced pressure. The crude was purified by *flash* column chromatography (Hexanes/EtOAc 50:50) to give the title compound as a dark red solid (222 mg, 0.845 mmol, 54 %). ν_{max}/cm^{-1} 3430 (N-H), 3226 (N-H), 2980 (C-H), 2919 (C-H), 1594 (N=N). ^{1}H NMR (400 MHz, Chloroform-*d*) δ 8.68 (s, 1H, C(3')*H*), 8.49 (d, *J* = 5.3 Hz, 1H, C(4')*H*), 7.83 (d, *J* = 8.6 Hz, 1H, C(5)*H*), 7.44 (d, *J* = 5.3 Hz, 1H, C(5')*H*), 6.39 - 6.11 (m, 2H, C(2)*H*, C(6)*H*), 4.34 (br s, 2H, N*H₂*), 3.99 (s, 3H, OCH_3). ^{13}C NMR (101 MHz, Chloroform-*d*) δ 161.0 (*C*(3)), 154.9 (*C*(1')), 153.9 (*C*(1)), 151.1 (*C*(3')), 148.8 (*C*(4')), 135.9 (*C*(4)), 129.8 (*C*(2')), 119.8 (*C*(5)), 112.1 (*C*(5')), 108.0 (*C*(6)), 97.2 (*C*(2)), 56.3 (OCH_3). HPLC-PDA-MS: method A was used; RT: 2.67 min, *m/z* (ESI$^+$) 263 (MH$^+$), λ_{max} = 428 nm (*trans* isomer); purity 75 % (254 nm). HRMS (ESI$^+$) found 263.0687, $C_{12}H_{12}N_4OCl$ (MH$^+$) requires 263.0700.

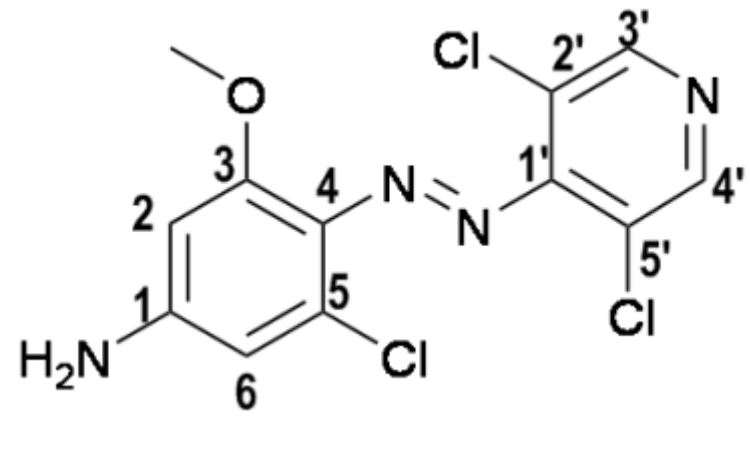

(*E*)-3-Chloro-4-((3,5-dichloropyridin-4-yl)diazenyl)-5-methoxyaniline 11. To a stirred solution of sodium nitrite (102 mg, 1.47 mmol) in water (4.0 mL) at 0 ºC, an ice-cold solution of 3,5-dichloropyridin-4-amine (200 mg, 1.23 mmol) in H_3PO_{4conc}/HNO_{3conc} 2:1 (20.0 mL) was added slowly keeping the temperature between 0-5 ºC. The solution was stirred at 0-5ºC for 45 min. Then, it was added dropwise into a solution of 3-chloro-5-methoxyaniline (232 mg, 1.47 mmol) in water/H_3PO_{4conc} 2:1 (20.0 mL) keeping temperature of the reaction at 0-5 ºC. It was left 1h from 0-5ºC to room temperature. Afterwards, the reaction mixture was partitioned with EtOAc and saturated aqueous Na_2CO_3. The aqueous phase was backextracted with EtOAc and the combined organic layers were washed with water, saturated aqueous $NaHCO_3$, water (10x) and then brine. The organic phase was dried over anhydrous $MgSO_4$, filtered, and concentrated under reduced pressure. The crude was purified by *flash* column chromatography (Hexanes/EtOAc 50:50) and then (DCM/EtOAc 99.5:0.5) to give the title compound as a dark red solid (93.0 mg, 0.280 mmol, 23 %). Mp: 181 - 182 ºC. ν_{max}/cm^{-1} 3427 (N-H), 3224 (N-H), 2970 (C-H), 1634 (N-H), 1591 (N=N). ^{1}H NMR (400 MHz, Chloroform-*d*) δ 8.53 (s, 2H, C(3')*H*, C(5')*H*), 6.51 (d, *J* = 2.3 Hz, 1H, C(6)*H*), 6.18 (d, *J* = 2.3 Hz, 1H, C(2)*H*), 4.32 (br s, 2H, N*H₂*), 3.88 (s, 3H, OCH_3). ^{13}C NMR (101 MHz, Chloroform-*d*) δ 155.2 (*C*(3)), 155.0 (*C*(1')), 151.5 (*C*(1)), 148.8 (*C*(3'), *C*(4')), 138.5 (*C*(4)), 131.4 (*C*(5)), 123.8 (*C*(2'), *C*(5')), 108.9 (*C*(6)), 96.7 (*C*(2)), 56.6 (OCH_3). HPLC-PDA-MS: method A was used; RT: 3.59 min, *m/z* (ESI$^+$) 331 (MH$^+$), λ_{max} = 382 nm (*trans* isomer); purity 89 % (254 nm). HRMS (ESI$^+$) found 330.9930, $C_{12}H_{10}N_4OCl_3$ (MH$^+$) requires 330.920.

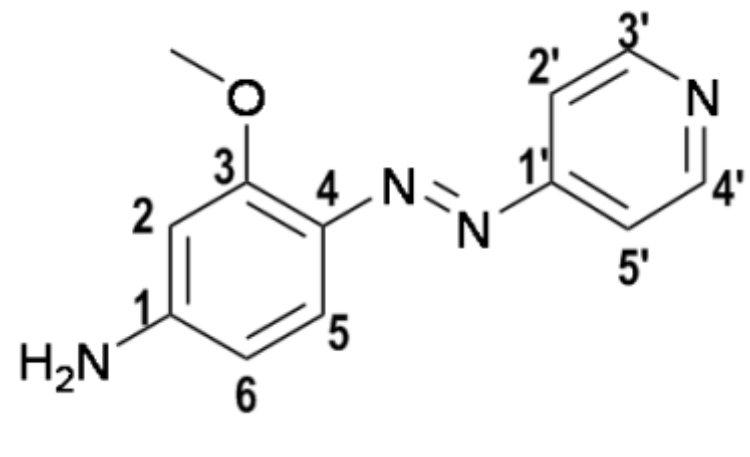

(*E*)-3-Methoxy-4-(pyridin-4-yldiazenyl)aniline 12. To a stirred solution of sodium nitrite (176 mg, 2.55 mmol) in water (4.0 mL) at 0 ºC, an ice-cold solution of pyridin-4-amine (200 mg, 2.13 mmol) in H_3PO_{4conc}/HNO_{3conc} 2:1 (20.0 mL) was added slowly keeping the temperature between 0-5 ºC. The solution was stirred at 0-5ºC for 45 min. Then, it was added dropwise into a solution of 3-methoxyaniline (0.28 mL, 2.55 mmol) in water/H_3PO_{4conc} 2:1 (20.0 mL) keeping temperature of the reaction at 0-5 ºC. It was left 1h from 0-5ºC to room temperature. Afterwards, the reaction mixture was partitioned with EtOAc and saturated aqueous Na_2CO_3. The aqueous phase was backextracted with EtOAc and the combined organic layers were washed with water, saturated aqueous $NaHCO_3$, water (10x) and then brine. The organic phase was dried over anhydrous $MgSO_4$, filtered, and concentrated under reduced pressure. The crude was purified by *flash* column chromatography

(DCM/MeOH 95:5) and then (Hexanes/EtOAc 20:80) to give the title compound as a red solid (222 mg, 0.845 mmol, 54 %). Mp: 164 – 165 ºC. ν_{max}/cm^{-1} 3430 (N-H), 3226 (N-H), 2980 (C-H), 2919 (C-H), 1594 (N=N). ^{1}H NMR (400 MHz, Chloroform-*d*) δ 8.76 - 8.57 (m, 2H, C(3')*H*, C(4')*H*), 7.74 (d, *J* = 9.4 Hz, 1H, C(5)*H*), 7.68 - 7.55 (m, 2H, C(2')*H*, C(5')*H*), 6.37 - 6.23 (m, 2H, C(2)*H*, C(6)*H*), 4.34 (br s, 2H, N*H₂*), 3.97 (s, 3H, OCH_3). ^{13}C NMR (101 MHz, Chloroform-*d*) δ 160.5 (*C*(3)), 158.5 (*C*(1')), 153.3 (*C*(1)), 151.0 (*C*(3'), *C*(4')), 135.2 (*C*(4)), 119.1 (*C*(5)), 116.3 (*C*(2'), *C*(5')), 107.8 (*C*(6)), 97.5 (*C*(2)), 56.3 (OCH_3). HPLC-PDA-MS: method A was used; RT: 2.76 min, *m/z* (ESI$^+$) 229 (MH$^+$), λ_{max} = 382, 504 nm (*trans* isomer); purity 90 % (254 nm). HRMS (ESI$^+$) found 229.1083, $C_{12}H_{13}N_4O$ (MH$^+$) requires 229.1089.

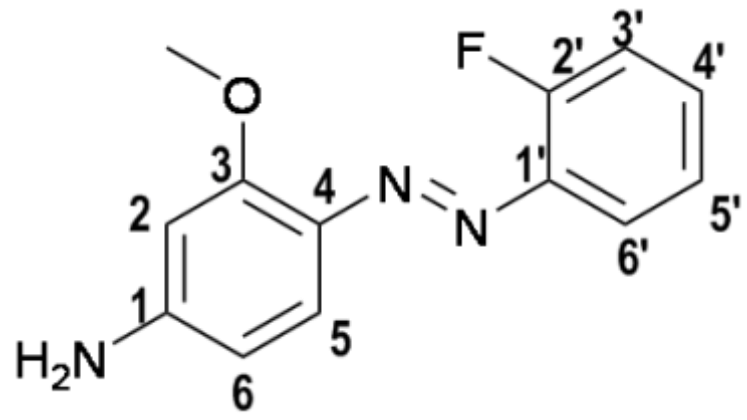

(*E*)-4-((2-Fluorophenyl)diazenyl)-3-methoxyaniline 13. To a stirred solution of 2-fluoroaniline (0.43 mL, 4.5 mmol) in water (7.5 mL) in an ice bath, HCl_{conc} (1.20 mL, 14.6 mmol) was added dropwise while keeping the temperature between 0-5 ºC. Then, an aqueous solution of sodium nitrite (310 mg, 4.50 mmol) in water (1.8 mL) was added dropwise to the mixture to generate the azonium intermediate and it was left stirring for 10 min. In a separate reaction vessel, a solution of 3-methoxyaniline (0.61 mL, 5.52 mmol) and sodium acetate (1.114 g, 14.0 mmol) in water (12.0 mL) was prepared and cooled in an ice bath. The diazonium solution was added to the 3-methoxyaniline solution in dropwise fashion while keeping the temperature between 0-5 ºC and then the resulting mixture was left stirring for another 30 min. Afterwards, water was added dropwise to induce the precipitacion of a red solid which was isolated by filtration and washed with water. The red solid was disolved using EtOAc and then dried over anhydrous $MgSO_4$, filtered, and concentrated under reduced pressure. The crude was purified by *flash* column chromatography (Hexanes/EtOAc 60:40 to EtOAc) to give the title compound as an orange solid (731 mg, 2.98 mmol, 66 %). Mp: 203 - 204 ºC. ν_{max}/cm^{-1} 3369 (N-H), 3211 (C-H), 2966 (C-H), 1621 (N-H),1584 (N=N). ^{1}H NMR (400 MHz, Chloroform-*d*) δ 7.80 - 7.62 (m, 2H, C(5)*H*, C(6')*H*), 7.35 - 7.29 (m, 1H, C(4')*H*), 7.23 - 7.08 (m, 2H, C(3')*H*, C(5')*H*), 6.30 - 6.25 (m, 2H, C(2)*H*, C(6)*H*), 4.15 (s, 2H, NH_2), 3.98 (s, 3H, OCH_3). ^{13}C NMR (101 MHz, Chloroform-*d*) δ 159.9 (d, *J* = 131 Hz, *C*(2')), 159.6 (*C*(3)), 151.9 (*C*(1)), 140.5 (*C*(1')), 135.6 (*C*(4)), 130.5 (*C*(4')), 124.2 (*C*(5')), 118.9 (*C*(5)), 118.3 (*C*(6')) , 116.5 (d, *J* = 18 Hz, *C*(3')), 107.5 (*C*(6)), 97.7 (*C*(2)), 56.2 (OCH_3). HPLC-PDA-MS: method A was used; RT: 3.28 min, *m/z* (ESI$^+$) 246 (MH$^+$), λ_{max} = 452 nm (*trans* isomer); purity 95 % (254 nm). HRMS (ESI$^+$) found 246.1045, $C_{13}H_{13}N_3OF$ (MH$^+$) requires 246.1043.

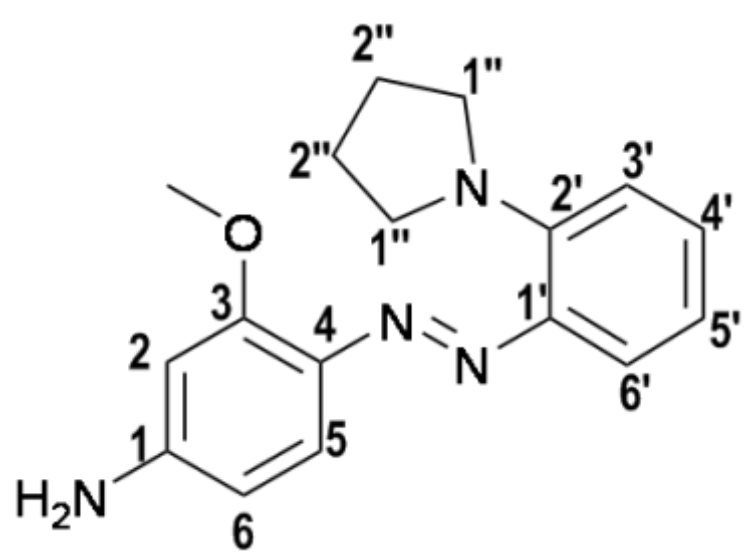

(E)-3-methoxy-4-((2-(pyrrolidin-1-yl)phenyl)diazenyl)aniline 14. To a round-bottomed flask containing azocompound **13** (80.0 mg, 0.326 mmol), pyrrolidine (6.0 mL, 73.1 mmol) was added, and the resulting reaction mixture was left stirring for 48 h at 50 ºC. Afterwards, the reaction mixture was partitioned with EtOAc and water. The aqueous phase was washed with EtOAc (x3) and the combined organic layers were washed brine. The organic phase was dried over anhydrous $MgSO_4$, filtered, and

concentrated under reduced pressure. The crude was purified by *flash* column chromatography (DCM) to give the title compound as a dark red solid (52.6 mg, 0.177 mmol, 54 %). Mp: 95 - 97 ºC. ν_{max}/cm^{-1} 3345 (N-H), 2930 (C-H), 2830 (C-H), 1595 (N=N). ^{1}H NMR (400 MHz, Chloroform-*d*) δ 7.66 (dd, *J* = 8.1, 1.7 Hz, 1H, C(6')*H*), 7.51 (d, *J* = 8.6 Hz, 1H, C(5)*H*), 7.19 (ddd, *J* = 8.5, 7.0, 1.7 Hz, 1H, C(4')*H*), 6.80 (dd, *J* = 8.5, 1.3 Hz, 1H, C(3')*H*), 6.68 (ddd, *J* = 8.1, 7.0, 1.3 Hz, 1H, C(5')*H*), 6.32 (d, *J* = 2.3 Hz, 1H, C(2)*H*), 6.27 (dd, *J* = 8.6, 2.3 Hz, 1H, C(6)*H*), 3.97 (s, 1H), 3.62 - 3.57 (m, 4H, C(1'')H_2), 2.02 - 1.94 (m, 4H, C(2'')H_2). ^{13}C NMR (101 MHz, Chloroform-*d*) δ 158.6 (*C*(3)), 150.1 (*C*(1)), 146.5 (*C*(2')), 141.6 (*C*(1')), 136.0 (*C*(4)), 130.6 (*C*(4')), 118.5 (*C*(5)), 117.6 (*C*(6')), 116.9 (*C*(5')), 115.4 (*C*(3')), 107.5 (*C*(6)), 98.5 (*C*(2)), 56.3 (OCH_3), 52.5 (*C*(1'')H_2), 26.0 (*C*(2'')H_2). HPLC-PDA-MS: method A was used; RT: 3.12 min, *m/z* (ESI$^+$) 297 (MH$^+$), λ_{max} = 460 nm (*trans* isomer); purity could not be determined because of its stability under acidic conditions. HRMS (ESI$^+$) found 297.1711, $C_{17}H_{21}N_4O$ (MH$^+$) requires 297.1715.

Synthesis of Final Compounds

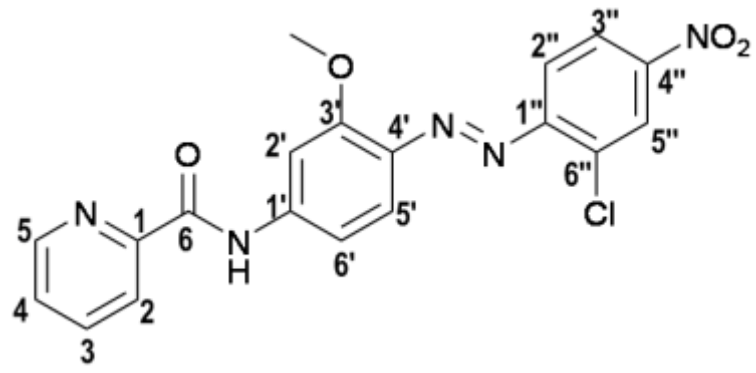

(*E*)-*N*-(4-((2-Chloro-4-nitrophenyl)diazenyl)-3-methoxyphenyl)picolinamide prosthe6-2. To a stirred solution of azocompound **2** (18.0 mg, 0.059 mmol), picolinic acid (21.6 mg, 0.178 mmol) and HATU (133 mg, 0.352 mmol) in DMF (3.0 mL), TEA (98 µL, 0.704 mmol) was added and then left stirring 48 h at 40 ºC. Afterwards, the reaction mixture was partitioned with EtOAc and saturated aqueous Na_2CO_3. The aqueous phase was backextracted with EtOAc and the combined organic layers were washed with water, saturated aqueous $NaHCO_3$, water (10x) and then brine. The organic phase was dried over anhydrous $MgSO_4$, filtered, and concentrated under reduced pressure. The crude was purified by *flash* column chromatography (DCM) to give the title compound as a red solid (13.6 mg, 0.033 mmol, 56 %). Mp: 234 - 235 ºC. ν_{max}/cm^{-1} 3316 (N-H), 2937 (C-H), 2845 (C-H), 1674 (C=O), 1584 (N=N). ^{1}H NMR (400 MHz, Chloroform-*d*) δ 10.34 (br s, 1H, C(6)ON*H*), 8.66 (ddd, *J* = 4.8, 1.7, 0.9 Hz, 1H, C(5)*H*), 8.41 (d, *J* = 2.4 Hz, 1H, C(5'')*H*), 8.32 (dt, *J* = 7.8, 1.2 Hz, 1H, C(2)*H*), 8.18 (dd, *J* = 8.9, 2.4 Hz, 1H, C(3'')*H*), 8.15 (d, *J* = 2.1 Hz, 1H, C(2')*H*), 7.96 (td, *J* = 7.6, 1.7 Hz, 1H, C(3)*H*), 7.90 (d, *J* = 8.8 Hz, 1H, C(5')*H*), 7.78 (d, *J* = 8.8 Hz, 1H, C(2'')*H*), 7.55 (ddd, *J* = 7.6, 4.8, 1.2 Hz, 1H, C(4)*H*), 7.10 (dd, *J* = 8.8, 2.1 Hz, 1H, C(6')*H*), 4.13 (s, 3H, OCH_3). ^{13}C NMR (101 MHz, Chloroform-*d*) δ 162.6 (*C*(6)), 159.9 (*C*(3')), 153.3 (*C*(4'')), 149.2 (*C*(1)), 148.3 (*C*(1'')), 148.2 (*C*(5)), 144.2 (*C*(4')), 139.0 (*C*(1')), 138.1 (*C*(3)), 134.8 (*C*(6'')), 127.2 (*C*(4)), 126.2 (*C*(5'')), 122.9 (*C*(3'')), 122.7 (*C*(2)), 119.0 (*C*(2'')), 118.5 (*C*(5')), 112.1 (*C*(6')), 103.4 (*C*(2')), 56.6 (OCH_3). HPLC-PDA-MS: method A was used; RT: 4.53 min, *m/z* (ESI$^+$) 412 (MH$^+$), λ_{max} = 410 nm (*trans* isomer); purity 98 % (254 nm). HRMS (ESI$^+$) found 412.0817, $C_{19}H_{15}N_5O_4Cl$ (MH$^+$) requires 412.0813.

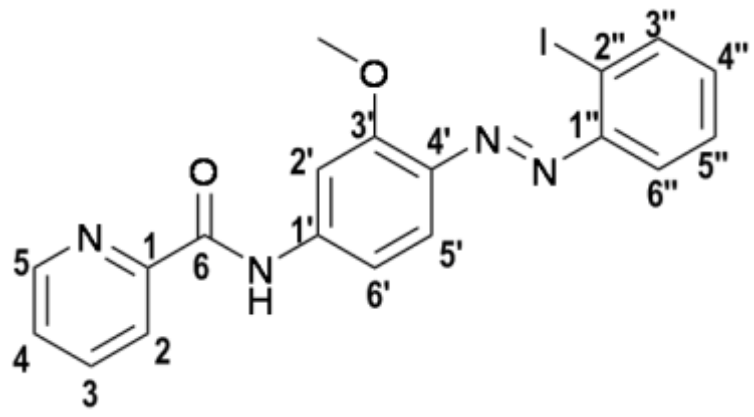

(*E*)-*N*-(4-((2-Iodophenyl)diazenyl)-3-methoxyphenyl)picolinamide prosthe6-3. To a stirred solution of azocompound **3** (30.0 mg, 0.085 mmol), picolinic acid (15.7 mg, 0.127 mmol) and HATU (97.0 mg, 0.255 mmol) in DMF (3.0 mL), TEA (71 µL, 0.510 mmol) was added and then left stirring 18 h at 40 ºC. Afterwards, the reaction mixture was partitioned with EtOAc and saturated aqueous Na_2CO_3. The aqueous phase was backextracted with EtOAc and the combined organic layers were washed with water, saturated aqueous $NaHCO_3$, water (10x) and then brine. The organic phase was dried over

anhydrous $MgSO_4$, filtered, and concentrated under reduced pressure. The crude was purified by *flash* column chromatography (DCM) to give the title compound as an orange solid (17.8 mg, 0.039 mmol, 46 %). Mp: 192 - 193 ºC. ν_{max}/cm^{-1} 3342 (N-H), 3006 (C-H), 2920 (C-H), 1683 (C=O),1585 (N=N). ^1H NMR (400 MHz, Chloroform-*d*) δ 10.27 (br s, 1H, C(6)ON*H*), 8.65 (ddd, *J* = 4.8, 1.7, 0.9 Hz, 1H, C(5)*H*), 8.31 (dt, *J* = 7.9, 1.1 Hz, 1H, C(2)*H*), 8.10 (d, *J* = 2.1 Hz, 1H, C(2')*H*), 7.99 (dd, *J* = 7.9, 1.3 Hz, 1H, C(3'')*H*), 7.94 (td, *J* = 7.9, 1.7 Hz, 1H, C(3)*H*), 7.90 (d, *J* = 8.7 Hz, 1H, C(5')*H*), 7.63 (dd, *J* = 8.0, 1.6 Hz, 1H, C(6'')*H*), 7.52 (ddd, *J* = 7.9, 4.8, 1.1 Hz, 1H, C(4)*H*), 7.40 (ddd, *J* = 8.0, 7.2, 1.3 Hz, 1H, C(5'')*H*), 7.16 - 7.07 (m, 2H, C(6')*H*, C(4'')*H*), 4.11 (s, 3H, OC*H₃*). ^{13}C NMR (101 MHz, Chloroform-*d*) δ 162.4 (*C*(6)), 158.8 (*C*(3')), 152.1 (*C*(1'')), 149.4 (*C*(1)), 148.2 (*C*(5)), 142.6 (*C*(4')), 139.7 (*C*(3'')), 138.4 (*C*(1')), 138.0 (*C*(3)), 131.7 (*C*(4'')), 129.1 (*C*(5'')), 127.0 (*C*(4)), 122.6 (*C*(2)), 118.6 (*C*(5')), 118.0 (*C*(6'')), 111.9 (*C*(6')), 103.6 (*C*(2')), 102.0 (*C*(2'')), 56.5 (O*C*H_3). HPLC-PDA-MS: method A was used; RT: 3.97 min, *m/z* (ESI$^+$) 385 (MH$^+$), λ_{max} = 446 nm (*cis* isomer); RT: 4.60 min, *m/z* (ESI$^+$) 385 (MH$^+$), λ_{max} = 388 nm (*trans* isomer); purity >99 % (254 nm). HRMS (ESI$^+$) found 385.0854, $C_{19}H_{15}N_4O_2FCl$ (MH$^+$) requires 385.0868.

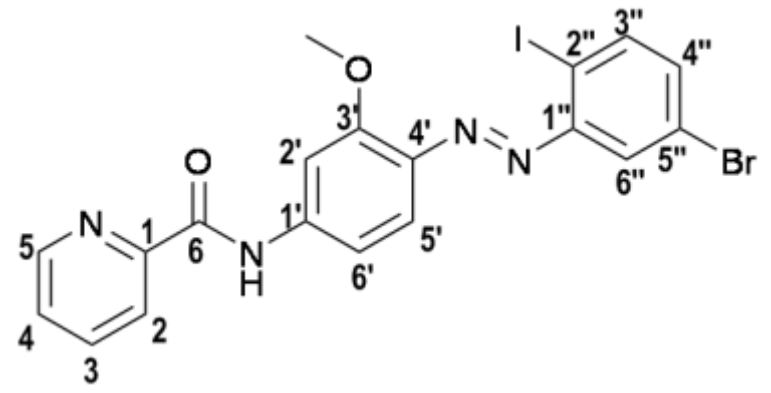

(*E*)-*N*-(4-((5-Bromo-2-iodophenyl)diazenyl)-3-methoxyphenyl)picolinamide prosthe6-4. To a stirred solution of azocompound **4** (30.0 mg, 0.069 mmol), picolinic acid (25.6 mg, 0.208 mmol) and HATU (158 mg, 0.416 mmol) in DMF (3.0 mL), TEA (116 µL, 0.834 mmol) was added and then left stirring 48 h at 40 ºC. Afterwards, the reaction mixture was partitioned with EtOAc and saturated aqueous Na_2CO_3. The aqueous phase was backextracted with EtOAc and the combined organic layers were washed with water, saturated aqueous $NaHCO_3$, water (10x) and then brine. The organic phase was dried over anhydrous $MgSO_4$, filtered, and concentrated under reduced pressure. The crude was purified by *flash* column chromatography (Hexanes/EtOAc 50:50) to give the title compound as an orange solid (30.6 mg, 0.057 mmol, 82 %). Mp: 198 - 199 ºC. ν_{max}/cm^{-1} 3340 (N-H), 3060 (C-H), 2921 (C-H), 1688 (C=O), 1581 (N=N). ^1H NMR (400 MHz, Chloroform-*d*) δ 10.28 (br s, 1H, C(6)ON*H*), 8.65 (ddd, *J* = 4.8, 1.7, 0.9 Hz, 1H, C(5)*H*), 8.31 (dt, *J* = 7.7, 1.1 Hz, 1H, C(2)*H*), 8.14 (dd, *J* = 1.6, 0.7 Hz, 1H, C(6'')*H*), 8.10 (d, *J* = 2.1 Hz, 1H, C(2')*H*), 7.95 (td, *J* = 7.7, 1.7 Hz, 1H, C(3)*H*), 7.89 (d, *J* = 8.8 Hz, 1H, C(5')*H*), 7.58 - 7.46 (m, 3H, C(4)*H*, C(3'')*H*, C(4'')*H*), 7.10 (dd, *J* = 8.8, 2.1 Hz, 1H, C(6')*H*), 4.11 (s, 3H, OC*H₃*). ^{13}C NMR (101 MHz, Chloroform-*d*) δ 162.4 (*C*(6)), 158.9 (*C*(3')), 151.0 (*C*(1'')), 149.4 (*C*(1)), 148.2 (*C*(5)), 143.0 (*C*(4')), 141.7 (*C*(6'')), 138.3 (*C*(1')), 138.1 (*C*(3)), 132.2 (*C*(4'')), 127.0 (*C*(4)), 125.0 (*C*(5'')), 122.6 (*C*(2)), 118.7 (*C*(5')), 118.7 (*C*(3'')), 112.0 (*C*(6')), 103.5 (*C*(2')), 102.7 (*C*(2'')), 56.5 (O*C*H_3). HPLC-PDA-MS: method A was used; RT: 4.34 min, *m/z* (ESI$^+$) 537 (MH$^+$), λ_{max} = 446 nm (*cis* isomer); RT: 5.26 min, *m/z* (ESI$^+$) 537 (MH$^+$), λ_{max} = 398 nm (*trans* isomer); purity 98 % (254 nm). HRMS (ESI$^+$) found 536.9409, $C_{19}H_{15}N_4O_2BrI$ (MH$^+$) requires 536.9423.

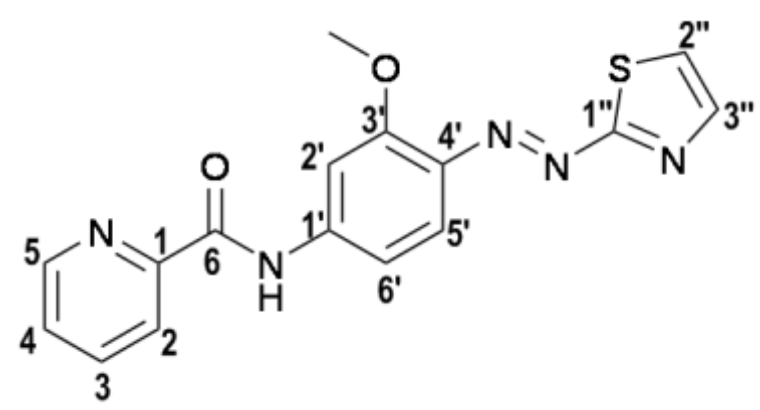

(*E*)-*N*-(3-Methoxy-4-(thiazol-2-yldiazenyl)phenyl)picolinamide prosthe6-5. To a stirred solution of azocompound **1** (20.0 mg, 0.085 mmol), picolinic acid (15.8 mg, 0.128 mmol) and HATU (97.0 mg, 0.256 mmol) in DMF (2.0 mL), TEA (71 µL, 0.512 mmol) was added and then left stirring 18 h at 40 ºC. Afterwards, the reaction mixture was partitioned with EtOAc and saturated aqueous Na_2CO_3. The aqueous phase was backextracted with EtOAc and the combined organic layers were washed with

water, saturated aqueous $NaHCO_3$, water (10x) and then brine. The organic phase was dried over anhydrous $MgSO_4$, filtered and concentrated under reduced pressure. The crude was purified by reverse phase automated column chromatography (Water/ACN 95:5 to ACN + 0.05 % HCOOH) to give the title compound as a red solid (10.6 mg, 0.031 mmol, 37 %). ν_{max}/cm^{-1} 3319 (N-H), 2922 (C-H), 2851 (C-H), 1692 (C=O), 1581 (N=N). ^{1}H NMR (400 MHz, Chloroform-*d*) δ 10.37 (br s, 1H, C(6)ON*H*), 8.65 (ddd, *J* = 4.8, 1.7, 0.9 Hz, 1H, C(5)*H*), 8.32 (dt, *J* = 7.9, 1.2 Hz, 1H, C(2)*H*), 8.11 (d, *J* = 2.1 Hz, 1H, C(2')*H*), 8.05 - 7.91 (m, 3H, C(3)*H*, C(3'')*H*, C(5')*H*), 7.55 (ddd, *J* = 7.9, 4.8, 1.2 Hz, 1H, C(4)*H*), 7.37 (d, *J* = 3.3 Hz, 1H, C(2'')*H*), 7.11 (dd, *J* = 8.9, 2.1 Hz, 1H, C(6')*H*), 4.11 (s, 3H, OC*H₃*). ^{13}C NMR (101 MHz, Chloroform-*d*) δ 178.2 (*C*(1'')), 162.4 (*C*(6)), 159.6 (*C*(3')), 149.2 (*C*(1)), 148.1 (*C*(5)), 144.3 (*C*(4')), 143.5 (*C*(3'')), 138.3 (*C*(3)), 137.8 (*C*(1')), 127.2 (*C*(4)), 122.8 (*C*(2)), 120.7 (*C*(5')), 119.0 (*C*(2'')), 112.2 (*C*(6')), 103.5 (*C*(2')), 56.7 (O*C*H_3). HPLC-PDA-MS: method A was used; RT: 3.74 min, *m/z* (ESI$^+$) 340 (MH$^+$), λ_{max} = 430 nm (*trans* isomer); purity 91 % (254 nm). HRMS (ESI$^+$) found 340.0858, $C_{16}H_{14}N_5O_2S$ (MH$^+$) requires 340.0868.

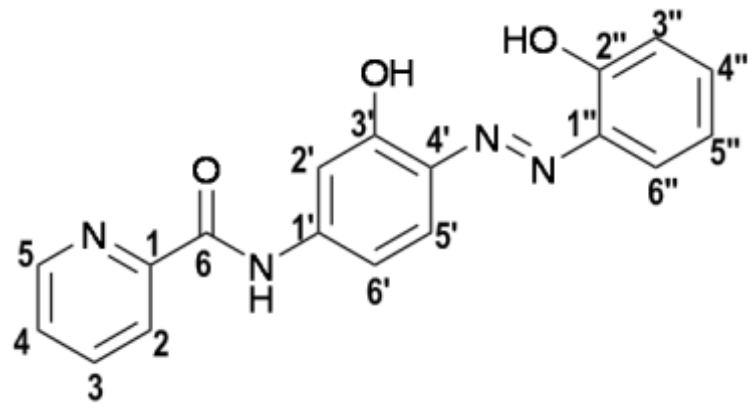

(*E*)-*N*-(3-Hydroxy-4-((2-hydroxyphenyl)diazenyl)phenyl)picolinamide prosthe6-6. To a stirred solution of azocompound **8** (85.0 mg, 0.235 mmol) in dry DCM (2.4 mL) at -78 ºC under inert atmosphere, a solution 1 M of BBr_3 in DCM (1.4 mL, 1.41 mmol) was added in a dropwise fashion. The temperature was kept for 1 h at -78 ºC and then it was allowed to reach room temperature. After 18 h, the reaction mixture was cooled down to -78ºC and the reaction was quenched with the dropwise addition of MeOH. Afterwards, the reaction mixture was partitioned with more DCM and water. The aqueous phase was backextracted with DCM and the combined organic layers were washed with water, then brine. The organic phase was dried over anhydrous $MgSO_4$, filtered, and concentrated under reduced pressure. The crude was purified by reverse phase automated column chromatography (Water/ACN 95:5 to ACN + 0.05 % HCOOH) to give the title compound as a dark red solid (19.5 mg, 0.058 mmol, 25 %). ν_{max}/cm^{-1} 3462 (O-H), 3316 (N-H), 2919 (C-H), 2850 (C-H), 1689 (C=O), 1575 (N=N). ^{1}H NMR (400 MHz, Chloroform-*d*) δ 12.63 (br s, 1H, C(3')-O*H*), 12.23 (br s, 1H, C(2'')-O*H*), 10.26 (br s, 1H, C(6)ON*H*), 8.68 - 8.61 (m, 1H, C(5)*H*), 8.32 (d, *J* = 7.8 Hz, 1H, C(2)*H*), 7.95 (td, *J* = 7.8, 1.7 Hz, 1H, C(3)*H*), 7.71 (d, *J* = 8.7 Hz, 1H, C(5')*H*), 7.68 (dd, *J* = 7.9, 1.7 Hz, 1H, C(6'')*H*), 7.59 (d, *J* = 2.2 Hz, 1H, C(2')*H*), 7.53 (ddd, *J* = 7.8, 4.7, 1.2 Hz, 1H, C(4)*H*), 7.48 (dd, *J* = 8.7, 2.2 Hz, 1H, C(6')*H*), 7.33 (td, *J* = 7.9, 1.7 Hz, 1H, C(4'')*H*), 7.09 - 7.00 (m, 2H, C(3'')*H*, C(5'')*H*). ^{13}C NMR (101 MHz, Chloroform-*d*) δ 162.4 (*C*(6)), 154.8 (*C*(3')), 153.0 (*C*(2'')), 149.3 (*C*(1)), 148.1 (*C*(5)), 142.1 (*C*(4')), 138.2 (*C*(3)), 135.2 (*C*(1'')), 132.8 (*C*(4'')), 132.4 (*C*(1')), 132.3 (*C*(5')), 130.9 (*C*(6'')), 127.1 (*C*(4)), 122.9 (*C*(2)), 120.4 (*C*(5'')), 118.7 (*C*(3'')), 112.0 (*C*(6')), 108.4 (*C*(2')). HPLC-PDA-MS: method A was used; RT: 4.35 min, *m/z* (ESI$^+$) 335 (MH$^+$), λ_{max} = 418 nm (*trans* isomer); purity 95 % (254 nm). HRMS (ESI$^+$) found 335.1147, $C_{18}H_{15}N_4O_3$ (MH$^+$) requires 335.1144.

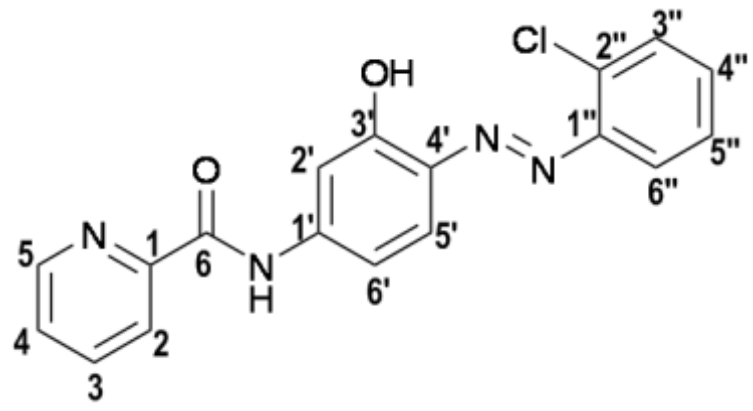

(*E*)-*N*-(4-((2-Chlorophenyl)diazenyl)-3-hydroxyphenyl)picolinamide prosthe6-7. To a stirred solution of **prosthe6-1** (437 mg, 0.235 mmol) in dry DCM (6.5 mL) at -78 ºC under inert atmosphere, a solution 1 M of BBr_3 in DCM (3.6 mL, 3.60 mmol) was added in a dropwise fashion. The temperature was kept for 1 h at -78 ºC and then it was allowed to reach room temperature. After 18 h, the reaction mixture

was cooled down to -78ºC and the reaction was quenched with the dropwise addition of MeOH. Afterwards, the reaction mixture was partitioned with more DCM and water. The aqueous phase was backextracted with DCM and the combined organic layers were washed with water, then brine. The organic phase was dried over anhydrous $MgSO_4$, filtered and concentrated under reduced pressure. The crude was purified by reverse phase automated column chromatography (Water/ACN 95:5 to ACN + 0.05 % HCOOH) to give the title compound as an orange solid (16.1 mg, 0.046 mmol, 4 %). ν_{max}/cm^{-1} 3313 (N-H), 3069 (O-H), 2917 (C-H), 2849 (C-H), 1693 (C=O), 1569 (N=N). ^{1}H NMR (400 MHz, Chloroform-*d*) δ 13.44 (s, 1H, C(3')-O*H*), 10.27 (br s, 1H, C(6)ON*H*), 8.65 (dt, *J* = 4.8, 1.3 Hz, 1H, C(5)*H*), 8.32 (dt, *J* = 7.9, 1.1 Hz, 1H, C(2)*H*), 7.99 - 7.89 (m, 3H, C(3)*H*, C(5')*H*, C(6'')*H*), 7.61 - 7.48 (m, 4H, C(4)*H*, C(3'')*H*, C(2')*H*, C(6')*H*), 7.45 - 7.33 (m, 2H, C(4'')*H*, C(5'')*H*). ^{13}C NMR (101 MHz, Chloroform-*d*) δ 162.4 (*C*(6)), 154.8 (*C*(3')), 149.4 (*C*(1)), 148.2 (*C*(5)), 146.6 (*C*(1'')), 142.8 (*C*(4')), 138.0 (*C*(3)), 135.4 (*C*(1')), 135.0 (*C*(6'')), 133.8 (*C*(2'')), 131.5 (*C*(4'')), 130.6 (*C*(3'')), 127.7 (*C*(5'')), 127.0 (*C*(4)), 122.8 (*C*(2)), 117.4 (*C*(5')), 111.8 (*C*6')), 108.0 (*C*(2')). HPLC-PDA-MS: method A was used; RT: 4.95 min, *m/z* (ESI^+) 353 (MH^+), λ_{max} = 400 nm (*trans* isomer); purity 98 % (254 nm). HRMS (ESI^+) found 353.0800, $C_{18}H_{14}N_4O_2Cl$ (MH^+) requires 353.0805.

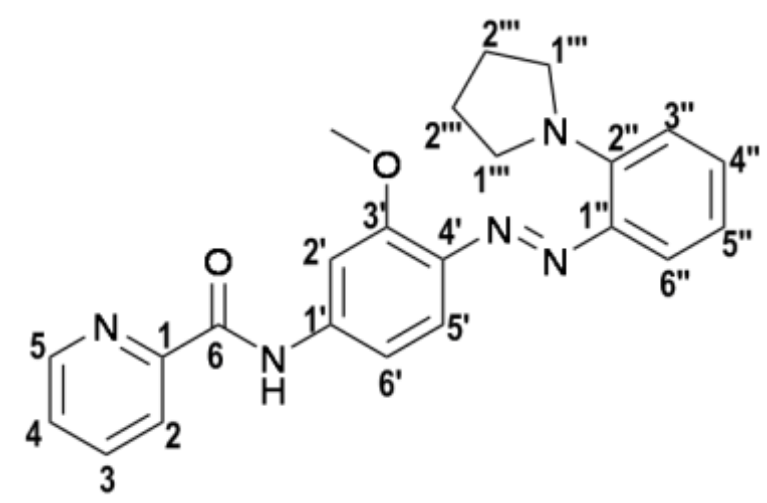

(*E*)-*N*-(3-Methoxy-4-((2-(pyrrolidin-1-yl)phenyl)diazenyl)phenyl)picolinamide prosthe6-8. To a stirred solution of azocompound **14** (34.0 mg, 0.115 mmol) in dry DMF (2.0 mL) kept at 0 ºC, DIPEA (60 µL, 0.344 mmol) was added and then picolinoyl chloride hydrochloride (24.5 mg, 0.138 mmol). The reaction was allowed to warm up to room temperature and then left stirring for 18 h. Afterwards, the reaction mixture was partitioned with EtOAc and saturated aqueous Na_2CO_3. The aqueous phase was backextracted with EtOAc and the combined organic layers were washed with water, saturated aqueous $NaHCO_3$, water (x3) and then brine. The organic phase was dried over anhydrous $MgSO_4$, filtered, and concentrated under reduced pressure. The crude was purified by *flash* column chromatography (Hexanes/EtOAc 80:20) to give the title compound as a dark red solid (43.4 mg, 0.108 mmol, 94 %). ν_{max}/cm^{-1} 3328 (N-H), 2925 (C-H), 2850 (C-H), 1678 (C=O), 1598 (N=N). ^{1}H NMR (400 MHz, Chloroform-*d*) δ 10.19 (br s, 1H, C(6)ON*H*), 8.64 (ddd, *J* = 4.8, 1.7, 0.9 Hz, 1H, C(5)*H*), 8.32 (dt, *J* = 7.8, 1.1 Hz, 1H, C(2)*H*), 7.99 - 7.90 (m, 2H, C(3)*H*, C(2')*H*), 7.75 (dd, *J* = 8.2, 1.7 Hz, 1H, C(6'')*H*), 7.60 (d, *J* = 8.6 Hz, 1H, C(5')*H*), 7.51 (ddd, *J* = 7.8, 4.8, 1.1 Hz, 1H, C(4)*H*), 7.26 - 7.21 (m, 1H, C(4'')*H*), 7.18 (dd, *J* = 8.6, 2.2 Hz, 1H, C(6')*H*), 6.82 (dd, *J* = 8.5, 1.3 Hz, 1H, C(3'')*H*), 6.68 (ddd, *J* = 8.2, 6.9, 1.3 Hz, 1H, C(5'')*H*), 4.10 (s, 3H, OCH_3), 3.71 - 3.62 (m, 4H, C(1''')H_2), 2.09 - 1.94 (m, 4H, C(2''')H_2). ^{13}C NMR (101 MHz, Chloroform-*d*) δ 162.2 (*C*(6)), 157.5 (*C*(3')), 149.7 (*C*(1)), 148.2 (*C*(5)), 147.1 (*C*(2'')), 141.3 (*C*(1'')), 140.4 (*C*(4')), 139.6 (*C*(1')), 138.0 (*C*(3)), 131.7 (*C*(4'')), 126.8 (*C*(4)), 122.6 (*C*(2)), 117.6 (*C*(6'')), 117.6 (*C*(5')), 116.6 (*C*(5'')), 115.7 (*C*(3'')), 111.8 (*C*(6')), 103.9 (*C*(2')), 56.5 (O*C*H_3), 52.6 (*C*(1''')H_2), 26.1 (*C*(2''')H_2). HPLC-PDA-MS: method A was used; RT: 3.16 min, *m/z* (ESI^+) 402 (MH^+), λ_{max} = 416, 534 nm (*trans* isomer); purity purity could not be determined because of its stability under acidic conditions. HRMS (ESI^+) found 402.1938, $C_{23}H_{24}N_5O_2$ (MH^+) requires 402.1930.

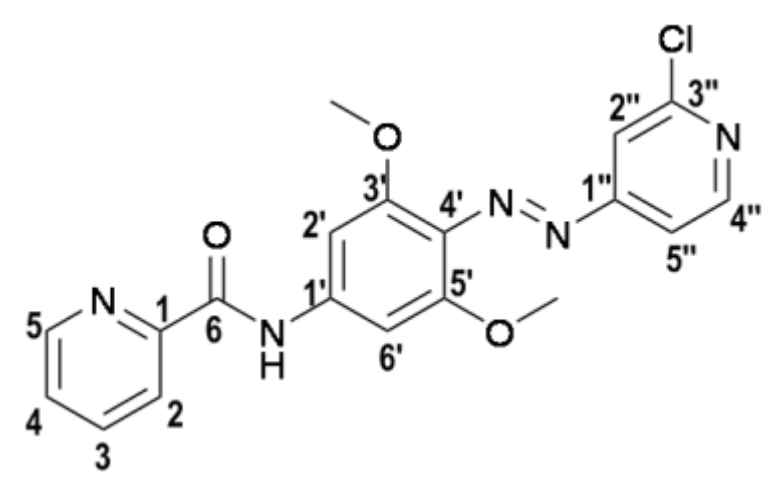

(*E*)-*N*-(4-((2-Chloropyridin-4-yl)diazenyl)-3,5-dimethoxyphenyl)picolinamide prosthe6-9. To a stirred solution of azocompound **9** (15.0 mg, 0.051 mmol), picolinic acid (9.5 mg, 0.077 mmol) and HATU (58.5 mg, 0.154 mmol) in DMF (2.0 mL), TEA (43 µL, 0.307 mmol) was added and then left stirring 72 h at room temperature. Afterwards, the reaction mixture was partitioned with EtOAc and saturated aqueous Na_2CO_3. The aqueous phase was backextracted with EtOAc and the combined organic layers were washed with water, saturated aqueous $NaHCO_3$, water (10x) and then brine. The organic phase was dried over anhydrous $MgSO_4$, filtered and concentrated under reduced pressure. The crude was purified by *flash* column chromatography (Hexanes/EtOAc 50:50) to give the title compound as a red solid (8.3 mg, 0.021 mmol, 41 %). ν_{max}/cm^{-1} 3301 (N-H), 2920 (C-H), 2849 (C-H), 1689 (C=O), 1579 (N=N). ^{1}H NMR (400 MHz, Chloroform-*d*) δ 10.28 (br s, 1H, C(6)ON*H*), 8.66 (ddd, *J* = 4.7, 1.7, 0.9 Hz, 1H, C(5)*H*), 8.50 (dd, *J* = 5.3, 0.7 Hz, 1H, C(4'')*H*), 8.31 (dt, *J* = 7.9, 1.1 Hz, 1H, C(2)*H*), 7.97 (td, *J* = 7.9, 1.7 Hz, 1H, C(3)*H*), 7.65 (dd, *J* = 1.7, 0.7 Hz, 1H, C(2'')*H*), 7.61 (dd, *J* = 5.3, 1.7 Hz, 1H, C(5'')*H*), 7.55 (ddd, *J* = 7.9, 4.7, 1.1 Hz, 1H, C(4)*H*), 7.27 (s, 2H, C(2')*H*, C(6')*H*) 3.99 (s, 6H, 2·OC*H₃*). ^{13}C NMR (101 MHz, Chloroform-*d*) δ 162.6 (*C*(6)), 161.3 (*C*(1'')), 155.8 (*C*(3'), *C*(5')), 152.7 (*C*(3'')), 150.7 (*C*(4'')), 149.3 (*C*(1)), 148.2 (*C*(5)), 142.8 (*C*(1')), 138.2 (*C*(3)), 129.2 (*C*(4')), 127.2 (*C*(4)), 122.7 (*C*(2)), 116.2 (*C*(5'')), 116.0 (*C*(2'')), 96.1 (*C*(2'), *C*(6')), 56.7 (2·O*C*H₃). HPLC-PDA-MS: method A was used; RT: 3.92 min, *m/z* (ESI$^+$) 398 (MH$^+$), λ_{max} = 374 nm (*trans* isomer); purity 96 % (254 nm). HRMS (ESI$^+$) found 398.1002, $C_{19}H_{17}N_5O_3Cl$ (MH$^+$) requires 398.1020.

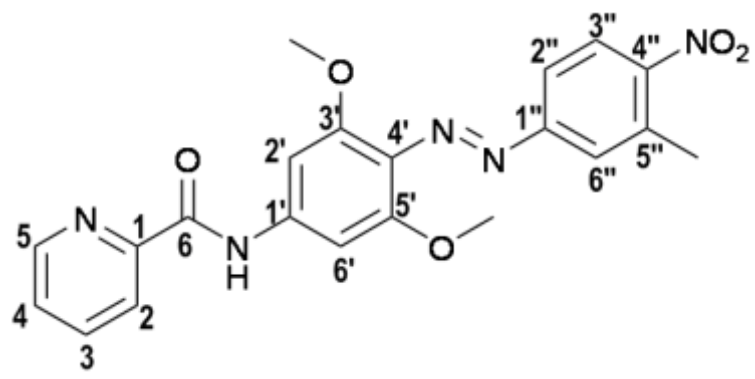

(*E*)-*N*-(3,5-Dimethoxy-4-((3-methyl-4-nitrophenyl)diazenyl)phenyl)picolinamide prosthe6-10. To a stirred solution of azocompound **5** (27.0 mg, 0.085 mmol), picolinic acid (15.8 mg, 0.128 mmol) and HATU (97.0 mg, 0.256 mmol) in DMF (3.0 mL), TEA (71 µL, 0.512 mmol) was added and then left stirring 72 h at room temperature. Afterwards, the reaction mixture was partitioned with EtOAc and saturated aqueous Na_2CO_3. The aqueous phase was backextracted with EtOAc and the combined organic layers were washed with water, saturated aqueous $NaHCO_3$, water (10x) and then brine. The organic phase was dried over anhydrous $MgSO_4$, filtered and concentrated under reduced pressure. The crude was purified by *flash* column chromatography (Hexanes/EtOAc 50:50) to give the title compound as a red solid (20.7 mg, 0.049 mmol, 58 %). ν_{max}/cm^{-1} 3334 (N-H), 2925 (C-H), 2849 (C-H), 1688 (C=O), 1583 (N=N). ^{1}H NMR (400 MHz, Chloroform-*d*) δ 10.25 (br s, 1H, C(6)ON*H*), 8.66 (ddd, *J* = 4.8, 1.7, 0.9 Hz, 1H, C(5)*H*), 8.31 (dt, *J* = 7.7, 1.2 Hz, 1H, C(2)*H*), 8.11 (d, *J* = 9.3 Hz, 1H, C(3'')*H*), 7.96 (td, *J* = 7.7, 1.7 Hz, 1H, C(3)*H*), 7.80 - 7.72 (m, 2H, C(2'')*H*, C(6'')*H*), 7.54 (ddd, *J* = 7.7, 4.8, 1.2 Hz, 1H, C(4)*H*), 7.27 (s, 2H, C(2')*H*, C(6')*H*), 3.98 (s, 6H, 2·OC*H₃*), 2.70 (s, 3H, C*H₃*). ^{13}C NMR (101 MHz, Chloroform-*d*) δ 162.5 (*C*(6)), 156.1 (*C*(1'')), 155.1 (*C*(3'), *C*(5')), 149.4 (*C*(1)), 149.3 (*C*(4'')), 148.2 (*C*(5)), 141.8 (*C*(1')), 138.1 (*C*(3)), 135.2 (*C*(5'')), 129.6 (*C*(4')), 127.1 (*C*(4)), 126.3 (*C*(2'')), 126.0 (*C*(3'')), 122.7 (*C*(2)), 120.6 (*C*(6'')), 96.3 (*C*(2'), *C*(6')), 56.7 (2·O*C*H₃), 21.0 (*C*H₃). HPLC-PDA-MS: method A was used; RT: 4.04 min, *m/z* (ESI$^+$) 422 (MH+), λ_{max} = 446 nm (*cis* isomer); RT: 4.24 min, *m/z* (ESI$^+$) 422 (MH$^+$), λ_{max} = 378 nm (*trans* isomer); purity 98 % (254 nm). HRMS (ESI$^+$) found 422.1463, $C_{21}H_{20}N_5O_5$ (MH$^+$) requires 422.1464

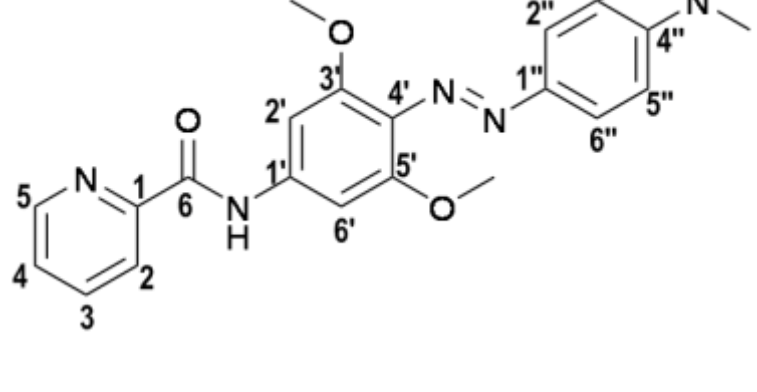

(*E*)-*N*-(4-((4-(Dimethylamino)phenyl)diazenyl)-3,5-dimethoxyphenyl)picolinamide prosthe6-11. To a stirred solution of azocompound **6** (36.0 mg, 0.120 mmol), picolinic acid (22.1 mg, 0.180 mmol) and

HATU (137 mg, 0.360 mmol) in DMF (3.0 mL), TEA (100 µL, 0.719 mmol) was added and then left stirring 72 h at room temperature. Afterwards, the reaction mixture was partitioned with EtOAc and saturated aqueous Na_2CO_3. The aqueous phase was backextracted with EtOAc and the combined organic layers were washed with water, saturated aqueous $NaHCO_3$, water (10x) and then brine. The organic phase was dried over anhydrous $MgSO_4$, filtered and concentrated under reduced pressure. The crude was purified by reverse phase automated column chromatography (Water/ACN 95:5 to ACN + 0.05 % HCOOH) to give the title compound as an orange solid (20.3 mg, 0.050 mmol, 42 %). ν_{max}/cm^{-1} 3318 (N-H), 2919 (C-H), 2848 (C-H), 1682 (C=O), 1597 (N=N). ^{1}H NMR (400 MHz, Chloroform-*d*) δ 10.12 (br s, 1H, C(6)ON*H*), 8.64 (ddd, *J* = 4.8, 1.7, 1.0 Hz, 1H, C(5)*H*), 8.30 (dt, *J* = 7.9, 1.1 Hz, 1H, C(2)*H*), 7.94 (td, *J* = 7.9, 1.7 Hz, 1H, C(3)*H*), 7.87 (d, *J* = 9.1 Hz, 2H, C(2'')*H*, C(6'')*H*), 7.51 (ddd, *J* = 7.9, 4.8, 1.1 Hz, 1H, C(4)*H*), 7.23 (s, 2H, C(2')*H*, C(6')*H*), 6.75 (d, *J* = 9.1 Hz, 2H, C(3'')*H*, C(5'')*H*), 3.89 (s, 6H, 2·OC*H*$_3$), 3.07 (s, 6H, 2·C*H*$_3$). ^{13}C NMR (101 MHz, Chloroform-*d*) δ 162.2 (*C*(6)), 153.3 (*C*(3'), *C*(5')), 152.5 (*C*(4'')), 149.8 (*C*(1)), 148.1 (*C*(5)), 144.8 (*C*(1'')), 138.2 (*C*(1')), 138.0 (*C*(3)), 130.8 (*C*(4')), 126.8 (*C*(4)), 124.7 (*C*(2''), *C*(6'')), 122.5 (*C*(2)), 111.6 (*C*(3''), *C*(5'')), 97.0 (*C*(2'), *C*(6')), 56.7 (2·O*C*H$_3$), 40.5 (2·*C*H$_3$). HPLC-PDA-MS: method A was used; RT: 3.87 min, *m/z* (ESI$^+$) 422 (MH+), λ_{max} = 446 nm (*cis* isomer); RT: 4.24 min, *m/z* (ESI$^+$) 406 (MH$^+$), λ_{max} = 396 nm (*trans* isomer); purity 92 % (254 nm). HRMS (ESI$^+$) found 406.1864, $C_{22}H_{24}N_5O_3$ (MH$^+$) requires 406.1879.

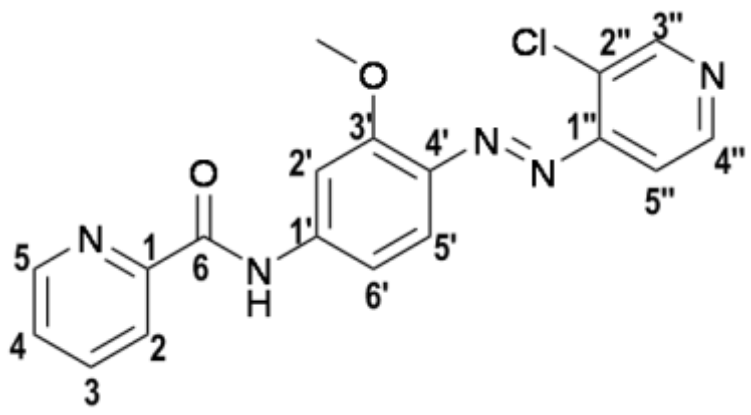

(*E*)-*N*-(4-((3-Chloropyridin-4-yl)diazenyl)-3-methoxyphenyl)picolinamide prosthe6-12. To a stirred solution of azocompound **10** (47.5 mg, 0.181 mmol), picolinic acid (33.4 mg, 0.271 mmol) and HATU (206 mg, 0.542 mmol) in DMF (6.0 mL), TEA (151 µL, 1.08 mmol) was added and then left stirring 18 h at 40 ºC. Afterwards, the reaction mixture was partitioned with EtOAc and saturated aqueous Na_2CO_3. The aqueous phase was backextracted with EtOAc and the combined organic layers were washed with water, saturated aqueous $NaHCO_3$, water (10x) and then brine. The organic phase was dried over anhydrous $MgSO_4$, filtered and concentrated under reduced pressure. The crude was purified by *flash* column chromatography (Hexanes/EtOAc 50:50) to give the title compound as an orange solid (40.2 mg, 0.109 mmol, 60 %). Mp: 213 - 214 ºC. ν_{max}/cm^{-1} 3343 (N-H), 2924 (C-H), 2849 (C-H), 1687 (C=O), 1587 (N=N). ^{1}H NMR (400 MHz, Chloroform-*d*) δ 10.33 (br s, 1H, C(6)ON*H*), 8.75 (s, 1H, C(3'')*H*), 8.65 (ddd, *J* = 4.8, 1.7, 0.9 Hz, 1H, C(5)*H*), 8.55 (d, *J* = 5.2 Hz, 1H, C(4'')*H*), 8.31 (dt, *J* = 7.8, 1.1 Hz, 1H, C(2)*H*), 8.14 (d, *J* = 2.2 Hz, 1H, C(2')*H*), 7.96 (td, *J* = 7.8, 1.7 Hz, 1H, C(3)*H*), 7.88 (d, *J* = 8.8 Hz, 1H, C(5')*H*), 7.54 (ddd, *J* = 7.8, 4.8, 1.1 Hz, 1H, C(4)*H*), 7.45 (d, *J* = 5.2 Hz, 1H, C(5'')*H*), 7.10 (dd, *J* = 8.9, 2.2 Hz, 1H, C(6')*H*), 4.13 (s, 3H, OC*H*$_3$). ^{13}C NMR (101 MHz, Chloroform-*d*) δ 162.6 (*C*(6)), 159.8 (*C*(3')), 154.5 (*C*(1'')), 151.4 (*C*(3'')), 149.3 (*C*(1)), 149.0 (*C*(4'')), 148.3 (*C*(5)), 144.2 (*C*(4')), 138.9 (*C*(1')), 138.1 (*C*(3)), 130.2 (*C*(2'')), 127.1 (*C*(4)), 122.7 (*C*(2)), 118.4 (*C*(5')), 112.0 (*C*(6'), *C*(5'')), 103.5 (*C*(2')), 56.6 (O*C*H$_3$). HPLC-PDA-MS: method A was used; RT: 4.05 min, *m/z* (ESI$^+$) 368 (MH$^+$), λ_{max} = 396 nm (*trans* isomer); purity 96 % (254 nm). HRMS (ESI$^+$) found 368.0920, $C_{18}H_{15}N_5O_2Cl$ (MH$^+$) requires 368.0914.

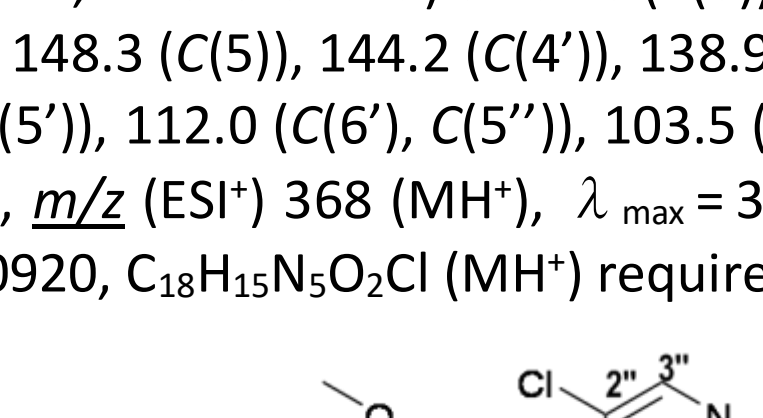

(*E*)-*N*-(3-Chloro-4-((3,5-dichloropyridin-4-yl)diazenyl)-5-methoxyphenyl)picolinamide prosthe6-13. To a stirred solution of azocompound **11** (37.0 mg, 0.112 mmol), picolinic acid (41.2 mg, 0.344 mmol) and HATU (254 mg, 0.670 mmol) in DMF (6.0 mL), TEA (186 µL, 1.34 mmol) was added and then left

stirring 18 h at 40 ºC. Afterwards, the reaction mixture was partitioned with EtOAc and saturated aqueous Na_2CO_3. The aqueous phase was backextracted with EtOAc and the combined organic layers were washed with water, saturated aqueous $NaHCO_3$, water (10x) and then brine. The organic phase was dried over anhydrous $MgSO_4$, filtered and concentrated under reduced pressure. The crude was purified by *flash* column chromatography (Hexanes/EtOAc 80:20) to give the title compound as a dark red solid (9.4 mg, 0.022 mmol, 19%). Mp: 227 – 228 ºC. ν_{max}/cm^{-1} 3321 (N-H), 2923 (C-H), 2853 (C-H), 1691 (C=O), 1567 (N=N). ^{1}H NMR (400 MHz, Chloroform-*d*) 10.29 (br s, 1H, C(6)ON*H*), 8.65 (ddd, *J* = 4.8, 1.7, 0.9 Hz, 1H, C(5)*H*), 8.58 (s, 2H, C(3'')*H*, C(4'')*H*), 8.30 (dt, *J* = 7.9, 1.1 Hz, 1H, C(2)*H*), 7.96 (td, *J* = 7.9, 1.7 Hz, 1H, C(3)*H*), 7.88 (d, *J* = 2.1 Hz, 1H, C(2')*H*), 7.55 (ddd, *J* = 7.9, 4.8, 1.1 Hz, 1H, C(4)*H*), 7.47 (d, *J* = 2.1 Hz, 1H, C(6')*H*), 3.99 (s, 3H, OC*H₃*). ^{13}C NMR (101 MHz, Chloroform-*d*) δ 162.6 (*C*(6)), 154.7 (*C*(1'')), 153.6 (*C*(3')), 149.0 (*C*(1)), 148.9 (*C*(3''), *C*(4'')), 148.3 (*C*(5)), 141.7 (*C*(4')), 138.2 (*C*(3)), 135.5 (*C*(1')), 134.7 (*C*(5')), 127.3 (*C*(4)), 123.3 (*C*(2''), *C*(5'')), 122.8 (*C*(2)), 113.4 (*C*(6')), 102.4 (*C*(2')), 57.0 (O*C*H₃). HPLC-PDA-MS: method A was used; RT: 4.56 min, *m/z* (ESI$^+$) 438 (MH$^+$), λ_{max} = 488 nm (*trans* isomer); purity 96 % (254 nm). HRMS (ESI$^+$) found 436.0131, $C_{18}H_{13}N_5O_2Cl_3$ (MH$^+$) requires 436.0135.

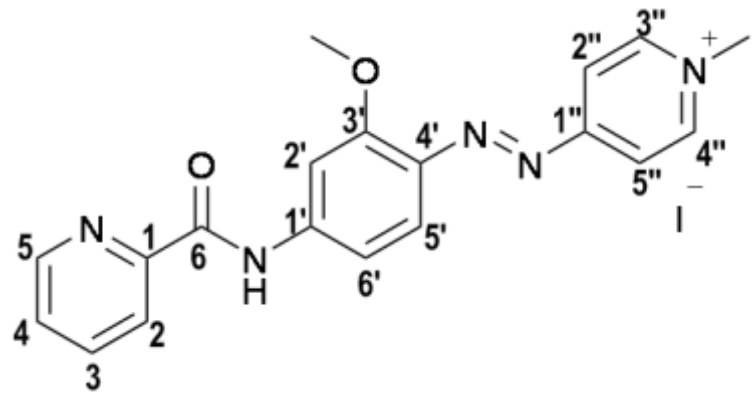

(*E*)-4-((2-Methoxy-4-(picolinamido)phenyl)diazenyl)-1-methylpyridin-1-ium iodide prosthe6-14. To a stirred solution of azocompound **prosthe6-15** (17.5 mg, 0.052 mmol) in anhydrous DCM (0.80 mL) at room temperature, methyl iodide (14 µL, 0.231 mmol) was added. The solution was stirred at room temperature for 18 h. The precipitated solid was filtered and then washed with cold DCM to afford the title compound as a dark solid (9.4 mg, 0.020 mmol, 38 %) pure and no further purification was required. ν_{max}/cm^{-1} 3317 (N-H), 3045 (C-H), 1690 (C=O), 1577 (N=N). ^{1}H NMR (400 MHz, DMSO-*d6*) δ 11.21 (br s, 1H, C(6)ON*H*), 9.08 (d, *J* = 6.5 Hz, 2H, C(3'')*H*, C(4'')*H*), 8.80 (d, *J* = 4.8 Hz, 1H, C(5)*H*), 8.31 - 8.23 (m, 2H, C(2'')*H*, C(5'')*H*), 8.21 (d, *J* = 7.8 Hz, 1H, C(2)*H*), 8.16 - 8.09 (m, 2H, C(3)*H*, C(2')*H*), 7.84 (s, 2H, C(6')*H*, C(5')*H*), 7.75 (ddd, J = 7.8, 4.8, 1.3 Hz, 1H, C(4)*H*), 4.38 (s, 3H, C*H₃*), 4.05 (s, 3H, OC*H₃*). ^{13}C NMR (101 MHz, DMSO-*d6*) δ 163.4 (*C*(6)), 160.5 (*C*(3')), 160.4 (*C*(1'')), 149.2 (*C*(1)), 148.6 (*C*(5)), 147.7 (*C*(3''), *C*(4'')), 147.4 (*C*(4')), 138.4 (*C*(3)), 138.1 (*C*(1')), 127.6 (*C*(4)), 122.8 (*C*(2)), 119.4 (*C*(2''), *C*(5'')), 117.6 (*C*(5')), 112.6 (*C*(6')), 104.4 (*C*(2')), 56.3 (O*C*H₃), 47.5 (*C*H₃). HPLC-PDA-MS: method A was used; RT: 2.83 min, *m/z* (ESI$^+$) 348 (MH$^+$), λ_{max} = 434 nm (*trans* isomer); purity 90 % (254 nm). HRMS (ESI$^+$) found 348.1461, $C_{19}H_{18}N_5O_2$ (MH$^+$) requires 348.1461.

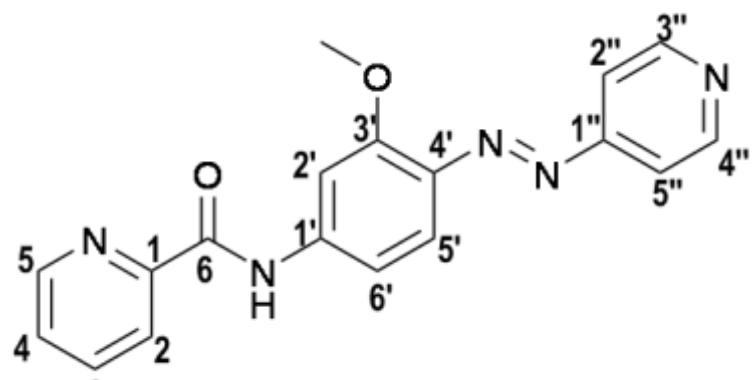

(*E*)-*N*-(3-Methoxy-4-(pyridin-4-yldiazenyl)phenyl)picolinamide prosthe6-15. To a stirred solution of azocompound **12** (20.0 mg, 0.088 mmol), picolinic acid (16.2 mg, 0.131 mmol) and HATU (100 mg, 0.263 mmol) in DMF (3.0 mL), TEA (73 µL, 0.526 mmol) was added and then left stirring 72 h at room temperature. Afterwards, the reaction mixture was partitioned with EtOAc and saturated aqueous Na_2CO_3. The aqueous phase was backextracted with EtOAc and the combined organic layers were washed with water, saturated aqueous $NaHCO_3$, water (10x) and then brine. The organic phase was dried over anhydrous $MgSO_4$, filtered and concentrated under reduced pressure. The crude was purified by *flash* column chromatography (Hexanes/EtOAc 50:50) to give the title compound as an orange solid (23.7 mg, 0.071 mmol, 81 %). ν_{max}/cm^{-1} 3340 (N-H), 2921 (C-H), 2849 (C-H), 1692 (C=O),

1583 (N=N). ^{1}H NMR (400 MHz, Chloroform-*d*) δ 10.31 (br s, 1H, C(6)ON*H*), 8.77 (d, *J* = 5.2 Hz, 2H, C(3'')*H*, C(4'')*H*), 8.65 (ddd, *J* = 4.8, 1.7, 0.9 Hz, 1H, C(5)*H*), 8.32 (dt, *J* = 7.9, 1.1 Hz, 1H, C(2)*H*), 8.09 (d, *J* = 2.1 Hz, 1H, C(2')*H*), 7.96 (td, *J* = 7.9, 1.7 Hz, 1H, C(3)*H*), 7.82 (d, *J* = 8.8 Hz, 1H, C(5')*H*), 7.73 - 7.64 (m, 2H, C(2'')*H*, C(5'')*H*), 7.54 (ddd, *J* = 7.9, 4.8, 1.2 Hz, 1H, C(4)*H*), 7.14 (dd, *J* = 8.8, 2.1 Hz, 1H, C(6')*H*), 4.12 (s, 3H, OCH_3). ^{13}C NMR (101 MHz, Chloroform-*d*) δ 162.5 (*C*(6)), 159.3 (*C*(3')), 158.1 (*C*(1'')), 151.3 (*C*(3''), *C*(4'')), 149.4 (*C*(1)), 148.3 (*C*(5)), 143.6 (*C*(4')), 138.5 (*C*(1')), 138.1 (*C*(3)), 127.1 (*C*(4)), 122.7 (*C*(2)), 117.9 (*C*(5')), 116.5 (*C*(2''), *C*(5'')), 111.9 (*C*(6')), 103.6 (*C*(2')), 56.6 (O*C*H$_3$). HPLC-PDA-MS: method A was used; RT: 3.10 min, *m/z* (ESI$^+$) 334 (MH$^+$), λ_{max} = 420 nm (*trans* isomer); purity 99 % (254 nm). HRMS (ESI$^+$) found 334.1318, $C_{18}H_{16}N_5O_2$ (MH$^+$) requires 334.1304.

Synthesis of (2R, 4R)-4-amino-1-benzylpyrrolidine-2,4-dicarboxylic acid (1-Bz-APDC)

Compound 1-Bz-APDC was synthesized as reported[1].

2. Photochemical characterization of prosthe6 compounds (absorption, slow/fast relaxation and photostability).

Prosthe6 UV-Vis absorption spectra, aqueous solubility and relaxation time measurement

A collection of samples was prepared with 25 µM of prosthe6 (50 µM in the case of compound prosthe6-**10**) in different solvent configurations ranging from 100 % DMSO to 20 % DMSO (by volume) in PBS at 25 ºC. UV-Vis absorbance spectra of the samples was measured between 300 nm and 600 nm with 2 nm fixed intervals in 96-well transparent plates (200 µL of compound solution/well) using the Spark microplate reader. Illumination at the different wavelengths (380, 455, 470, 550 nm) was achieved using the using the LED array plate (LEDA Teleopto) during 3 min. The aqueous solubility was obtained by comparison of the UV-Vis absorbance spectra in the dark at different DMSO percentages to that of pure DMSO. The approximated aqueous solubility was selected by choosing the DMSO percentage that rendered the spectra similar in band intensity to that of the DMSO spectra.
Thermal relaxation was studied at 25 ºC by prolonged absorbance measuring (in dark conditions) after samples (25-50 µM in DMSO) had been subjected to 3 min illuminations at their corresponding optimal *trans* to *cis* wavelength. Samples were illuminated using the LED array plate (LEDA Teleopto) system and measurements were collected using the Spark microplate reader. The half-life of the compounds was calculated by plotting absorbance readings at a fixed wavelength *versus* time and then fitting the obtained curve to an exponential decay function using GraphPad prism. The measured wavelength corresponded to the point where maximum difference in absorbance was observed between *trans* and *cis* isomers.

Prosthe6-12 and 15 relaxation time measurement in PBS: DMSO 99:1

For prosthe6-**12** and **15** having relaxation times in the order of a few seconds (> 1 s), the thermal back-isomerization in the dark was monitored by steady state UV-Vis absorption spectroscopy using a 8453 Agilent spectrophotometer coupled with a LED395 (Chanzon, 380-415 nm) for *in situ* photoexcitation. In all the cases, these measurements were conducted in PBS:DMSO 99:1 solutions and 1-cm optical path cuvettes at room temperature (22ºC). The *cis* lifetimes in the dark were determined by fitting the time variation of the absorption (at 395 nm and 381 nm respectively for prosthe6-**12** and **15**) of the sample after irradiation with a monoexponential functionusing GraphPad Prism software.

Prosthe6-14 relaxation time measurement in PBS: DMSO 99:1

Fast *cis*–*trans* thermal isomerization processes were studied by transient absorption spectroscopy by means of a ns LKII laser flash-photolysis spectrometer (Applied Photophysics, Surrey, UK) equipped with a Xe lamp as probe source, a monochromator, and a photomultiplier (PMT) tube (Hamamatsu Photonics, Hamamatsu, JP). A solution of prosthe6-**14** in PBS: DMSO 99:1 was excited at 355 nm by a 20 ns pulsed laser beam arising from the third harmonic unit of a Nd:YAG Brilliant laser (Quantel, Les Ulis, FR). A 500 MHz oscilloscope (Agilent Technologies, Inc.) transferred the signal received from the

PMT to an Accorn PCRisk station. The variation of absorption as measured at t=0 s in the 300-500 nm region mirrors the steady-state UV/Vis absorption spectra of the *trans* isomers and indicates formation of the *cis* isomers. The thermal back-isomerization kinetics of the photogenerated *cis* isomers were measured at 430 nm. Half-life was determined fitting the experimental data to a monoexponential decay model with the GraphPad Prism software.

**Compound**	**Wavelengths used for *trans*-*cis*/*cis*-*trans* (nm in DMSO)**	**Aqueous sample (% DMSO in PBS)[a]**	**Wavelengths used for *trans*-*cis*/*cis*-*trans* (nm in aqueous media)**	**$t_{½}$ in DMSO**	**$t_{½}$ in aqueous media**
**prosthe6-1**	380 / 470	40	380 / 470	> 16 h	2.5 h
**prosthe6-2**	380 / 550	60	ND	2 min	ND
**prosthe6-3**	380 / 470	20	380 / -	> 16 h	< 10 s
**prosthe6-4**	380 / 470	20	380 / -	> 16 h	< 10 s
**prosthe6-5**	380 / 550	40	ND	6 min	ND
**prosthe6-6**	ND	60	ND	ND	ND
**prosthe6-7**	ND	40	ND	ND	ND
**prosthe6-8**	380 / 550*	20	380 / 550*	32 min	27 min
**prosthe6-9**	380 / 550	60	380 / 550	77 min	4 min
**prosthe6-10**	380 / 470	60	ND	65 min	ND
**prosthe6-11**	405 / 365	20	ND	8.9 min	ND
**prosthe6-12**	380 / 470	20	395 / -	10 min	ND (4.3 s)[#]
**prosthe6-13**	380 or 550 / 470	80	365 / -	1 min	1 min
**prosthe6-14**	ND	20	355 / -	ND	ND (1 ms)[#]
**prosthe6-15**	380 / 470	20	395 / -	4 min	ND (1.9 s)[#]

Table S1. The table shows the chemical/photochromic properties of the prosthe6 compounds. It includes the *trans*-to-*cis* and *cis*-to-*trans* wavelengths of prosthe6 compounds in both DMSO and aqueous media, and the thermal relaxation lifetimes in DMSO and aqueous solutions (slow for prosthe6-**1**, faster for the others).
[a] Percentage of DMSO used to obtain a solubilized 25 µM sample. * Any of the assayed wavelengths rendered an optimal *cis*-to-*trans* isomerisation. N.D. = could not be determined because of the fast thermal relaxation of the compounds. [#] Relaxation times determined by transient absorption spectroscopy in PBS:DMSO 99:1 (see Figure 2efg).

Compounds<br><br>MW and aqueous solubility	UV-Vis absorption spectra in DMSO	Thermal relaxation in DMSO
**prosthe6-1**<br><br>366.8 g/mol<br><br>40 % DMSO in PBS (25 µM)	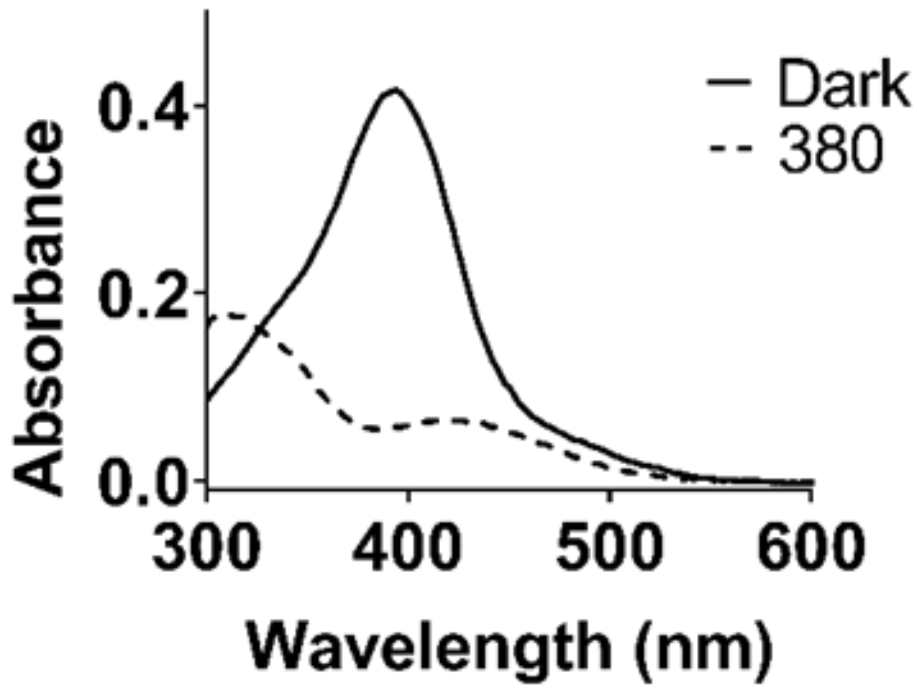<br>25 µM	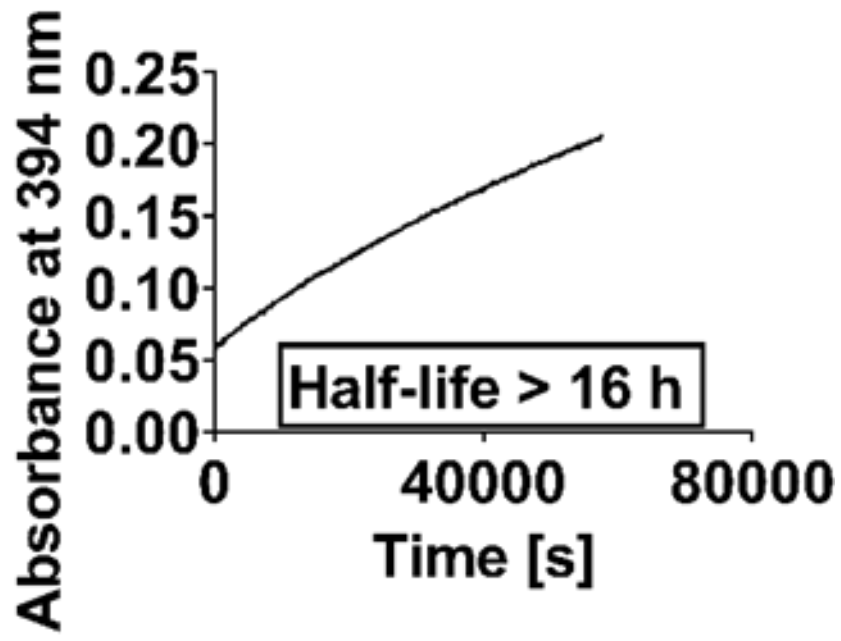<br>25 µM
**prosthe6-2**<br><br>411.8 g/mol<br><br>100 % DMSO (25 µM)	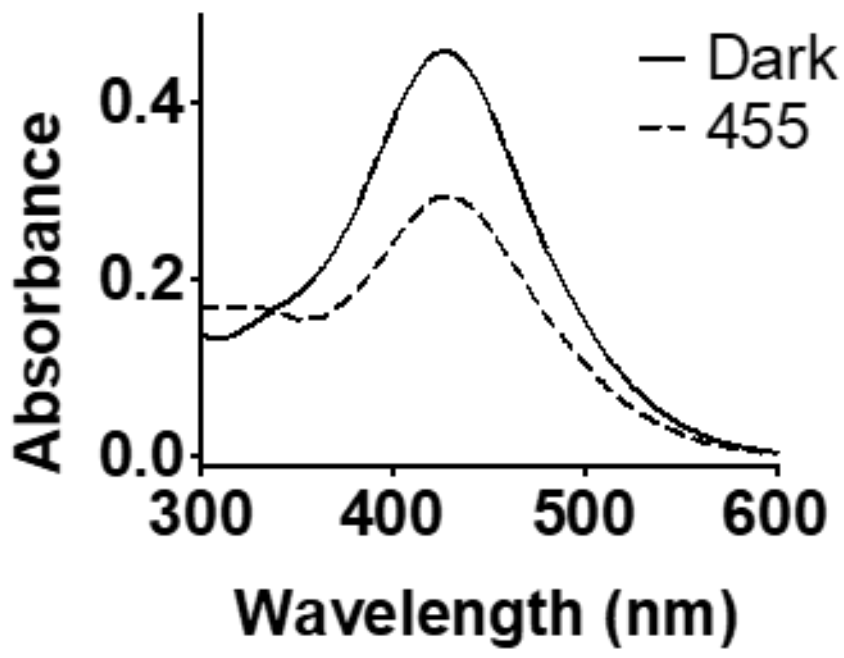<br>25 µM	25 µM<br>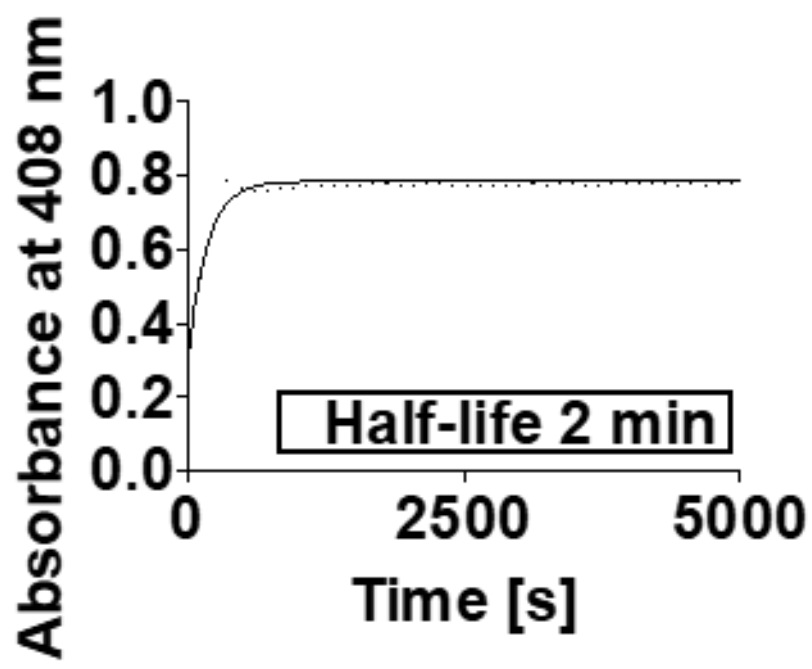
**prosthe6-3**<br><br>458.2 g/mol<br><br>100 % DMSO (25 µM)	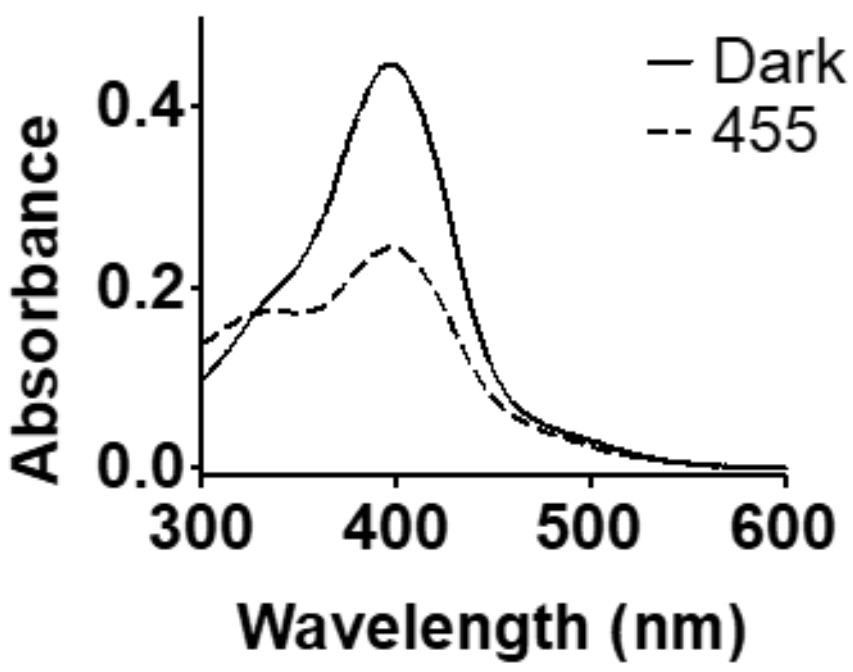<br>25 µM	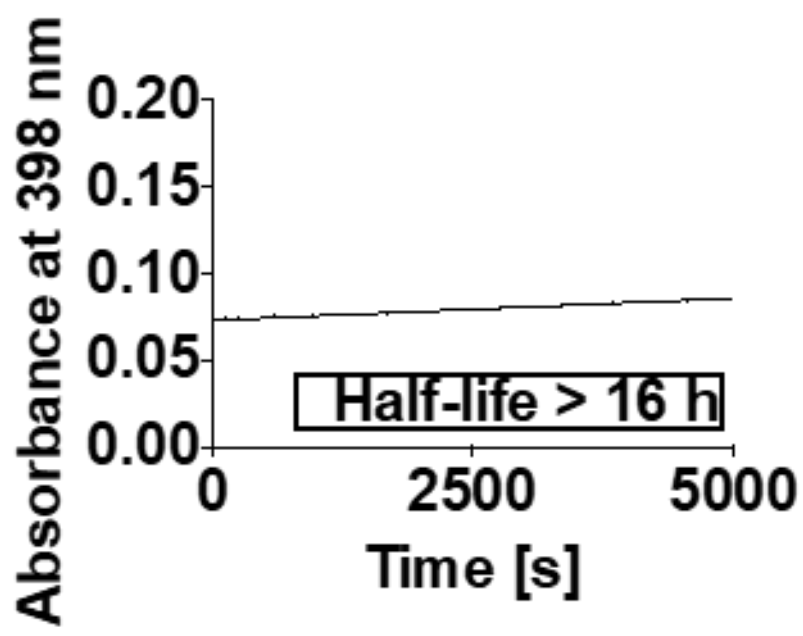<br>25 µM

**Compounds**<br><br>**MW and aqueous solubility**	**UV-Vis absorption spectra in DMSO**	**Thermal relaxation in DMSO**
**prosthe6-4**<br><br>537.1 g/mol<br><br>100 % DMSO (25 µM)	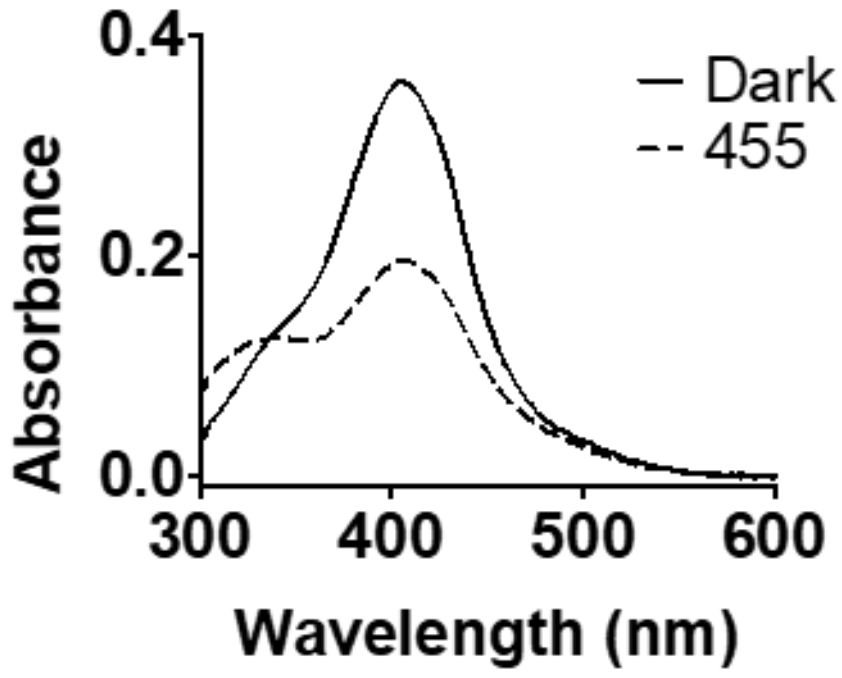<br>25 µM	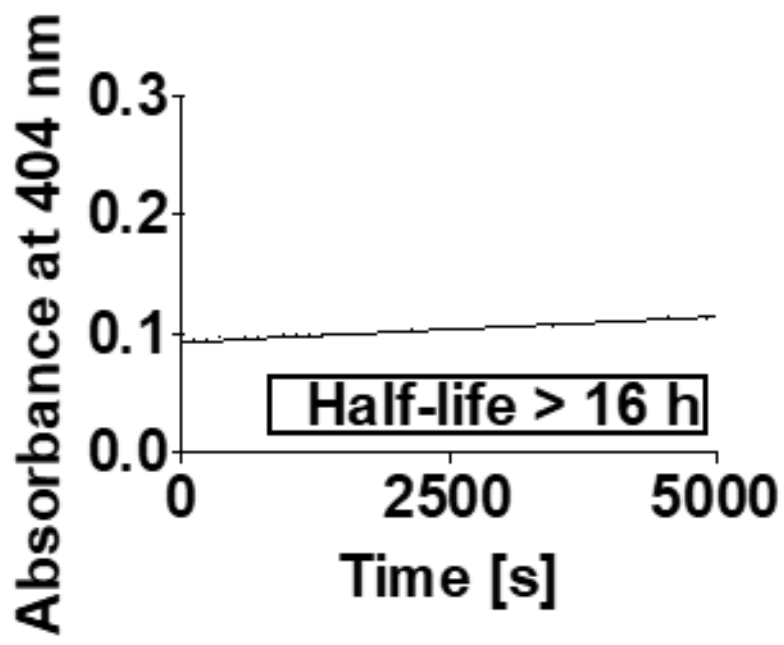<br>25 µM
**prosthe6-5**<br><br>339.3 g/mol<br><br>100 % DMSO (25 µM)	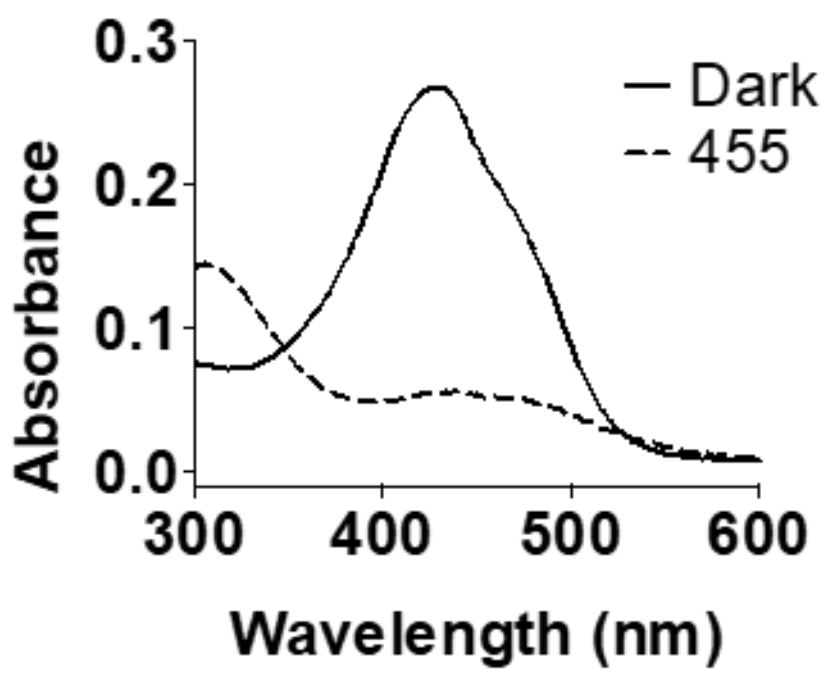<br>25 µM	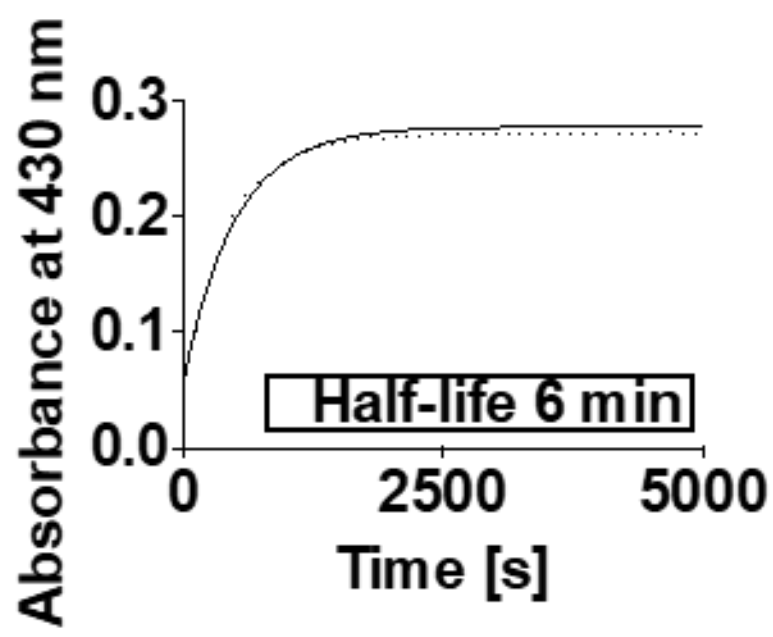<br>25 µM
**prosthe6-6**<br><br>334.3 g/mol<br><br>100 % DMSO (25 µM)	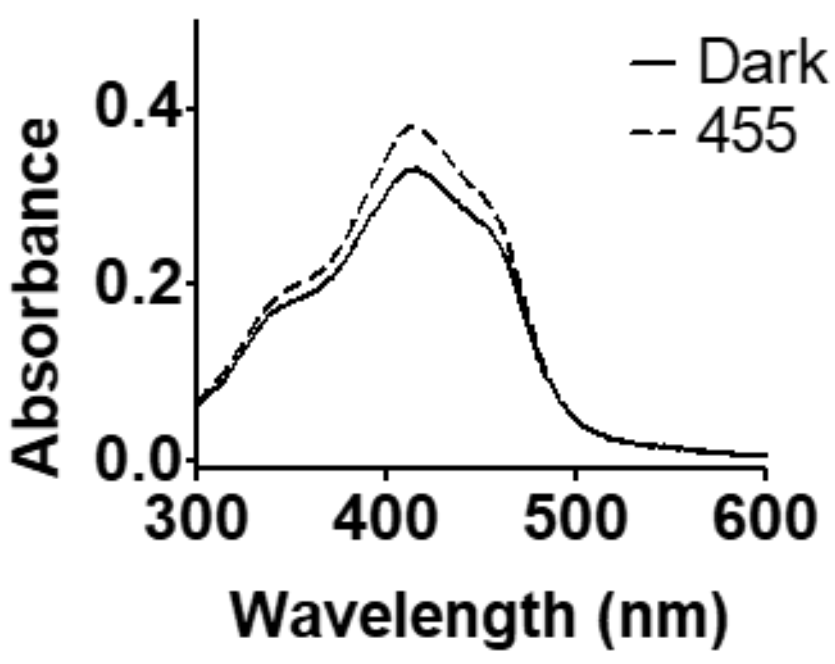<br>25 µM	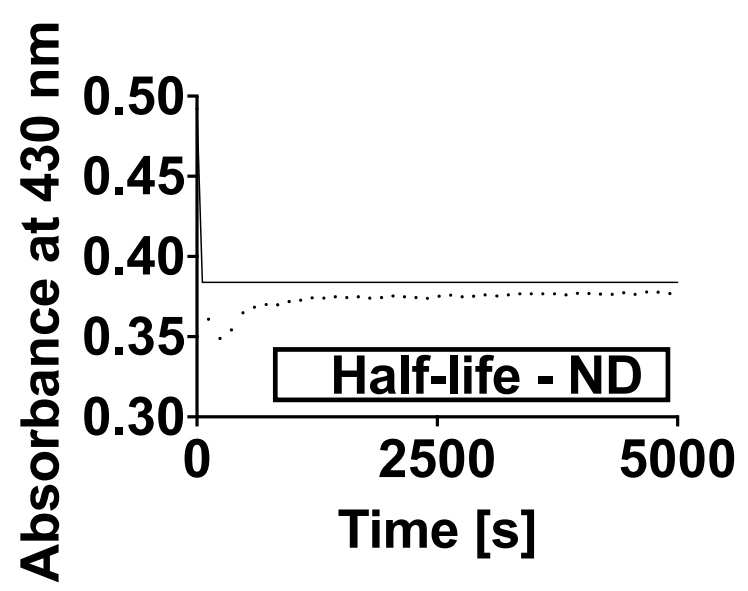<br>25 µM

Compounds<br><br>MW and aqueous solubility	UV-Vis absorption spectra in DMSO	Thermal relaxation in DMSO
**prosthe6-7**<br><br>352.2 g/mol<br><br>100 % DMSO (25 µM)	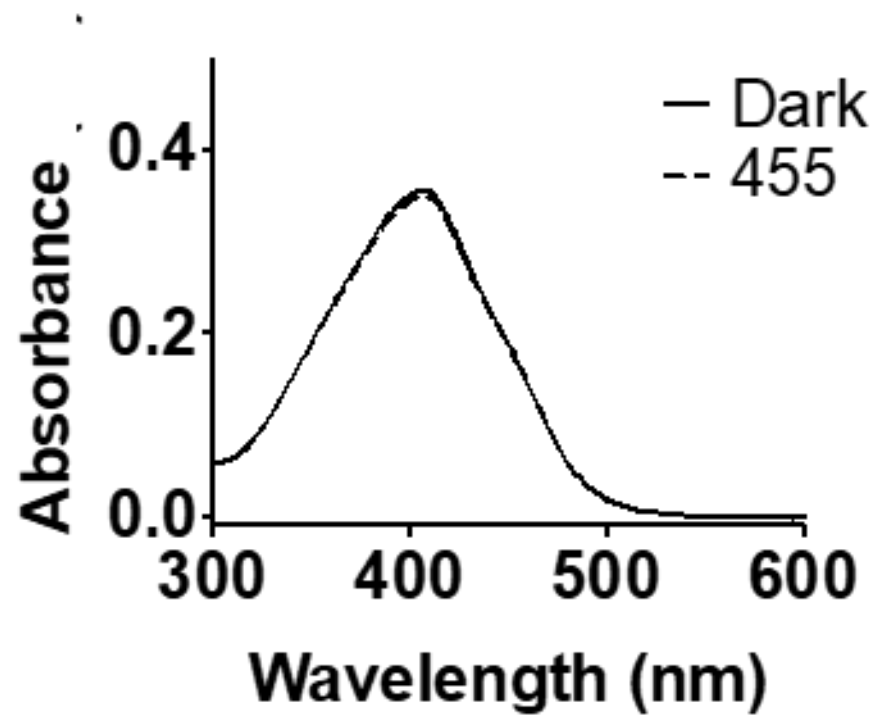<br>25 µM	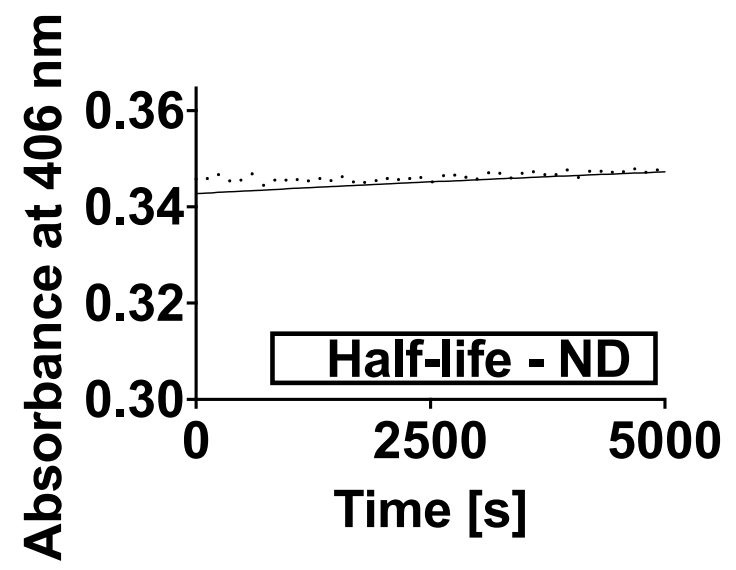<br>25 µM
**prosthe6-8**<br><br>401.4 g/mol<br><br>80 % DMSO in PBS (25 µM)	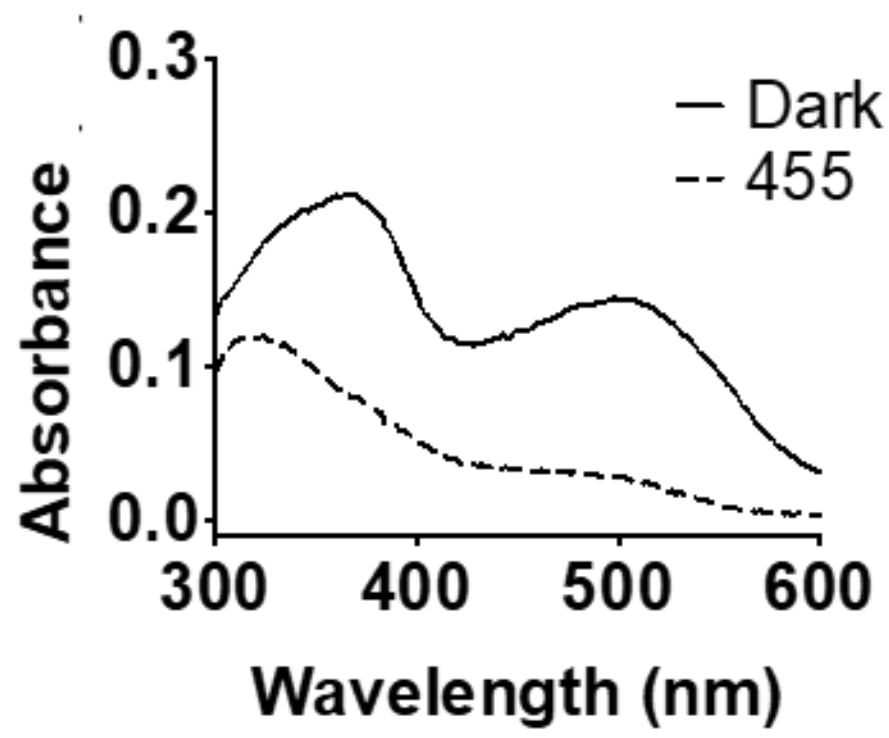<br>25 µM	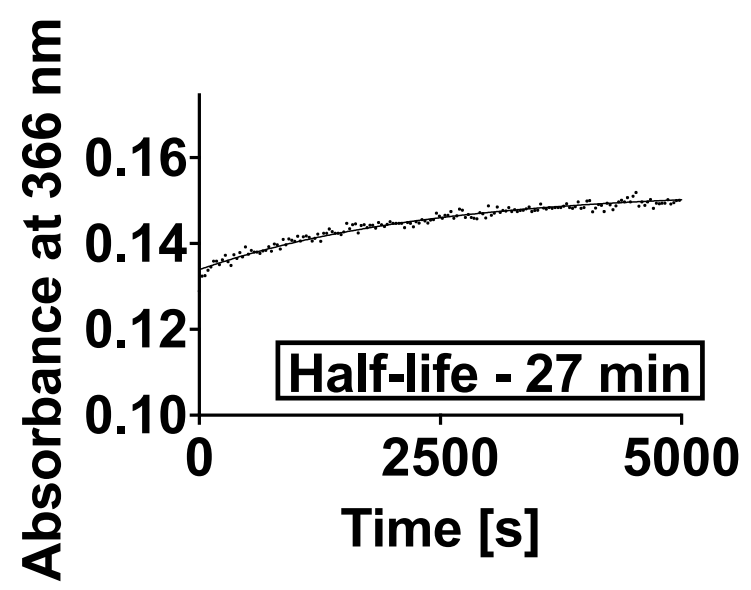<br>25 µM
**prosthe6-9**<br><br>397.8 g/mol<br><br>60 % DMSO in PBS (25 µM)	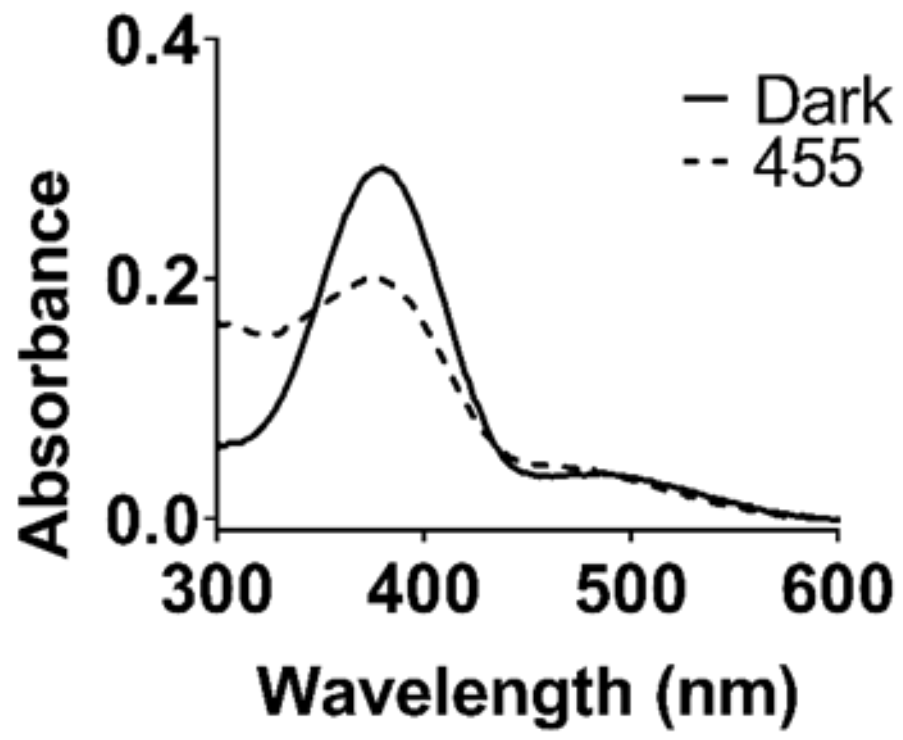<br>25 µM	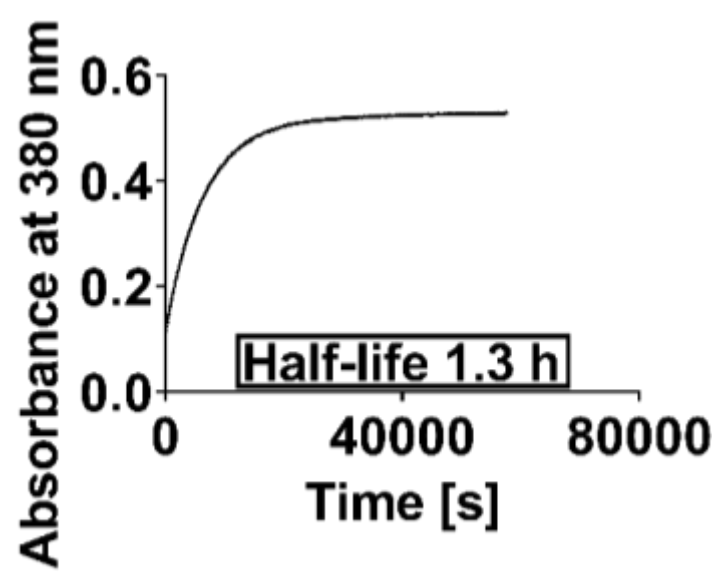<br>25 µM

<table>
<tr><th>Compounds

MW and aqueous solubility</th><th>UV-Vis absorption spectra in DMSO</th><th>Thermal relaxation in DMSO</th></tr>
<tr><td>prosthe6-10

421.4 g/mol

60 % DMSO in PBS (25 µM)</td><td>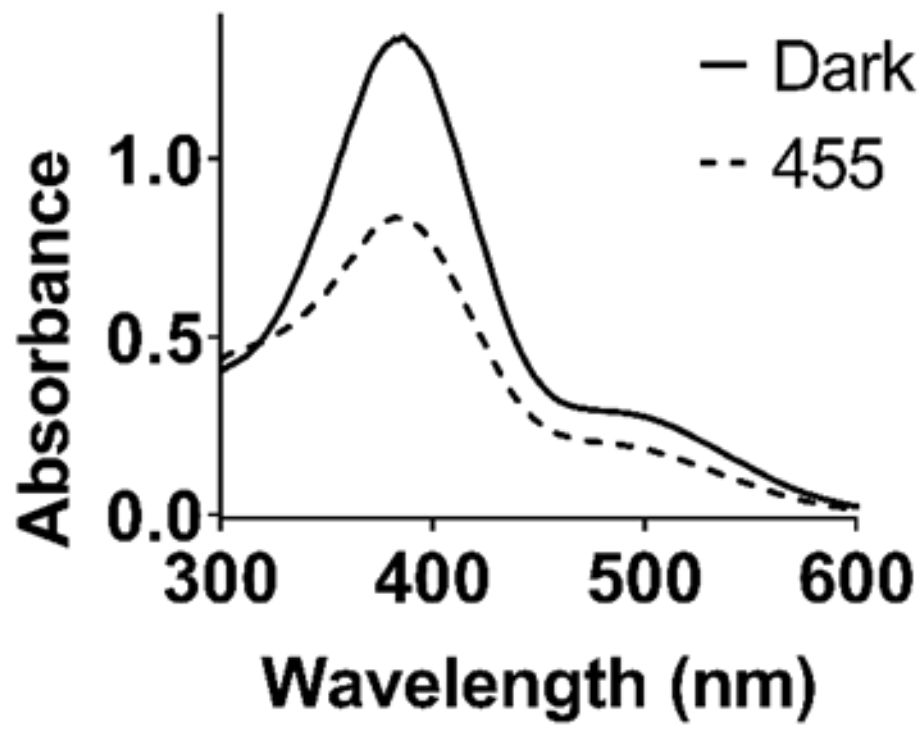

50 µM</td><td>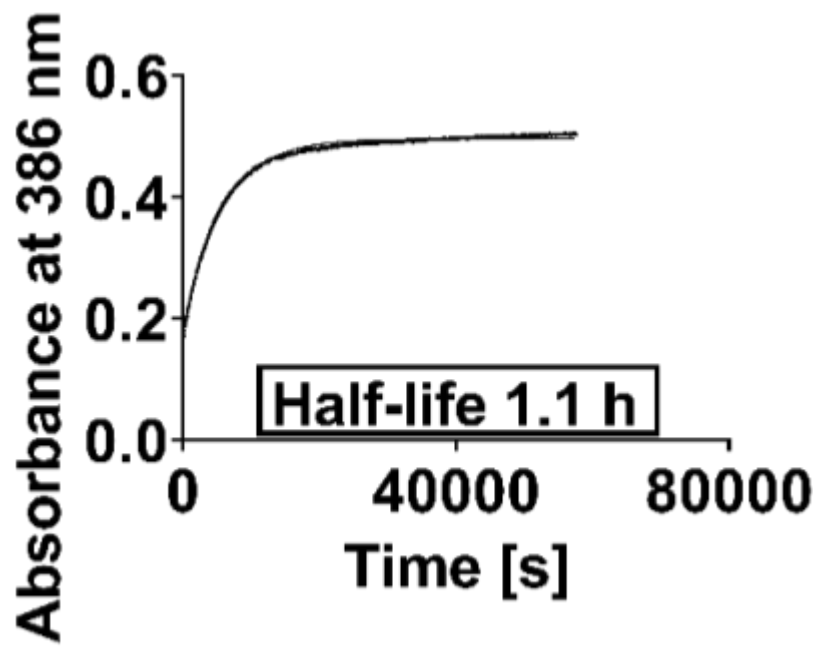

25 µM</td></tr>
<tr><td>prosthe6-11

405.5 g/mol

20 % DMSO in PBS (25 µM)</td><td>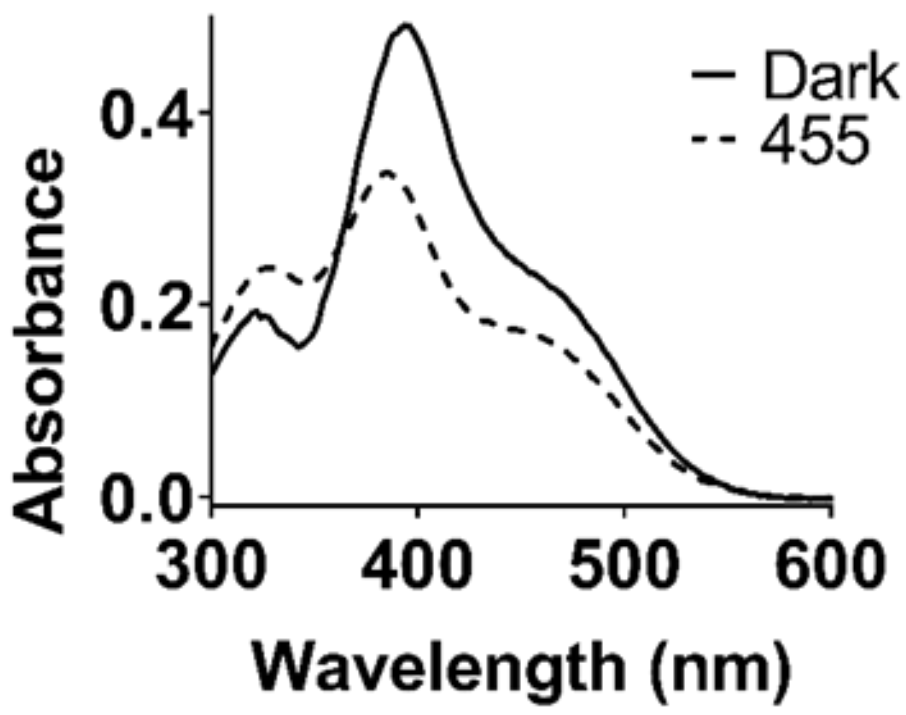

25 µM</td><td>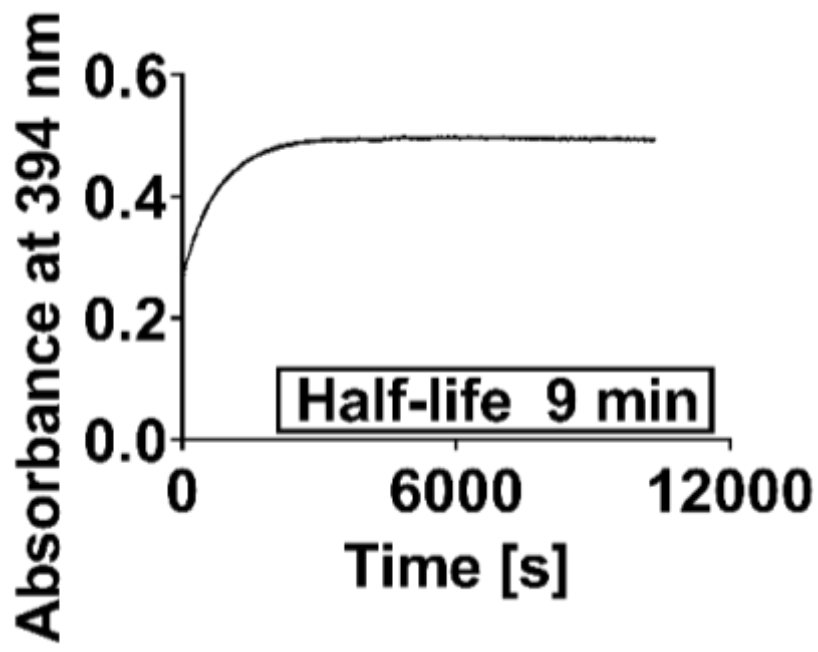

25 µM</td></tr>
<tr><td>prosthe6-12

367.8 g/mol

20 % DMSO in PBS (25 µM)</td><td>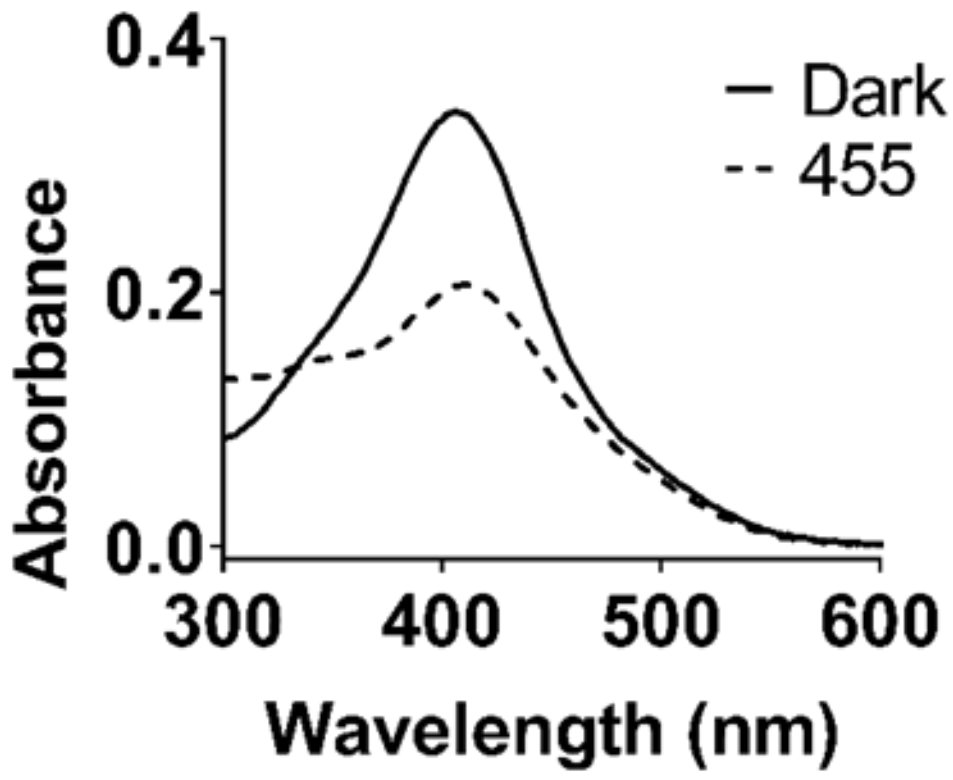

25 µM</td><td>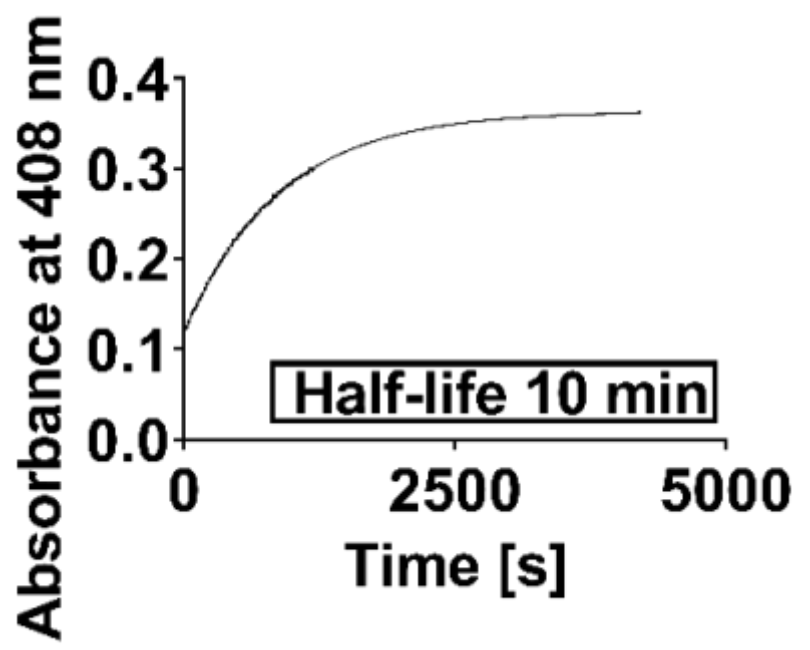

25 µM</td></tr>
</table>

Compounds<br><br>MW and aqueous solubility	UV-vis absorption spectra in DMSO	Thermal relaxation in DMSO
**prosthe6-13**<br><br>436.7 g/mol<br><br>80 % DMSO in PBS (25 µM)	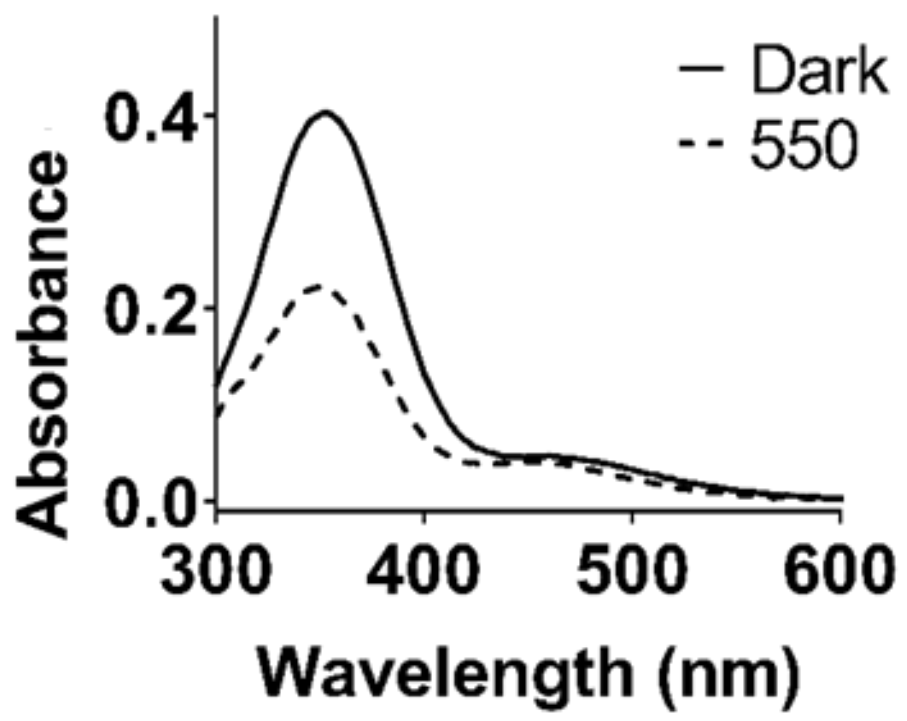<br>25 µM	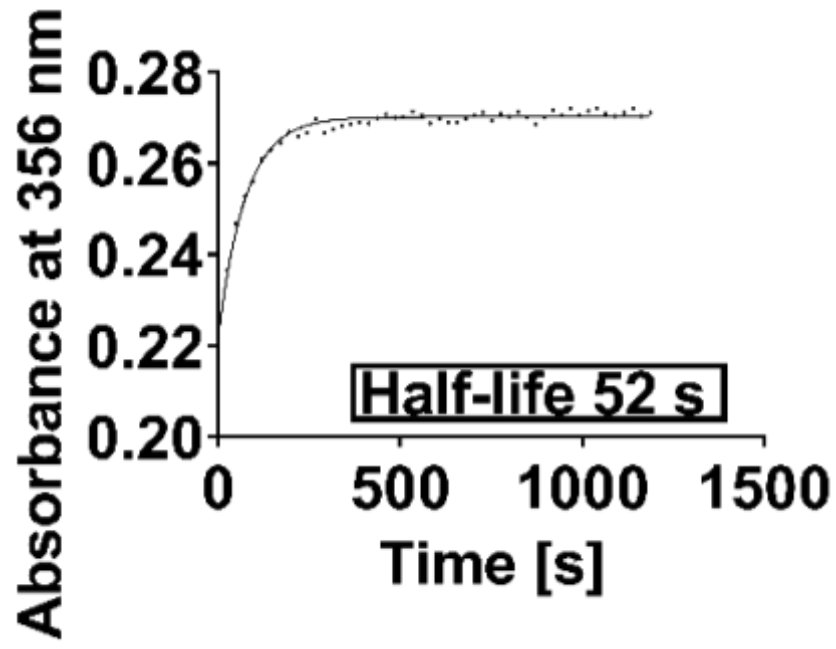<br>25 µM
**prosthe6-14**<br><br>476.3 g/mol<br><br>20 % DMSO in PBS (25 µM)	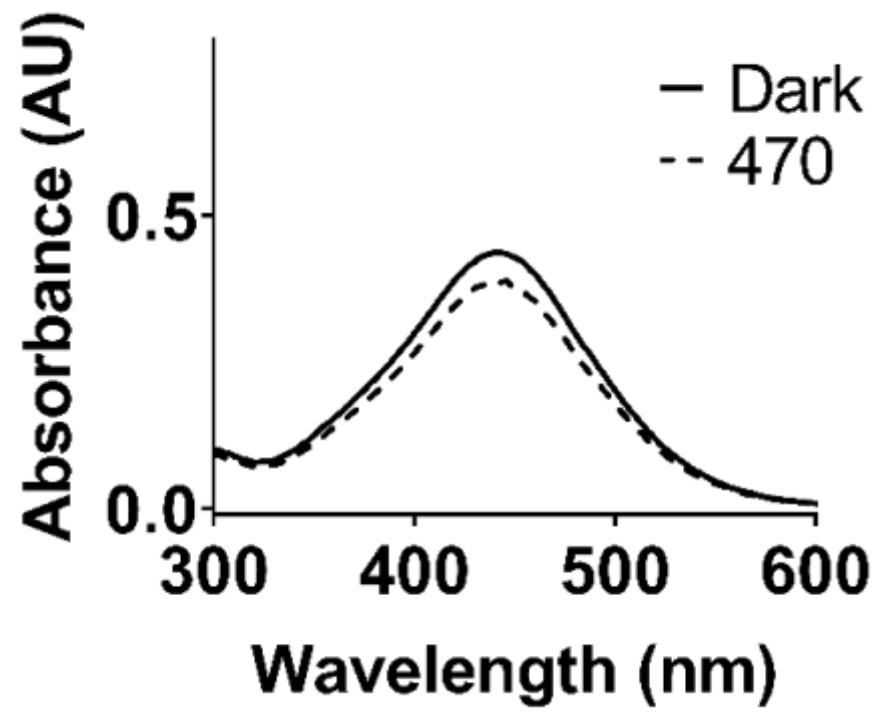<br>25 µM	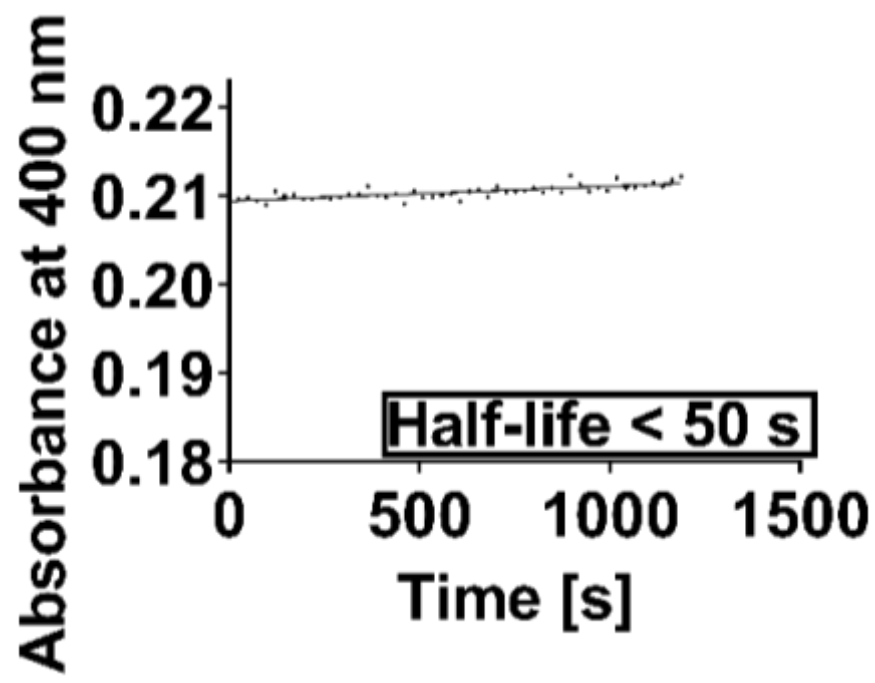<br>25 µM
**prosthe6-15**<br><br>333.3 g/mol<br><br>20 % DMSO in PBS (25 µM)	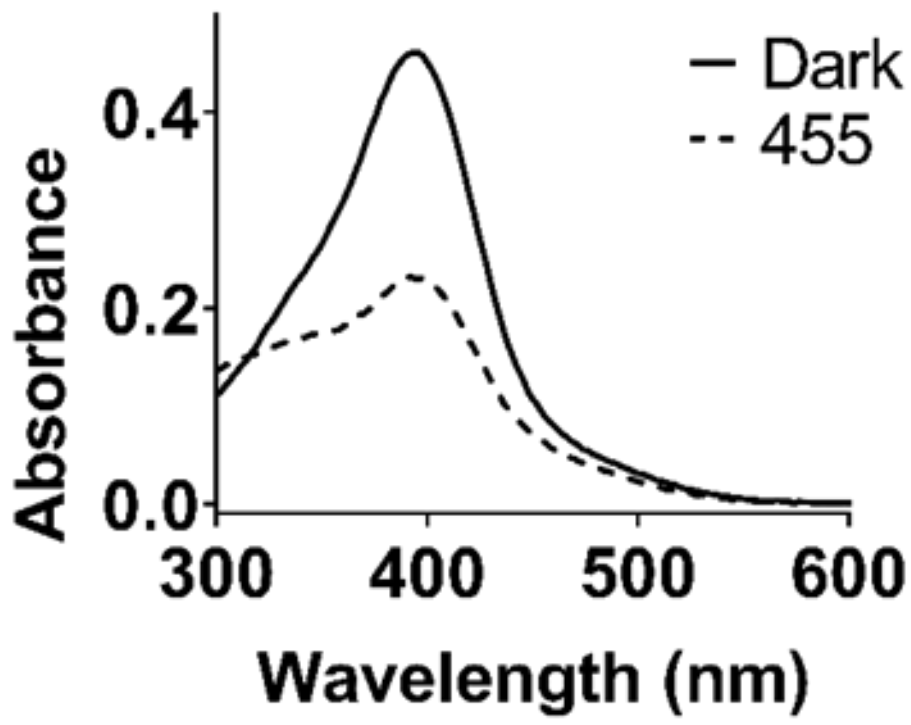<br>25 µM	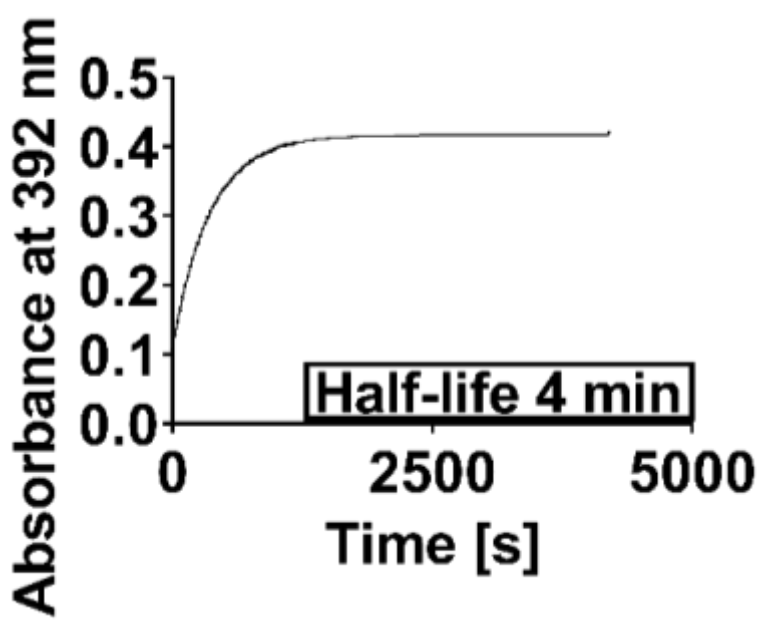<br>25 µM

Figure S4. Molecular weight (MW), aqueous solubility, Ultraviolet (UV)-Vis absorption spectra in DMSO and thermal relaxation in DMSO of prosthe6 compounds.

Assessment of prosthe6-12 photostability

Photostability of prosthe6-**12** was evaluated by measuring the absorption spectra (Shimadzu UV-1800 UV-vis spectrophotometer) of a 100 µM solution in a sealed cuvette to avoid solvent evaporation before and after irradiation with a 405 nm LED. The photoconversion protocol consisted of 1000 cycles, each one composed by 10 seconds of irradiation at 120 mW of intensity measured with a power meter (PM101 - Power Meter Interface with USB, RS232, UART, and Analog Operation, Thorlabs GmbH; S470C - Thermal Power Sensor Head, Volume Absorber, 0.25 - 10.6 µm, 100 µW - 5 W, Ø15 mm, Thorlabs GmbH) followed by 10 seconds of darkness. Area under the curve (AUC) of both absorption spectra were computed using Graphpad Prism Software version 9, and a ratio to assess the percentage of degradation of the compound was calculated as AUC after irradiation / AUC before irradiation. The observed results (5 % decrease in AUC after 1000 cycles of photoswitching) show that the compound is stable to photobleaching and has high fatigue resistance.

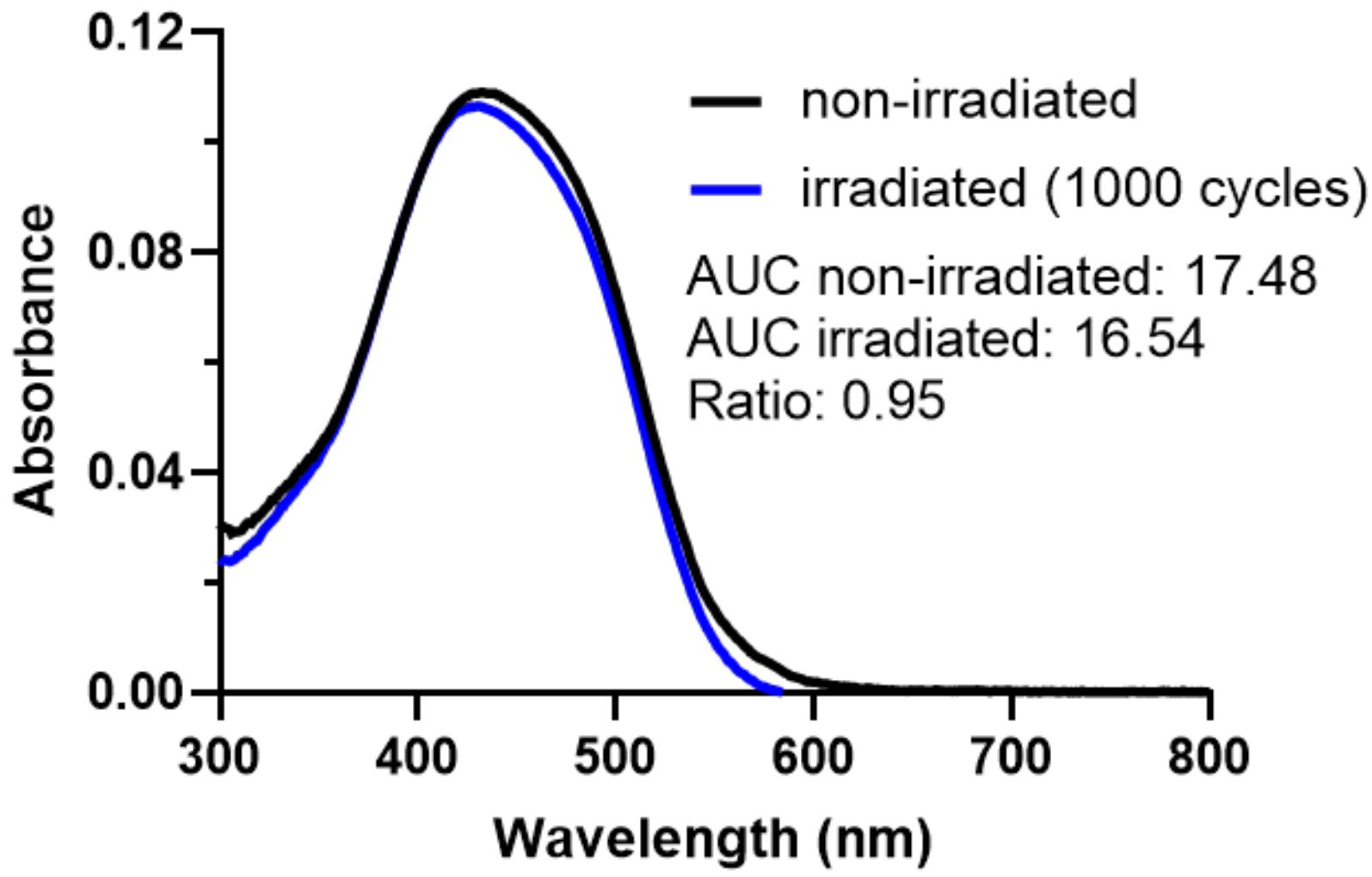

Figure S5. Absorption spectra of prosthe6-**12** 100 µM before and after 1000 cycles of irradiation with 405 nm light. The absorption spectra barely vary after the stimulation.

3. *In vitro* photopharmacological characterization in overexpressed metabotropic glutamate 6 ($mGlu_6$) receptors

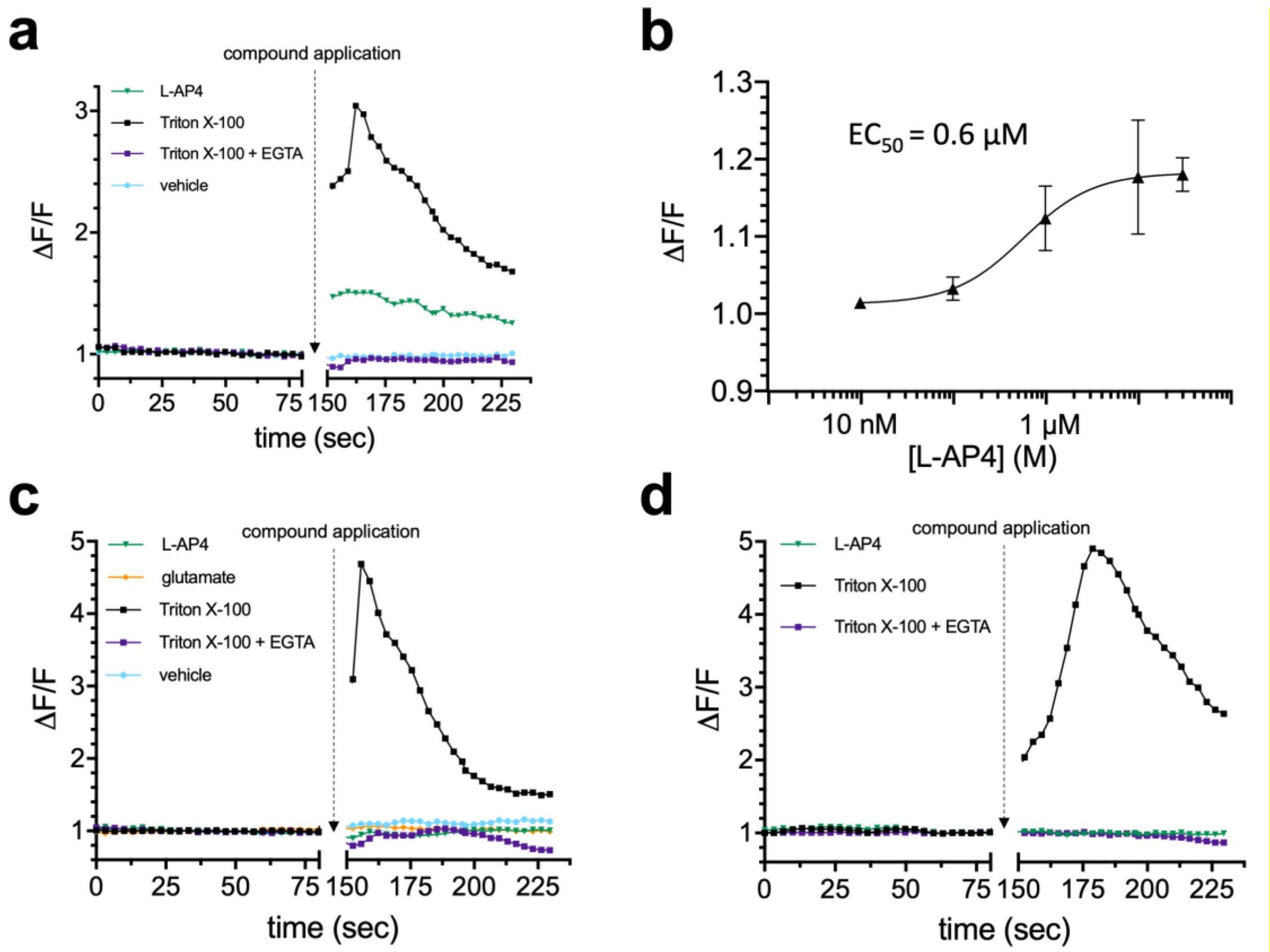

Figure S6. Multiwell plate assay controls. (a) Positive and negative controls of the fluorescence response in Human embryonic kidney (HEK) cells co-expressing wild-type (wt) mouse metabotropic glutamate 6 ($mGlu_6$), $G_{q/i}$ and the calcium indicator GCaMP6s. Fluorescence time course (ex. 485 nm, em. 535 nm) in an individual well upon application of 10 µM L-2-amino-4-phosphonobutyric acid (L-AP4) ($mGlu_6$ orthosteric agonist) is represented in green. Triton X-100 (0.2 %, black) is a non-ionic surfactant that was used to lyse cell membrane, leading to the release of intracellular Ca^{2+} ions into the extracellular medium and resulting in maximal fluorescence intensity. Addition of a mix of Triton X-100 (0.2 %) and 10 mM ethylene glycol-bis(β-aminoethyl ether)-N,N,N′,N′-tetraacetic acid (EGTA; purple) led to minimal fluorescence intensity, attributed to the Ca^{2+} chelating effect of EGTA (negative control). Vehicle (2 % DMSO) is shown in light blue. **(b)** The peak response of multiple wells was averaged for each independent experiment (n=3) and used to build dose-response curves. The reported EC_{50} value of L-AP4 is 0.6 – 0.9 µM, in agreement with results. Each point represents the mean ± SEM of at least three independent experiments. **(c)** Positive and negative controls of fluorescence response in HEK cells co-expressing mutant T171A $mGlu_6$, $G_{q/i}$ and GCaMP6s. The T171A $mGlu_6$ mutant is not sensitive to orthosteric agonists. Consequently, L-AP4 (green) and glutamate (orange) did not elicit any responses. Vehicle and the mix Triton X-100 (0.2 %) + EGTA (10 mM) are represented in light blue and purple, respectively, serving as negative controls, while Triton X-100 as positive control. **(d)** Fluorescence responses of cells expressing only GCaMP6s. The calcium released after Triton application was successfully detected by the calcium indicator, confirming its ability to detect increases in calcium concentrations. No responses were elicited upon application of L-AP4, confirming the absence of receptor expression.

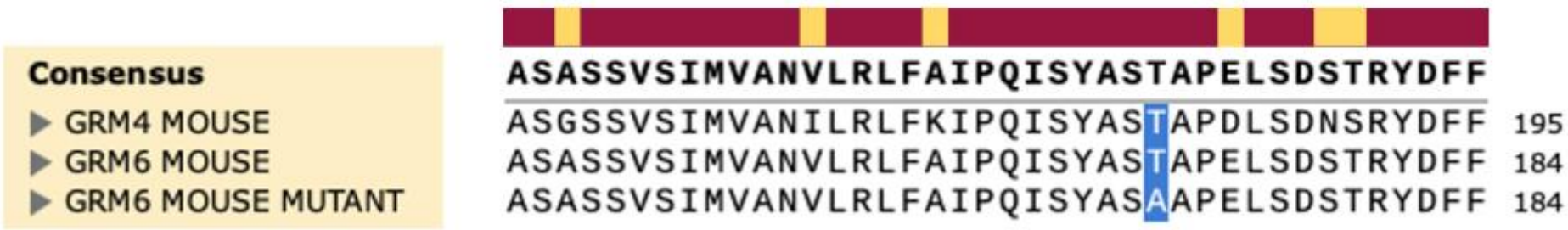

Figure S7. Multiple sequence alignment of mouse metabotropic glutamate 4 ($mGlu_4$) wild-type (*wt*), mouse metabotropic glutamate 6 ($mGlu_6$) wt and mouse mutant $mGlu_6$. Alignment was conducted utilizing SnapGene software. The alignment highlights Thr^{182} of $mGlu_4$ (first line)[2] and its corresponding amino acid in $mGlu_6$, Thr^{171} (second line). In the mutant $mGlu_6$ sequence, Thr^{171} was substituted with alanine (third line). This mutation renders the $mGlu_6$ receptor insensitive to the orthosteric agonists. The consensus sequence is depicted in bold, with regions of complete conservation of amino acids between $mGlu_4$ and $mGlu_6$ indicated by red bars above, while non-conserved amino acids are highlighted in yellow.

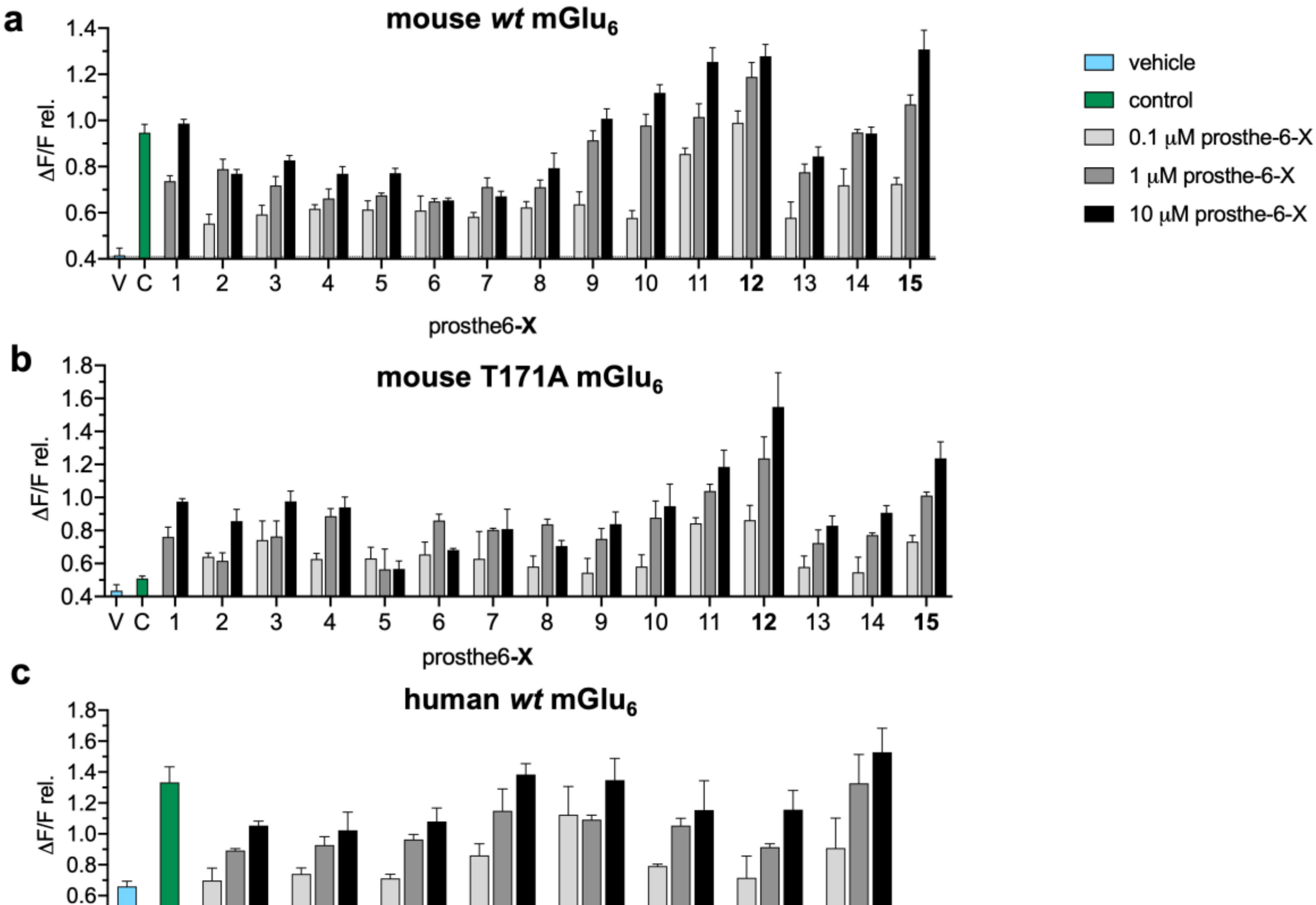

Figure S8. *In vitro* screening of the novel photoswitches in Human embryonic kidney (HEK) cells expressing mouse mouse wild-type (wt) or mutant T171A, and human *wt* metabotropic glutamate 6 ($mGlu_6$) receptors. The fluorescence-based calcium mobilization assay was performed in HEK cells tsA201 overexpressing **(a)** mouse *wt*, **(b)** mouse mutant T171A, **(c)** human wt $mGlu_6$ receptor. Cells were co-transfected with $mGlu_6$ receptor, $G_{q/i}$[3] and GCaMP6s. The entire library of photoswitchable compounds was evaluated at three concentrations (0.1, 1 and 10 µM) in (a) mouse *wt* or (b) T171A mutant. The seven best performing compounds were tested in human $mGlu_6$ receptor. The vehicle is represented in light blue and served as negative control across all experimental conditions. L-2-amino-4-phosphonobutyric acid (L-AP4) is depicted in green and was used as positive control in experiments involving both human and mouse wt $mGlu_6$ receptors, while serving as a negative control in experiments with the T171A mutant $mGlu_6$. Data are relative to the maximal response evoked by prosthe6-**1** (10 µM). Results are shown as the mean of at least four independent experiments (with n=3 replicates for each) ± SEM.

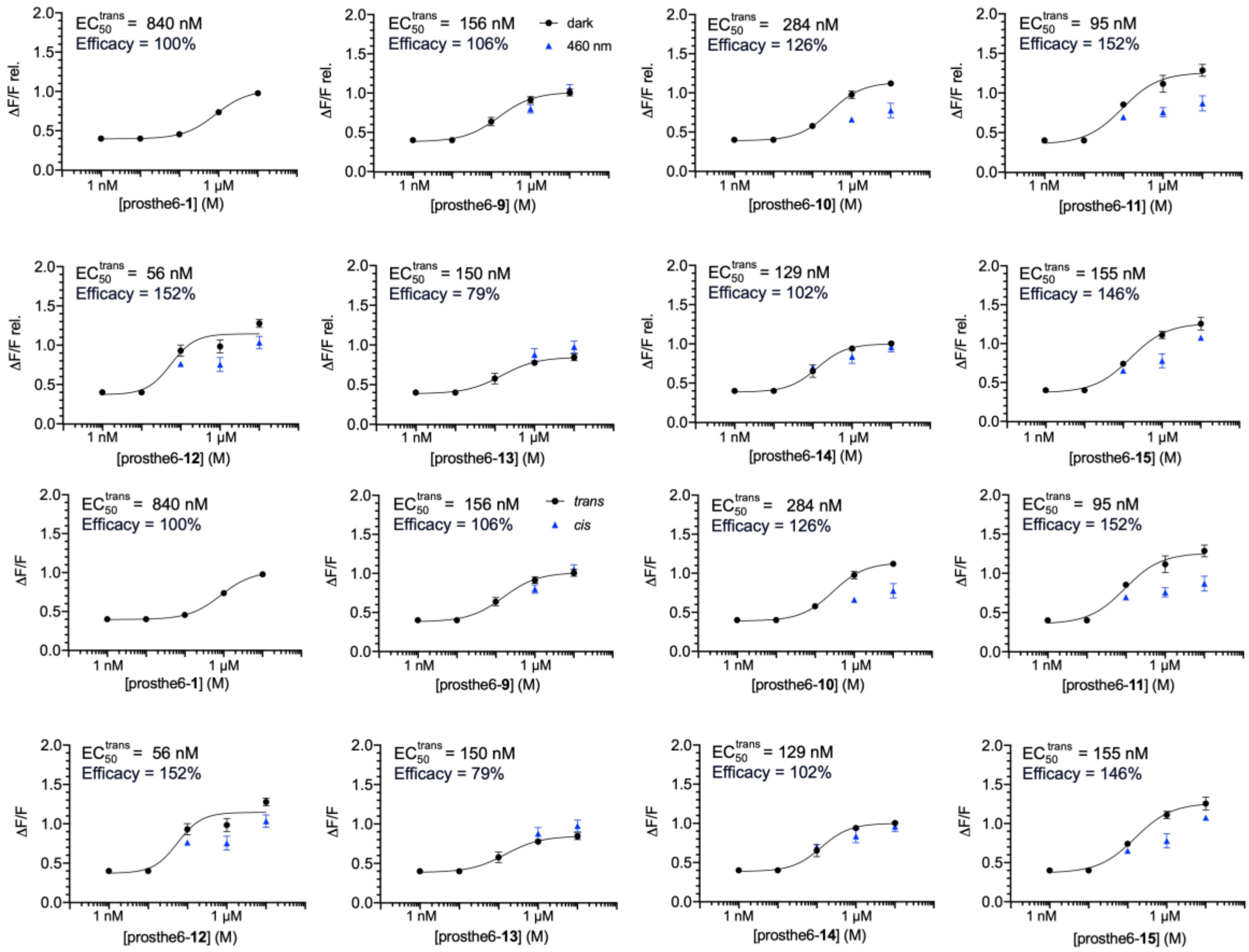

Figure S9. Dose response relationship in mouse wild-type (*wt*) metabotropic glutamate 6 ($mGlu_6$) receptor. The concentration-dependent curve data were generated from GraphPad using a sigmoidal dose-response curve model with variable slope. These data were plotted as ΔF/F and relative to the response elicited by prosthe6-**1** **(**10 µM). The potency and efficacy of prosthe6 were determined by calculating the compound concentration required to produce 50 % of receptor activation (EC_{50}) and the maximum response relative to that of prosthe6-**1**. The concentration-response curves (CRCs) of the *cis* isomers are represented with blue triangles for mouse *wt* $mGlu_6$, confirming a rightward shift of the plot upon illumination. Results are shown as the mean of at least three independent experiments (with n=3 replicates for each) ± SEM.

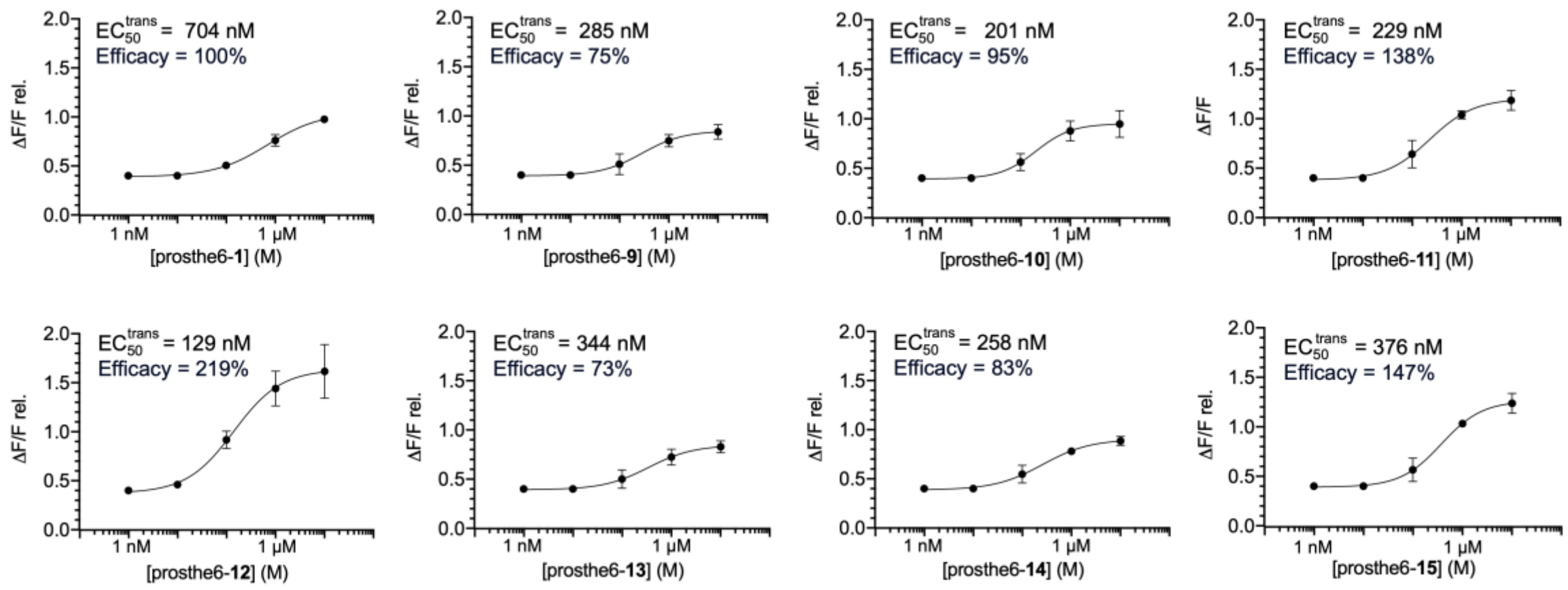

Figure S10. Dose response relationship in mouse mutant T171A metabotropic glutamate 6 ($mGlu_6$) receptor. The concentration-dependent curve data were generated from GraphPad using a sigmoidal dose-response curve model with variable slope. These data were plotted as ΔF/F and relative to the maximal response evoked by prosthe6-**1** (10 µM). The potency and efficacy of prosthe6 were determined by calculating the compound concentration required to produce 50 % of activation (EC_{50}) and the maximum response relative to that of prosthe6-**1**. Results shown as the mean of at least 3 independent experiments (with n=3 replicates each) ± SEM.

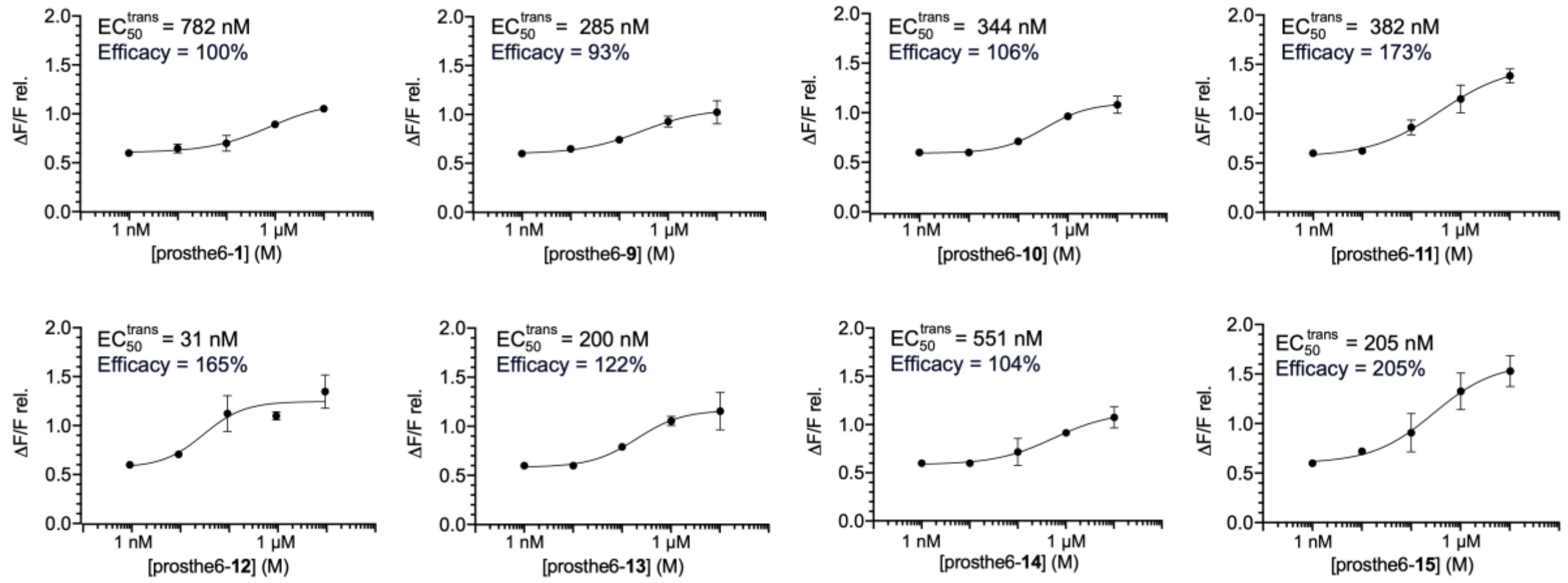

Figure S11. Dose response relationship in human wild-type (*wt*) metabotropic glutamate 6 ($mGlu_6$) receptor. The concentration-dependent curve data were generated from GraphPad using a sigmoidal dose-response curve model with variable slope. These data were plotted as ΔF/F and relative to the response elicited by prosthe6-**1** (10 µM). The potency and efficacy of prosthe6 were determined by calculating the compound concentration required to produce 50% of receptor activation (EC_{50}) and the maximum response relative to that of prosthe6-**1**. Results are shown as the mean of at least three independent experiments (with n=3 replicates for each) ± SEM.

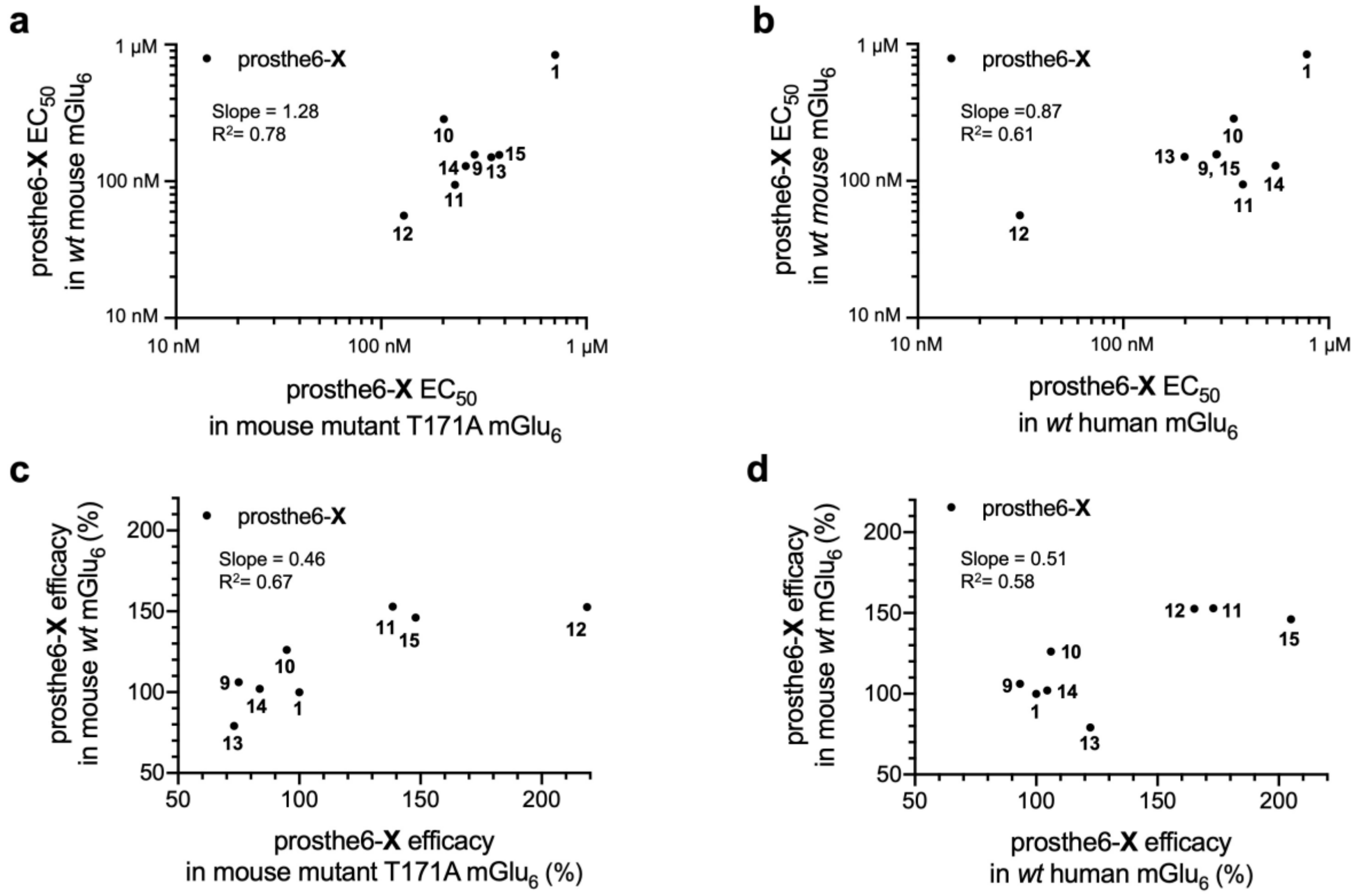

Figure S12. Correlations between EC_{50}/efficacy values in mouse wild-type (*wt*) *vs* mutant T171A and mouse *wt vs* human *wt* metabotropic glutamate 6 ($mGlu_6$). (a) The plot of prosthe6 compounds EC_{50} values in mouse *wt vs* mouse mutant T171A $mGlu_6$ revealed a high correlation, with R^2 and slope values of 0.78 and 1.28, respectively. **(b)** The plot of prosthe6 compounds EC_{50} values in mouse *wt vs* human *wt* $mGlu_6$ receptors showed more variability in human receptor potencies (30-550 nM) than in mouse (60-300 nM) resulting in a lower correlation (R^2 = 0.61, slope 0.87). (**c**) The plot of prosthe6 compounds efficacy values in mouse *wt vs* mouse mutant T171A $mGlu_6$ showed a weaker correlation (R^2=0.67, slope = 0.46). (**d**) The plot of prosthe6 compounds efficacy values in mouse *wt vs* human *wt* indicated a low correlation (R^2=0.58, slope =0.51).

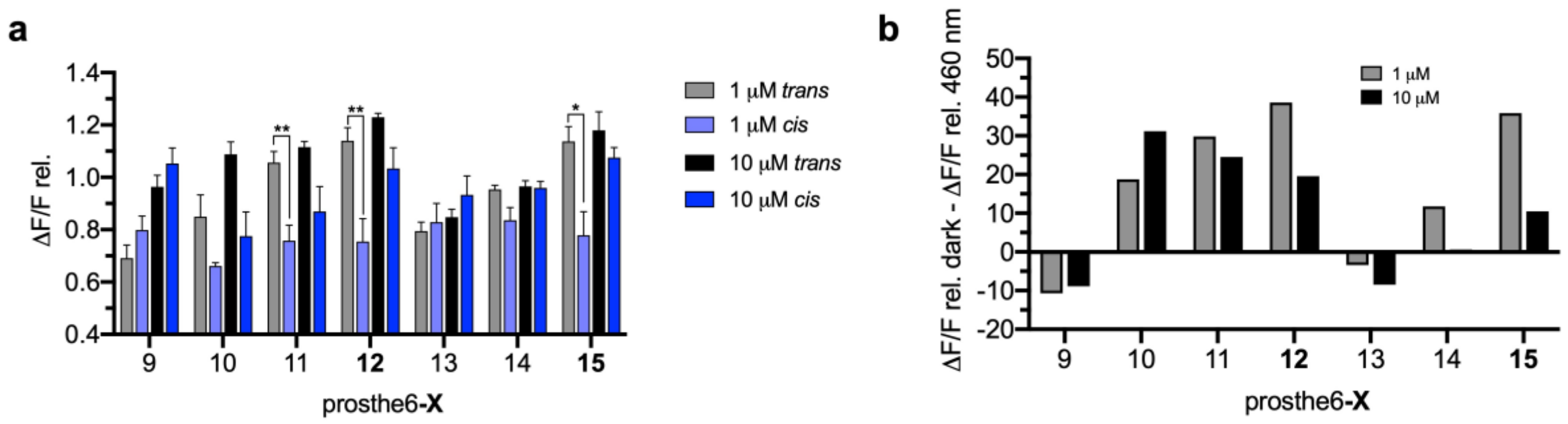

Figure S13. Prosthe6 compounds allow reversible photoswitching of metabotropic glutamate 6 ($mGlu_6$) receptor activity. (a) In mouse wild-type (*wt*) $mGlu_6$, we tested both the *trans* and *cis* forms of each compound (dark-relaxed and pre-irradiated for 2 min with blue light, respectively) at two concentrations (1 and 10 µM). The results illustrate the difference in response amplitude between *trans* and *cis* isoforms, indicating that prosthe6-**9** and -**13** are more active in *cis*, while prosthe6-**10**, -**11**, -**12**, -**14**, and -**15** are more active in *trans*. Notably, prosthe6-**12** and -**15** exhibit the largest differences in the group. The data were analyzed by the unpaired Mann-Whitney test (*p*-value (**) < 0.01, *p*-value (*) < 0.05; GraphPad Prism 6). Error bars are ± SEM. **(b)** Histograms showing the differences in amplitude photoresponses between dark and illuminated compounds, ($\Delta F/F$ rel. $_{dark}$ – $\Delta F/F$ rel. $_{illum}$) of the 7 selected prosthe6 compounds in mouse *wt* $mGlu_6$. Data are expressed in % of response at the corresponding concentration (1 and 10 µM). Data are relative to the maximal response elicited by prosthe6-**1** (10 µM). Positive values indicate higher efficacy in *trans*. Results are shown as the mean of at least four independent experiments (with n=3 replicates for each) ± SEM.

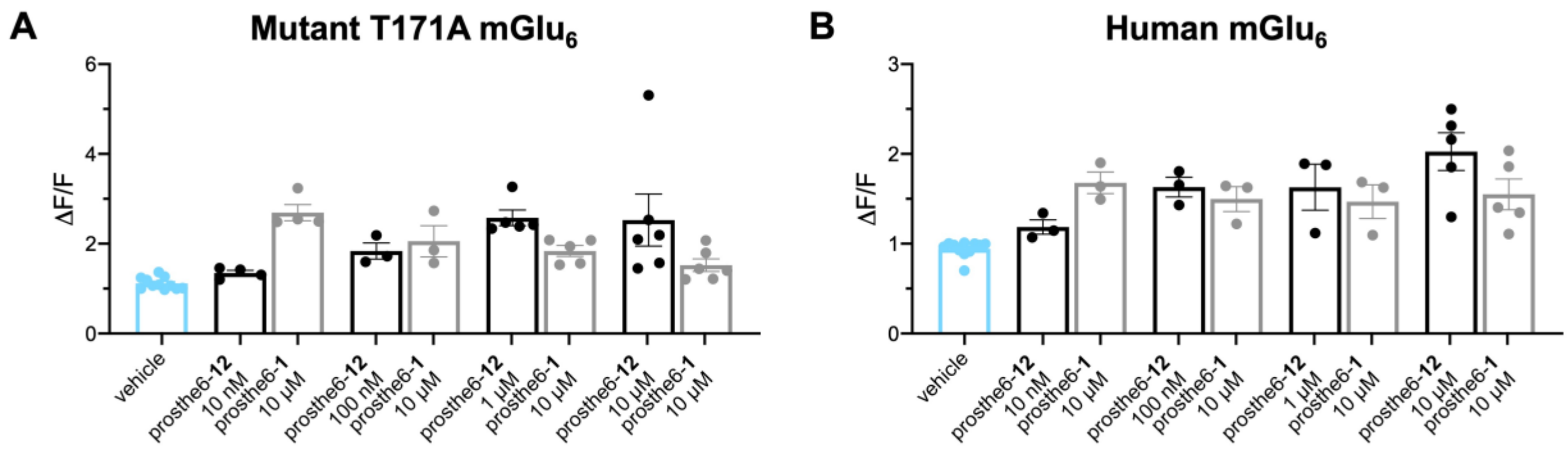

Figure S14. Two representative examples of ΔF/F dataset (not relative to prosthe6-1) for prosthe6-12 on mouse T171A mutant and human $mGlu_6$ receptors. (**a**) ΔF/F calcium responses measured in cells expressing the T171A mutant $mGlu_6$ receptor following application of prosthe6-**12** at the indicated concentrations (10 nM, 100 nM, 1 µM, and 10 µM). (**b**) ΔF/F responses recorded in cells expressing human wild-type (wt) $mGlu_6$ under the same treatment conditions. Bars represent mean ± SEM, and individual data points correspond to independent replicates. Prosthe6-**12** produced concentration-dependent modulation of receptor activity in both the mouse T171A mutant and human wt $mGlu_6$.

Plasmid construction

The mouse mutant (T171A) $mGlu_6$ receptor was created and had a point mutation (*i.e.*, a threonine to alanine exchange at position 171) in the "Venus flytrap" domain (VFT), resulting in a receptor that is insensitive to its orthosteric ligands. This site-directed mutagenesis was accomplished through a PCR-driven overlap extension by using the KOD Hot Start Master Mix kit (Sigma-Aldrich). The final product (around 800 bp) containing the mutation of interest was loaded on an agarose gel, purified by a gel extraction kit (Qiagen) and inserted in an expression vector (PGEM-T Easy Vector systems; Promega) to transform bacteria (One Shot™ MAX Efficiency™ DH5α-T1^R; ThermoFisher Scientific). Subsequently, the DNA from positively screened colonies was sequenced to verify the correctly established mutation. Then the insert and the vector were digested with the EcoRI (New England Biolabs), a restriction

enzyme, whereas the digested vector backbone was treated with the Antarctic phosphatase (New England Biolabs) prior to ligation, preventing the two ends of the vector to re-circularize. The cut insert was ligated into the vector and the complete insert + vector construct was transformed into the DH5α bacteria. Successful creation of the plasmid was screened through mini-prepping (#740588.50, NucleoSpin Plasmid, Mini kit for plasmid DNA) and Sanger sequencing of random colonies.

Fluorescence-based plate reader assay

Human embryonic kidney tsA201 (HEK tsA201, American Type Culture Collection; ATCC) cells were maintained in Dulbecco's Modified Eagle's Medium/Nutrient Mixture F-12 Ham (DMEM/F12 1:1, Life Technologies) supplemented with 10 % fetal bovine serum (FBS, Life Technologies), and 1 % antibiotics (Penicillin/Streptomycin; Sigma-Aldrich) in a controlled environment (37°C, 98 % humidity and 5 % CO_2). Mouse wild-type (wt), mouse mutant (T171A) or human wt $mGlu_6$ were transiently co-transfected with $G_{q/i}$ and the calcium indicator GCaMP6s (ratio 1:0.25:0.6) through X-tremeGENE 9 DNA Transfection Reagent (Roche Applied Science) following the manufacturer's instructions. The mouse $mGlu_6$ receptor plasmid was a kind gift from Prof. Takahisa Furukawa (Institute for Protein Research, Osaka University, Osaka, Japan). Prior to the experiment, co-transfected HEK tsA201 cells with *wt* $mGlu_6$ receptor were pre-incubated for 30 min with DMEM GlutaMAX (lacking L-glutamine) (Thermo Fisher Scientific Inc.).

In general, the culture medium was aspirated and 100 µL of recording solution was added to each well. The bath solution used for single-cell intracellular calcium recordings contained 140 mM NaCl, 5.4 mM KCl, 1 mM MgCl2, 10 mM HEPES, 10 mM glucose, and 2 mM CaCl2 (pH 7.4). This solution does not contain glutamate suggesting that the compounds may not require glutamate to activate $mGlu_6$. Subsequently, the signal was measured by the TECAN infinite 200 microplate reader (Tecan Austria GmbH), which was controlled by i-Control software (Tecan Group Ltd., version 1.6). The microplate reader was set at 485 nm excitation wavelength and 535 nm emission wavelength, while applying empirically optimized parameters (temperature 37ºC; integration time 40 µs; number of flashes 18; gain 120; number of cycles 25). Next, the blank or background signal was measured (*i.e.*, baseline reading). Later, 20 µL were taken from each well and 20 µL of the assay buffer, containing the compound or the control, were transferred to the cell plate and the changes in fluorescence intensity were recorded for 120 s.

Positive and negative controls were included in the experiments. In cells overexpressing the human *wt* or mouse *wt* $mGlu_6$ receptor, L-2-amino-4-phosphonobutyric acid (L-AP4) allowed to verify successful transfections. In cell overexpressing the mutant T171A $mGlu_6$ receptor the compound prosthe6-**1** was used to verify successful transfections. In all experiments, prosthe6-**1** served as the reference compound to which the responses of all other drugs were compared. The vehicle (2 % DMSO) resulted in a minimal fluorescence intensity to ensure that does not interfere with the receptors, was used as negative controls. All the experiments were performed under dark conditions to keep the compounds in their *trans* conformation. Test compounds were dissolved in DMSO (Sigma-Aldrich) and diluted with the assay buffer to a 2 % concentration of DMSO in each well. Compounds were tested at 0.1 µM, 1 µM and 10 µM. Seven compounds were additionally assessed at 0.01 µM. To study the effect of the compounds in the *cis* conformation, they were illuminated with light with 460 nm wavelength for 2 min, and then were added to the cells as soon as possible because are fast-relaxing molecules.

All data obtained with this assay were analyzed by using Excel (Microsoft, version 16.62) and GraphPad Prism (GraphPad Software, version 9). First, the raw data obtained by the Tecan were grouped in a single Excel sheet, because for each run the microplate reader produces a different sheet. The Relative Fluorescence (RF) was calculated through the ratio between the peak fluorescence and baseline for each well. Subsequently, the data were expressed relative to the maximal response elicited by prosthe6-**1** (10 µM). Dose-response curves were obtained with GraphPad Prism using a sigmoidal dose-response curve model with variable slope. Statistical differences were analyzed by one-way

ANOVA followed by Tukey's post-hoc test, whereas a value of *p*-value (*) < 0.05 was considered as significant. The data are expressed as the mean ± SEM for at least three independent experiments.

4. Toxicity and drug-likeness prediction of seven prosthe6 compounds.

Toxicity of seven best performing prosthe6 compounds, BENAQ and Foliglurax (Figures S15 and S16) was predicted *in silico* using *ProTox 3.0*, which is a web server for the prediction of several toxicological endpoints based on chemical similarities of the input molecules to known toxic compounds whose *in vitro* and *in vivo* real data is used to train a machine learning model.

Among the output values obtained from this software, a toxicity class ranking from 1 to 6 is provided according to the globally harmonized system of classification of labelling of chemicals (GHS), based on the predicted Lethal Dose (LD50), stratifying the output as follows:

- Class I: fatal if swallowed (LD50 ≤ 5 mg/kg)
- Class II: fatal if swallowed (5 mg/kg < LD50 ≤ 50 mg/kg)
- Class III: toxic if swallowed (50 mg/kg < LD50 ≤ 300 mg/kg)
- Class IV: harmful if swallowed (300 mg/kg < LD50 ≤ 2000 mg/kg)
- Class V: may be harmful if swallowed (2000 mg/kg < LD50 ≤ 5000 mg/kg)
- Class VI: non-toxic (LD50 > 5000 mg/kg)

This model is based on empirical data from acute oral toxicity tests in rodents using 38515 compounds for model training. It considers two-dimensional similarity of the input compound to others with known LD50, using molecular fingerprints and molecule fragments as descriptors. More information about the method and the different models used for all toxicological endpoints prediction can be found at their website in the *Model Info* section.[4]

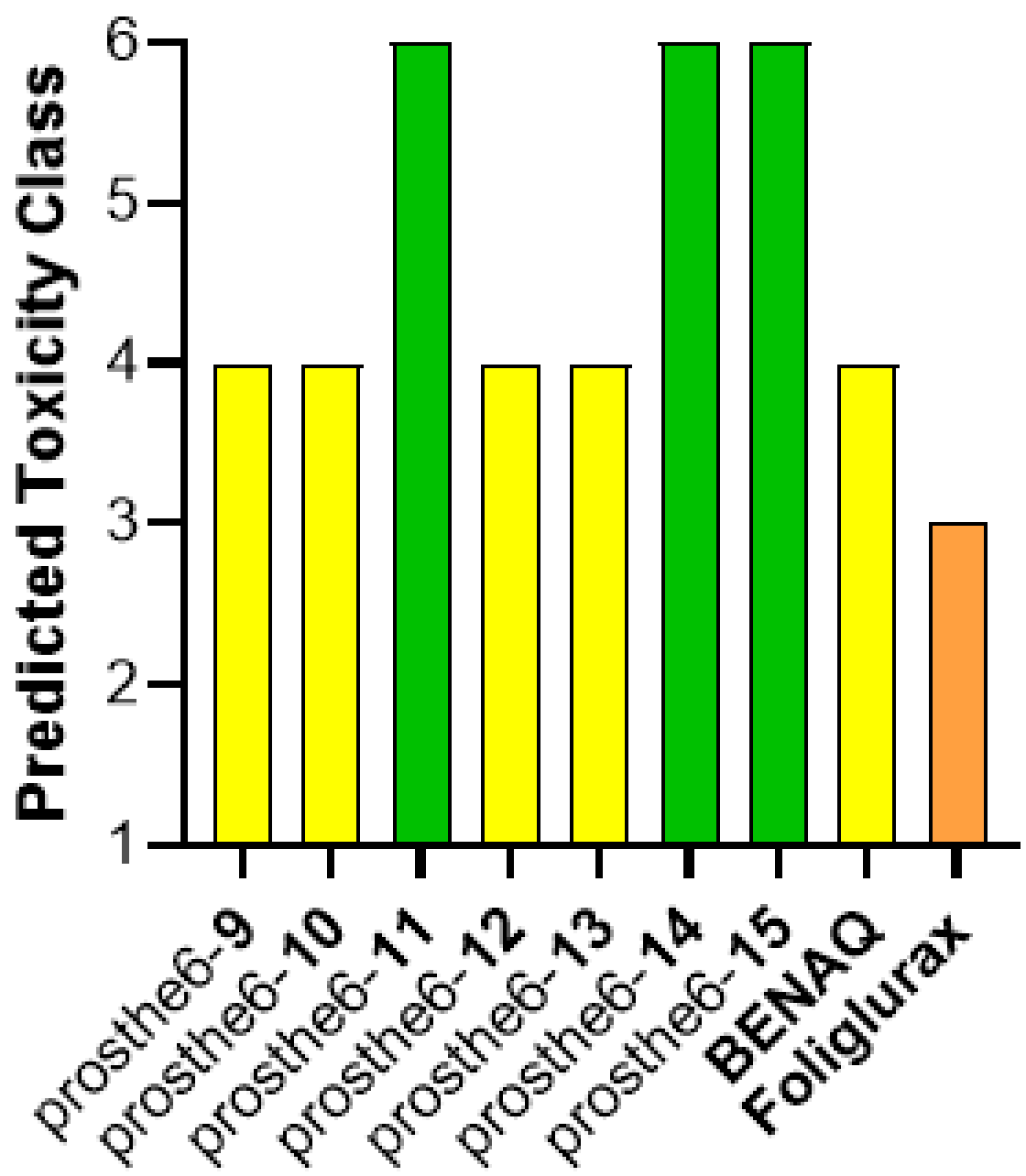

Figure S15. *In silico* oral toxicity prediction results for seven different prosthe6-X compounds, BENAQ and Foliglurax. These data were obtained from *ProTox 3.0*.

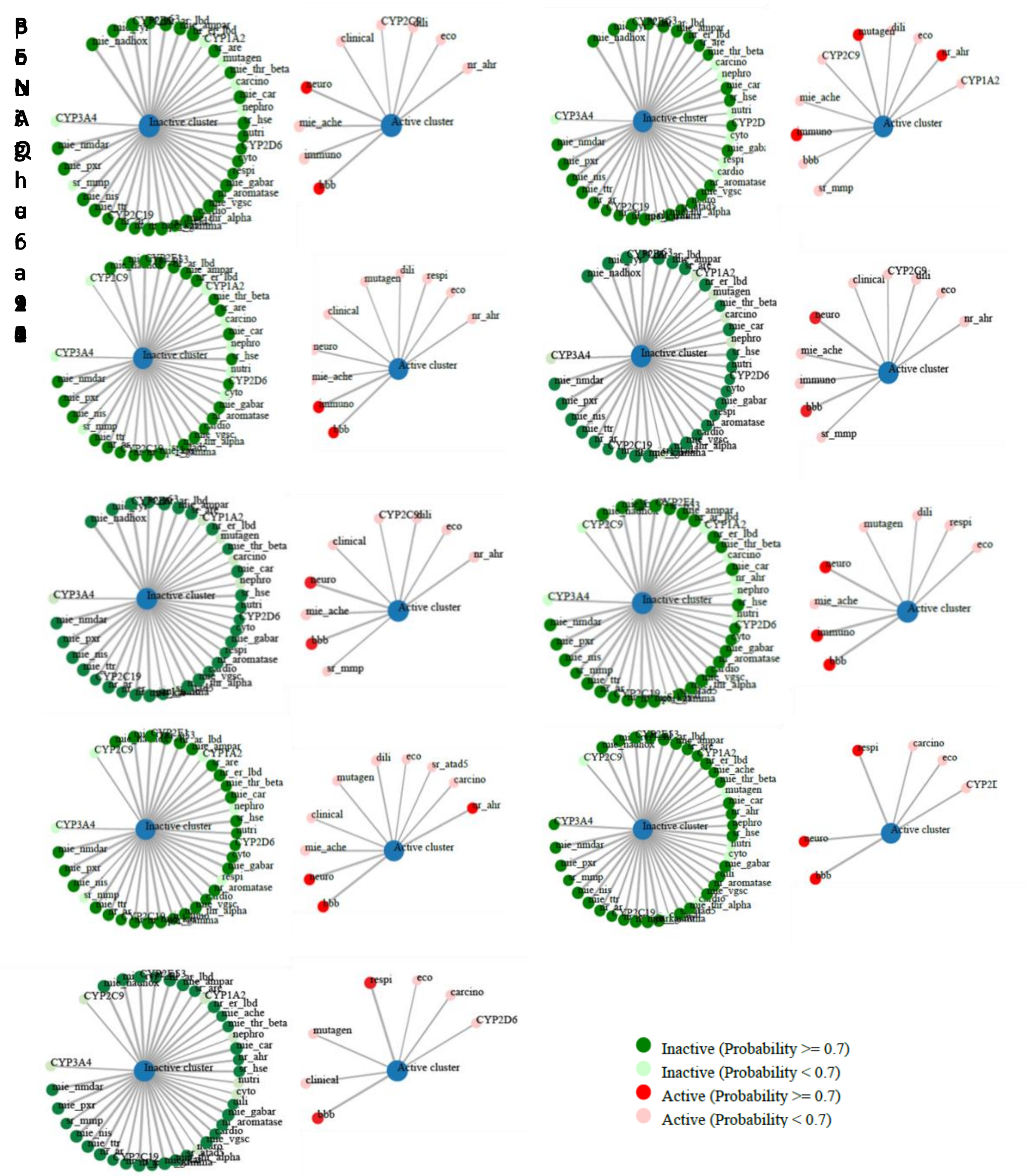

Figure S16. Toxicity network charts of seven different prosthe6 compounds, BENAQ and Foliglurax. Results obtained from *ProTox 3.0*. The chart shows the connection of each compound with different predicted activities, presenting in green or red the different toxicity endpoints for which it has been predicted to be inactive or active respectively. Confidence of the prediction is reflected in the intensity of the colour, as shown in the lower-right corner reference.

prosthe6-**X**	*Average Similarity*	*Prediction Accuracy*	*Predicted Toxicity Class*	*Predicted LD_{50} (mg/kg)*	*In Vitro EC_{50} in human $mGlu_6$ (Figure S11)*
**9**	47 %	54 %	4	2000	908 nM
**10**	47 %	54 %	4	810	344 nM
**11**	53 %	67 %	6	10000	300 nM
**12**	47 %	54 %	4	2000	31 nM
**13**	45 %	54 %	4	750	215 nM
**14**	49 %	54 %	6	10000	590 nM
**15**	51 %	67 %	6	10000	782 nM
**BENAQ**	65 %	68 %	4	560	9500 nM#

Table S2. Summary of the main features of acute oral toxicity prediction for all prosthe6-X and BENAQ. These values were calculated using *ProTox 3.0*. Average two-dimensional similarity of the input compound to compounds with known LD50, as well as the hit rates obtained in a cross-validation study determine the prediction accuracy. (#) For BENAQ, EC_{50} is extracted from *ex vivo* retinal photosensitization dose response curves and was not tested for metabotropic glutamate 6 ($mGlu_6$) activity.[5]

Drug-likeness calculations

Drug likeness was calculated using ChemDraw version 20.0.0.41 (Perkin Elmer Informatics) to generate two-dimensional structures of 7 prosthe6-**X** and ChemDraw20 for Excel add-in (Microsoft, Version 2402) to calculate LogP, molecular weight, number of Hydrogen bond donors and acceptors. To determine if a molecule was drug-like, Lipinski's rules of five[6] were followed (at least three out of the following four rules must be complied: molecular weight ≤ 500, Log P ≤ 5, Hydrogen bond donors ≤ 5 and Hydrogen bond acceptors ≤ 10).

	Lipinski's rules				
prosthe6-**X**	*MW*	*HB Acceptors*	*HB Donors*	*Log P*	*Drug-Like?*
**9**	397.82, yes	7, yes	1, yes	3.67, yes	yes
**10**	421.41, yes	7, yes	1, yes	3.91, yes	yes
**11**	405.46, yes	7, yes	1, yes	4.40, yes	yes
**12**	367.79, yes	6, yes	1, yes	3.46, yes	yes
**13**	436.68, yes	6, yes	1, yes	4.57, yes	yes
**14**	475.29, yes	6, yes	1, yes	0.00 (salt) , yes	yes
**15**	333.35, yes	6, yes	1, yes	2.90, yes	yes

Table S3. Calculated drug-likeness of seven prosthe6-X.

5. *In vivo* photopharmacological characterization in zebrafish larvae

Zebrafish housing

Zebrafish embryos (Tupfel long-fin strain) were purchased from the animal facility of the Barcelona Biomedical Research Park (PRBB) and raised in darkness for 7 days at 28 °C in UV filtered tap water in Petri dishes (daily cleaned and refilled). Animal development was checked every 24 h. Unhealthy or abnormal embryos and larvae were removed and euthanized in tricaine methanosulfonate 0.02 %. All experiments and procedures were conducted according to the European Directive 2010/63/EU.

Blinding Procedure

The animals underwent a noninvasive and highly specific blinding technique that induces photoreceptor apoptosis in the dorsal and central retina. The light-lesioning procedure was performed in a thermostatic and well-ventilated room equipped with small fans for air circulation and heat dissipation. Larvae at 5 days postfertilization were placed in a 35-mm petri dish containing 20 ml of UV filtered tap water, inserted in a closed mirrored chamber, and then exposed to 135.000 lux light emitted by a mercury lamp (model Olympus U-LH100HG) for 30 min. Temperature was monitored before and after the procedure.

Optokinetic reflex (OKR) assay

Tupfel long-fin strain zebrafish larvae aged 6-7 days post fertilization were used for OKR assays. Larvae were placed in a 35mm petri dish containing a droplet of approximately 100 µL of 6 % methylcellulose (Sigma Aldrich, M6385 Lot # SLBS9535) for immobilization, facing outwards with dorsal side up at a distance of 4.5 cm to the stimuli. OKR device was a customized equipment manufactured by Fundació Eduard Soler. It consists of a rotating conic carousel with interspersed mirrors and windows of 2 cm width, placed below a binocular microscope (OM99-ACRO 6.5X-45X Articulated Boom Stereoscope with Acrobat stand; Microscope LLC, Roanoke, VA) coupled to a cool white LED ring with variable intensity (80 LED Ring Light, OMANO) providing lighting for photoswitching video recording and binocular OKR stimuli generation. One-minute recordings were obtained by duplicate/triplicate per larvae and condition using a camera adapted to the microscope (Summit D3K2-5 5.0MP USB 3.0 High-Speed Digital Microscope Camera, Microscope LLC, Roanoke, VA) attached to a with confocal distances adjusted for maximum clarity of larvae's eyes and ToupView Image software (software version 4.8.16143.20191216, ToupTek Photonics, Hangzhou, China). When the carousel rotates clockwise at adjustable speed (6-60 revolutions per minute, rpm), white light reflected on the mirrors generates a moving pattern of highly contrasted light/dark stripes. Irradiance conditions were measured using a powermeter (PM101 - Power Meter Interface with USB, RS232, UART, and Analog Operation, Thorlabs GmbH) coupled to a sensor (S470C - Thermal Power Sensor Head, Volume Absorber, 0.25 - 10.6 µm, 100 µW - 5 W, Ø15 mm, Thorlabs GmbH) placed on the center of the OKR setup facing to the mirrors in the direction fishes are placed for the experiments, and processed using Optical Power Monitor Version 4.1.4204.742 (Thorlabs GmbH). Experiments were performed from 8.00 AM to 2.00 PM (UTC+01:00) and recordings were manually analyzed quantifying the number of saccades per minute, considering as saccade for quantification only a complete slow movement of the eye when tracking the rotating stimuli (monocular or binocular) followed by a fast movement in the opposite direction recovering eye initial position, rather than any fast eye movement. Explanatory recordings from a non-blinded larva and a blinded larva before and after prosthe6-**12** administration are shown in Supplementary movies M1 and M2.

Different rotational speeds of the carousel were tested with non-blinded larvae to set an optimal speed for the assay (Figure S16), which was set at 11 rpm for all experiments. For prosthe6-**X** efficacy assays and vehicle controls, recordings were performed before and one minute after the addition a 10 µL droplet of the compound on top of larvae's head (Figure S18). Considering the volume of the methylcellulose droplet, a 1/10 dilution could be considered for the compounds once applied, although due to the methylcellulose viscosity the actual dilution calculation would require further

investigation. For experiments involving administration of 1-Bz-APDC (Figure 4f and S20), recordings were obtained before addition of prosthe6-**12**, one-minute after addition of a 10 µL droplet of the photoswitch and five min after addition of a µL droplet of 1-Bz-APDC. For obtaining the traces represented on Figure 4c, a region of the larva eye was selected to measure the mean grey value which changes according to the eye movements employing Image J software. To assess the efficacy of the prosthe6-**X** the saccades/min after addition of the compound were normalized to the mean of saccades/min performed by non-blinded larvae at 11 rpm (12.0±0.6).

All data obtained with this assay were analyzed by using Excel (Microsoft, version 16.62) and GraphPad Prism (GraphPad Software, version 9).

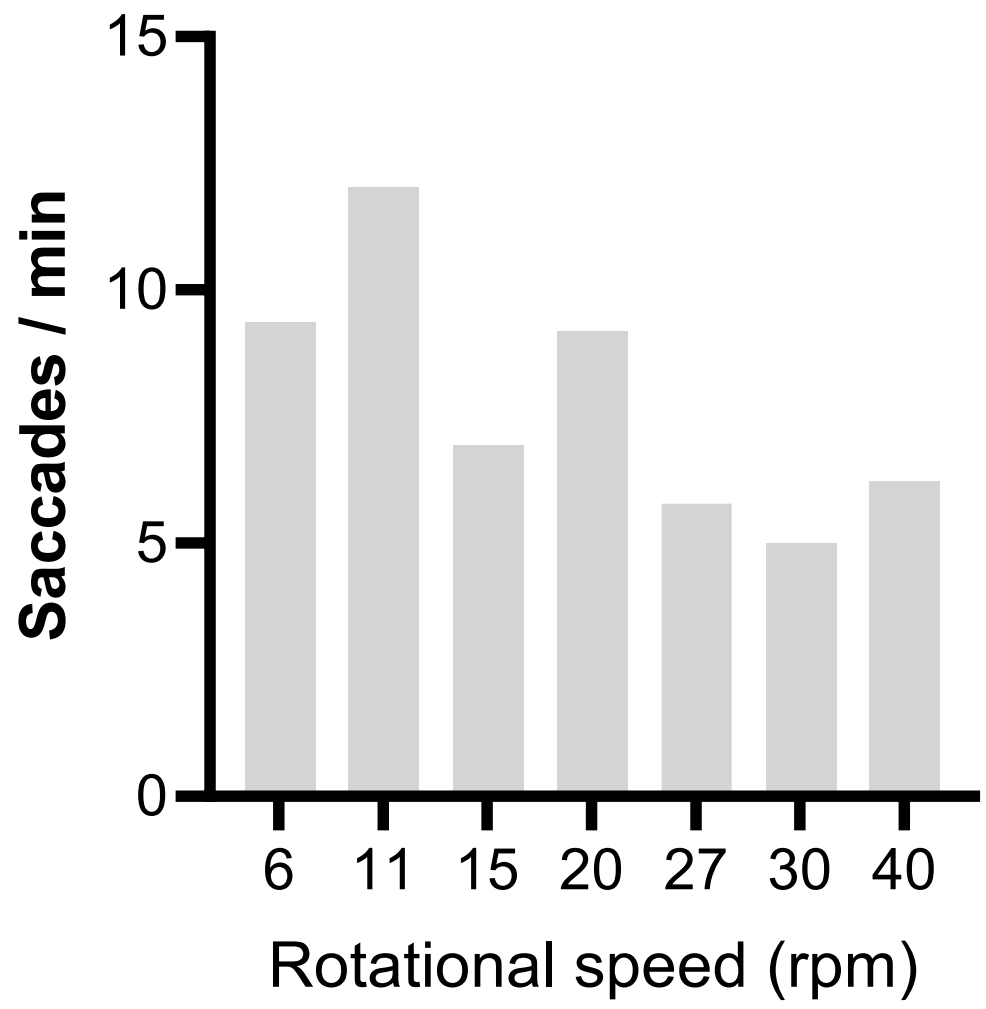

Figure S17. Mean of saccades per minute at different drum rotational speeds for non-blinded zebrafish larvae. Light irradiance = 3.26 mW·cm^{-2}.

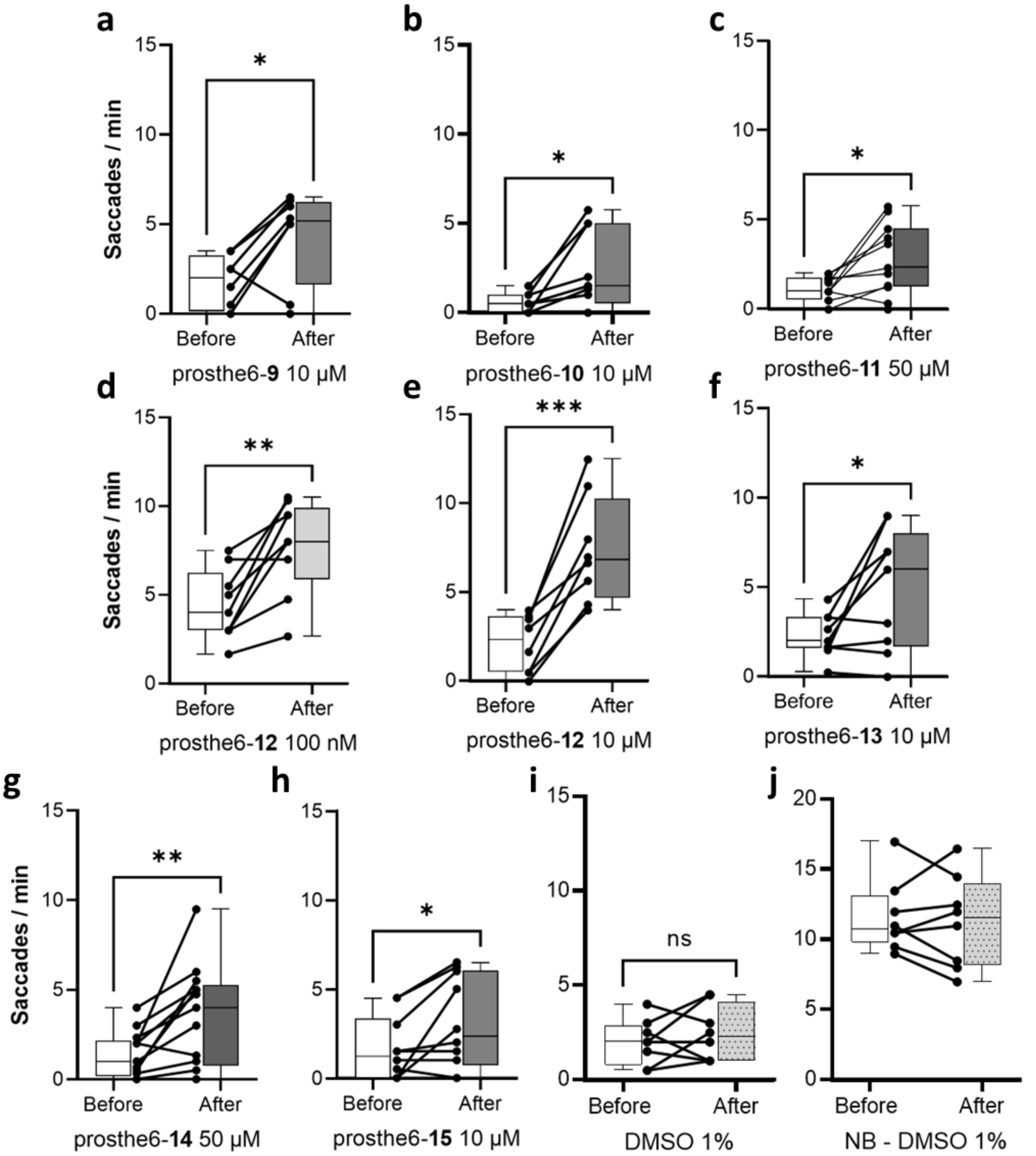

Figure S18. Optokinetic reflex (OKR) results of different concentrations of prosthe6-X in blinded larvae or vehicle (DMSO 1 % in blinded and non-blinded larvae. Saccades per minute before and after addition of a 10 µL droplet of different concentrations of prosthe6-**X** **(a-h)** or vehicle DMSO 1 % **(i)** on the head of the blinded zebrafish larvae. **(j)** Saccades per minute before and after addition of a 10 µL droplet of vehicle DMSO 1 % on the head of the non-blinded zebrafish (NB) larvae. Light irradiance = 3.26 mW·cm^{-2}. Results presented as mean ± SEM and individual responses. *p<0.05, **p<0.01, ***p<0.001, ns = not significant, paired t-test.

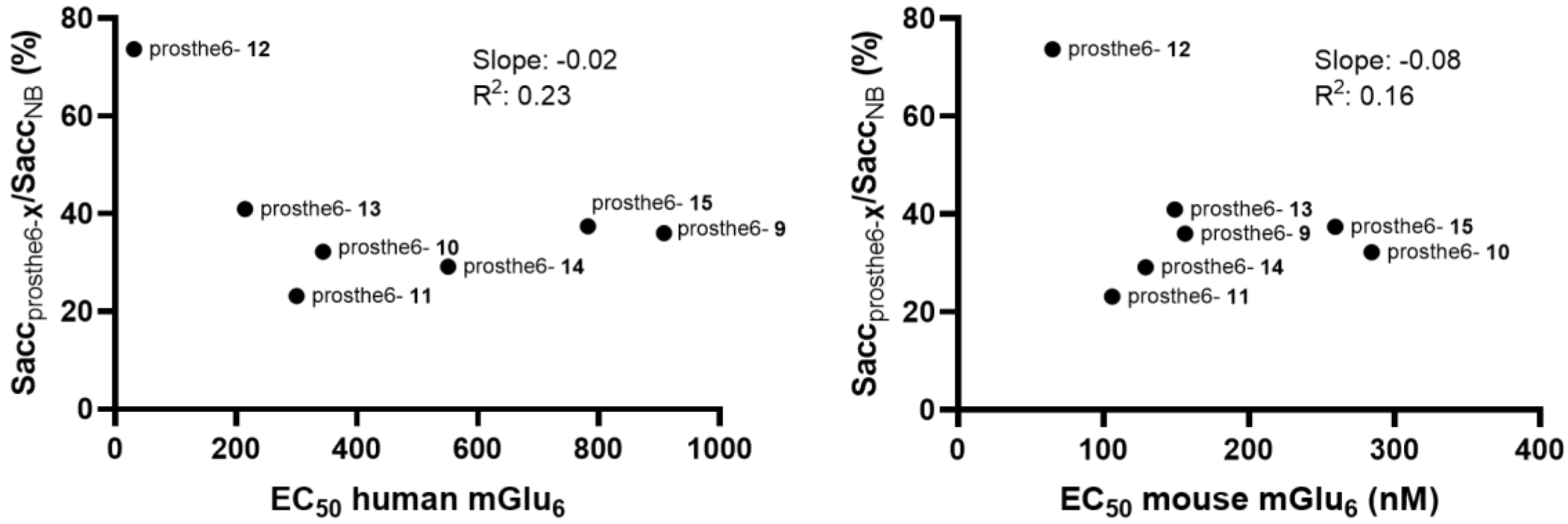

Figure S19. Correlation between Optokinetic reflex (OKR) best performance of prosthe6 and the *in vitro* EC$_{50}$ values for human and mouse wild-type metabotropic glutamate 6 (mGlu$_6$). OKR best performance of each prosthe6-**X** was normalized to response of non-blinded zebrafish larvae.

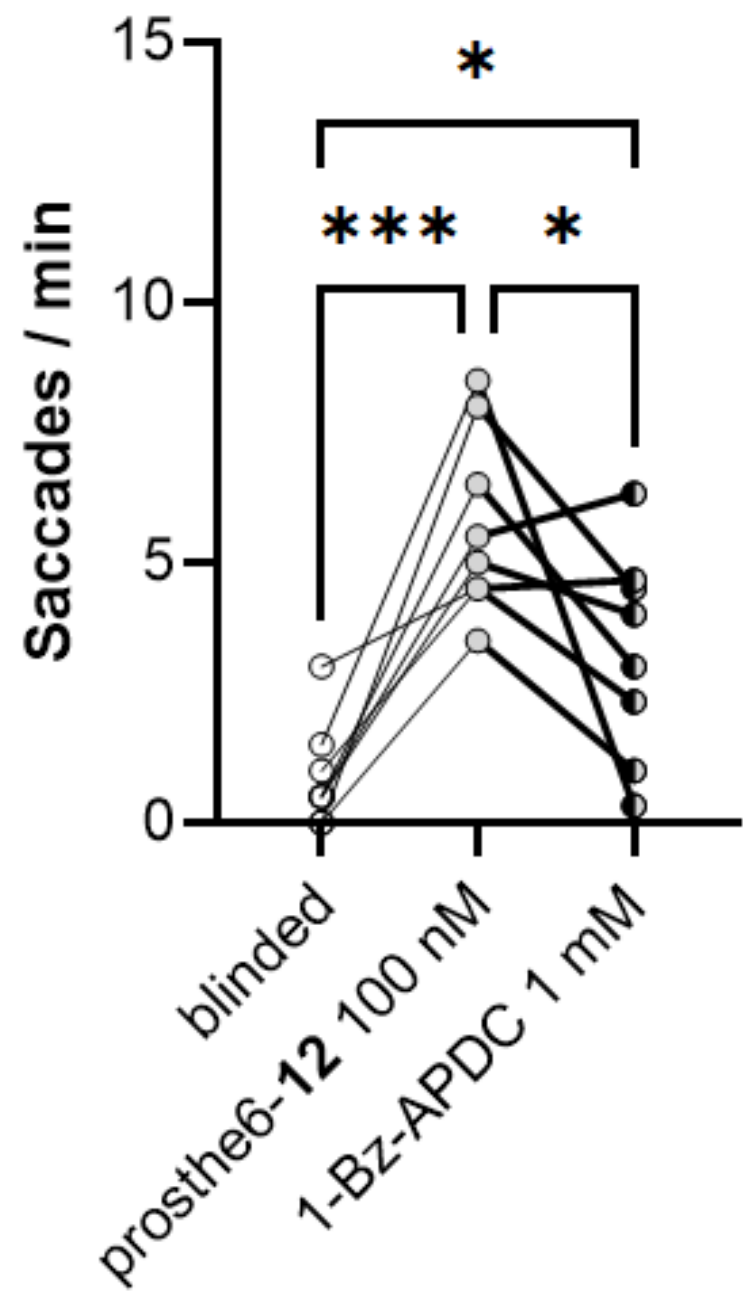

Figure S20. Target engagement experiment in zebrafish larvae. Saccades per minute before and after addition of a 10 µL droplet of prosthe6-**12** 100 nM on the head of the blinded zebrafish larvae, followed by the addition of a 10 µL droplet of (2R, 4R)-4-amino-1-benzylpyrrolidine-2,4-dicarboxylic acid (1-Bz-APDC) 1 mM. Light irradiance = 3.26 mW·cm^{-2}. The results correspond to Figure 4f, presented here as individual larvae responses. *p<0.05, **** p<0.0001 vs prosthe6-**12** 1 µM One Way ANOVA with Dunnett's multiple comparisons test.

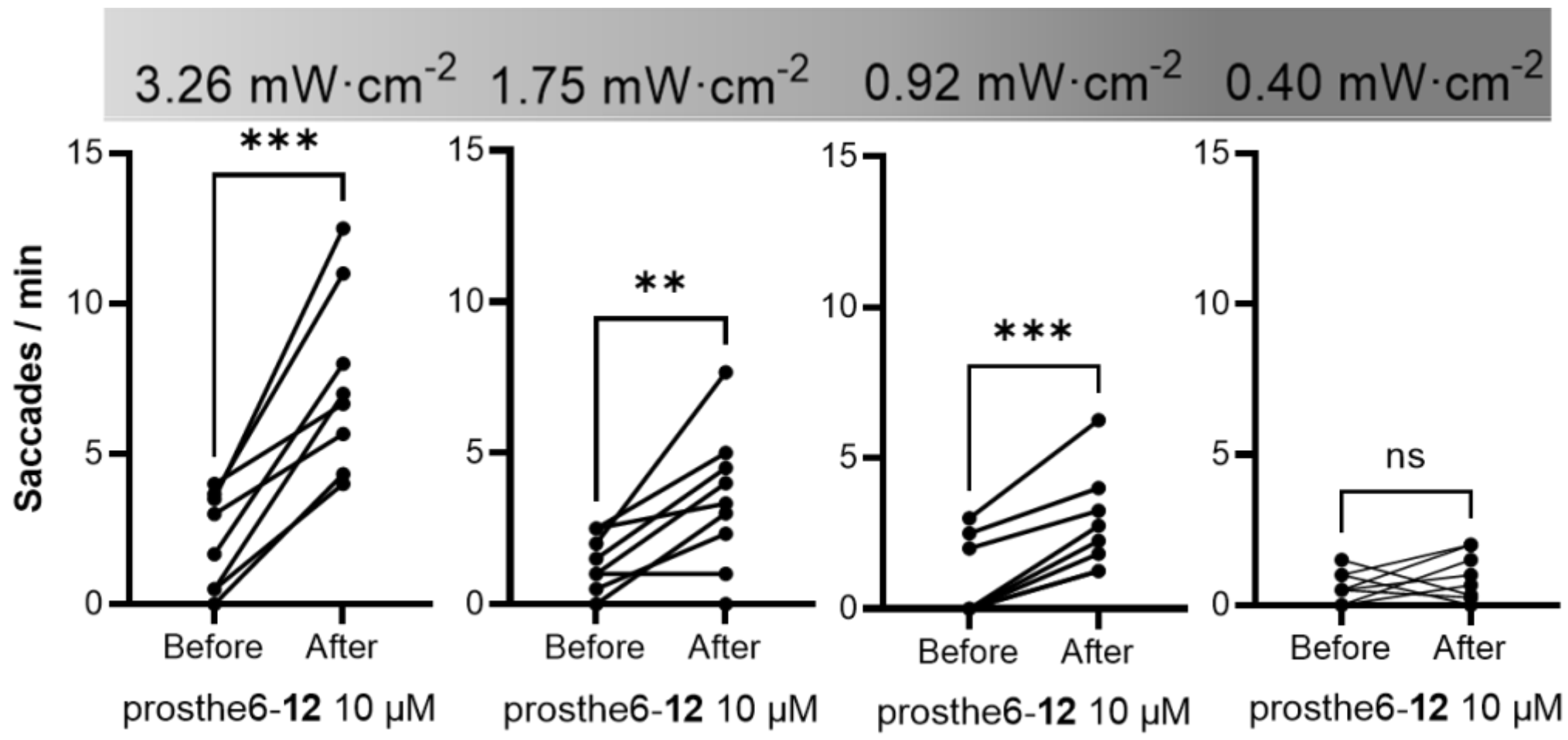

Figure S21. Optokinetic reflex (OKR) results with different concentration of prosthe6-12 under different conditions of light irradiance. Saccades per minute before and after addition of a 10 µL droplet of prosthe6-**12** 10 µM on the head of the blinded zebrafish larvae, under different conditions of light irradiance. The results correspond to Fig. 4h, presented here as individual larvae responses. ** p<0.01; *** p<0.001; ns = not significant; paired t-test.

6. *In vivo* toxicity in zebrafish larvae

Zebrafish Embryonic Toxicity Assay

For prosthe6-**12** and **BENAQ** toxicity assessment, we conducted a reported toxicity assay. Briefly, embryos were placed into flat-bottomed 96 well plate (Nunc™ MicroWell™ 96-Well, Nunclon Delta-Treated, Flat-Bottom Microplate, Thermo Scientific™) in a total volume of 270 µL, and then 30 µL of UV-filtered water, DMSO 1 %, prosthe6-**12** 1 µM in DMSO 1 % or **BENAQ** 100 µM in DMSO 1 % were added in the corresponding wells being therefore diluted ten times (final well volume = 300 µL). Exposition to the compounds was initiated at 6 h post fertilization (hpf) and embryos were revised at 24 and 120 hpf to count dead embryos and abnormalities under each condition. The observed abnormalities were overall short body, curved tail, and large yolk for the developmental stage. Remarkably, at 100 nM concentration which is already efficient for saccadic eye movements restoration, prosthe6-**12** did not show significant differences in deaths or abnormalities compared to the vehicle, whereas all **BENAQ** incubated embryos were dead at 120 hpf (Figure S22).

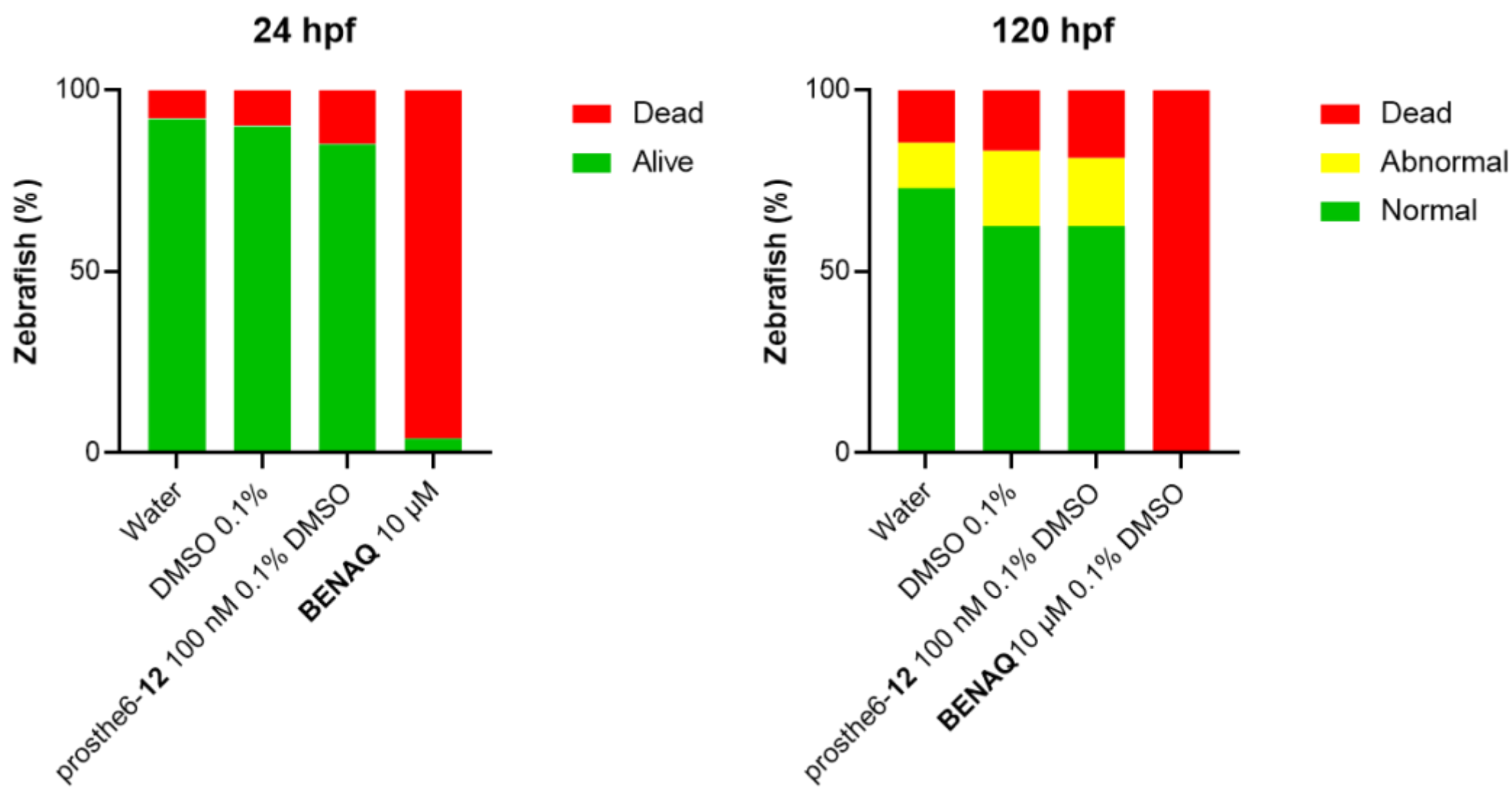

Figure S22. Zebrafish embryonic toxicity assay results for prosthe6-12 and BENAQ. The concentrations used for prosthe6-**12** and BENAQ were 100 nM and 10 µM, respectively.

7. In vitro pharmacology of prosthe6-12 and 15

An *in vitro* pharmacology assay was outsourced to Eurofins Cerep (France) to test prosthe6-**12** and **15** against a panel of 87 relevant targets. Percentage of inhibition of the binding of the natural target, enzyme activity or uptake in case of transporters is reported below for prosthe6-**12** (3 µM) and prosthe6-**15** (10 µM). In each experiment and if applicable, the respective reference compound was tested concurrently with both compounds, and the data were compared with historical values determined at Eurofins. The experiment was accepted in accordance with Eurofins validation Standard Operating Procedure.

Results showing an inhibition (or stimulation for assays run in basal conditions) higher than 50 % are considered to represent significant effects of the test compounds. 50 % is the most common cut-off value for further investigation (determination of IC_{50} or EC_{50} values from concentration-response curves) that we would recommend. Results showing an inhibition (or stimulation) between 25 % and 50 % are indicative of weak to moderate effects (in most assays, they should be confirmed by further testing as they are within a range where more inter-experimental variability can occur). Results showing an inhibition (or stimulation) lower than 25 % are not considered significant and mostly attributable to variability of the signal around the control level. Low to moderate negative values have no real meaning and are attributable to variability of the signal around the control level. High negative values (≥ 50 %) that are sometimes obtained with high concentrations of test compounds are generally attributable to nonspecific effects of the test compounds in the assays. On rare occasion they could suggest an allosteric effect of the test compound.

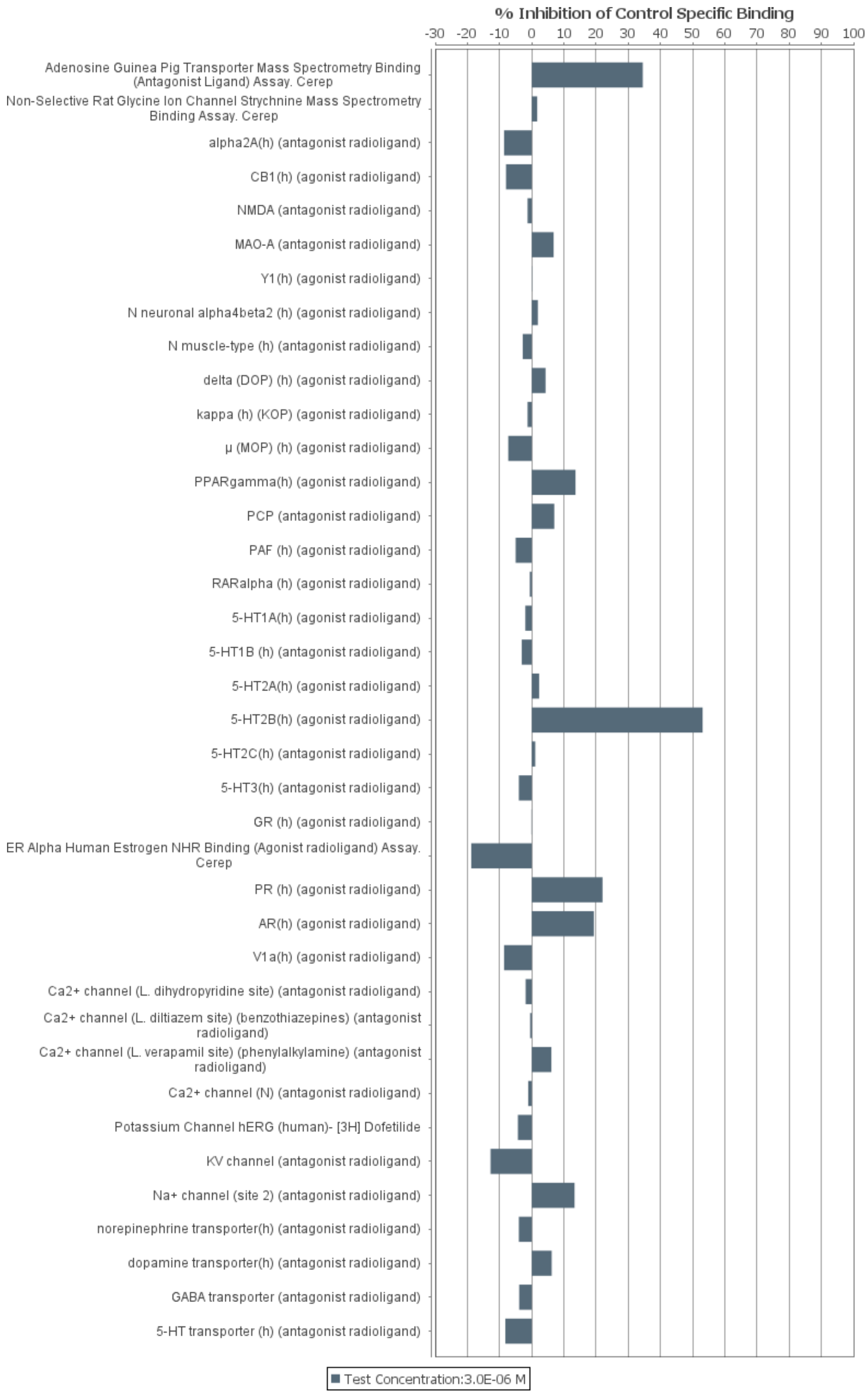

Figure S23*. In vitro* pharmacology results for prosthe6-12. Results showing an inhibition of 50 % or greater are considered to represent a significant effect of the compound.

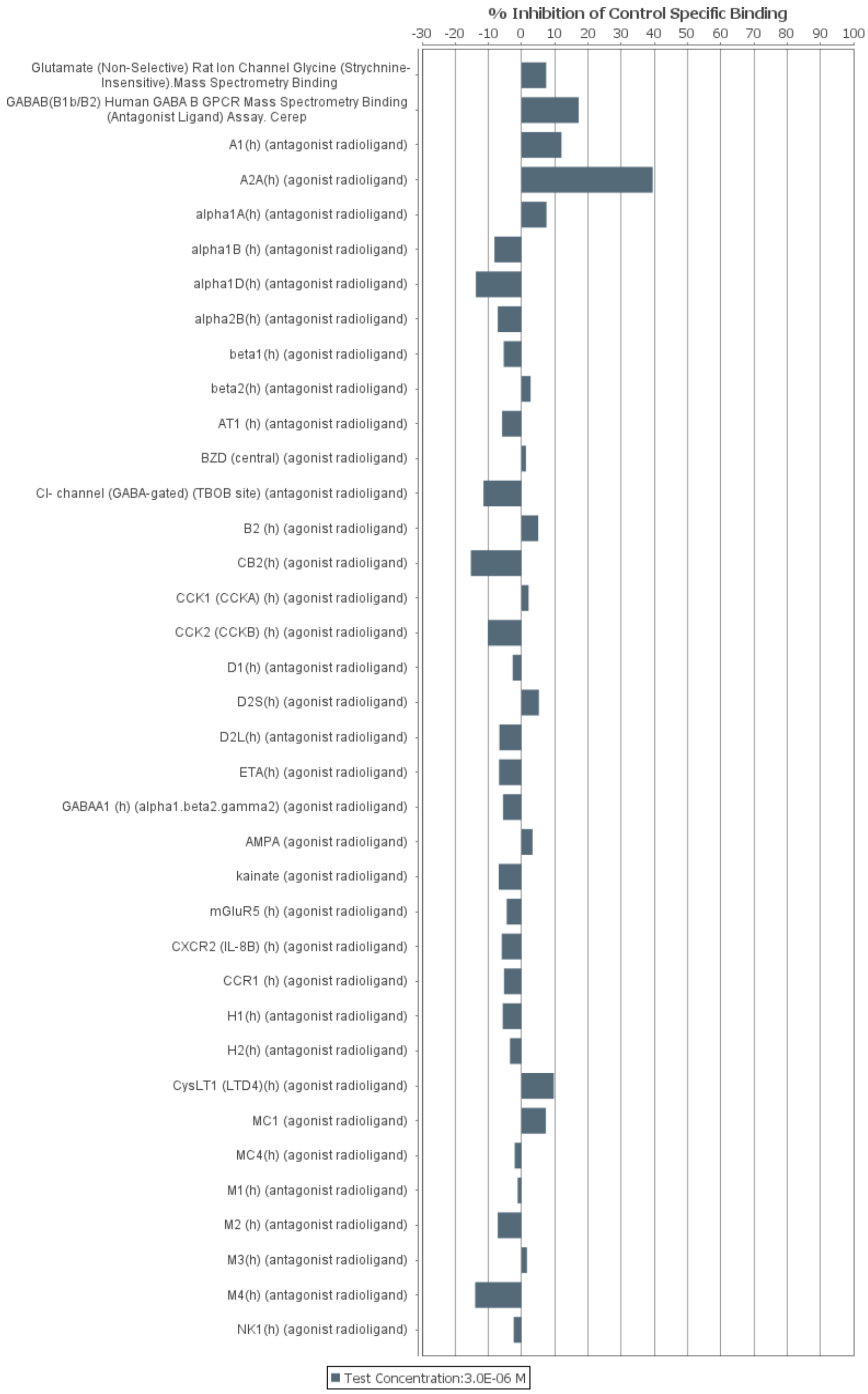

Figure S23 (cont.). *In vitro* pharmacology results for prosthe6-12. Results showing an inhibition of 50 % or greater are considered to represent a significant effect of the compound.

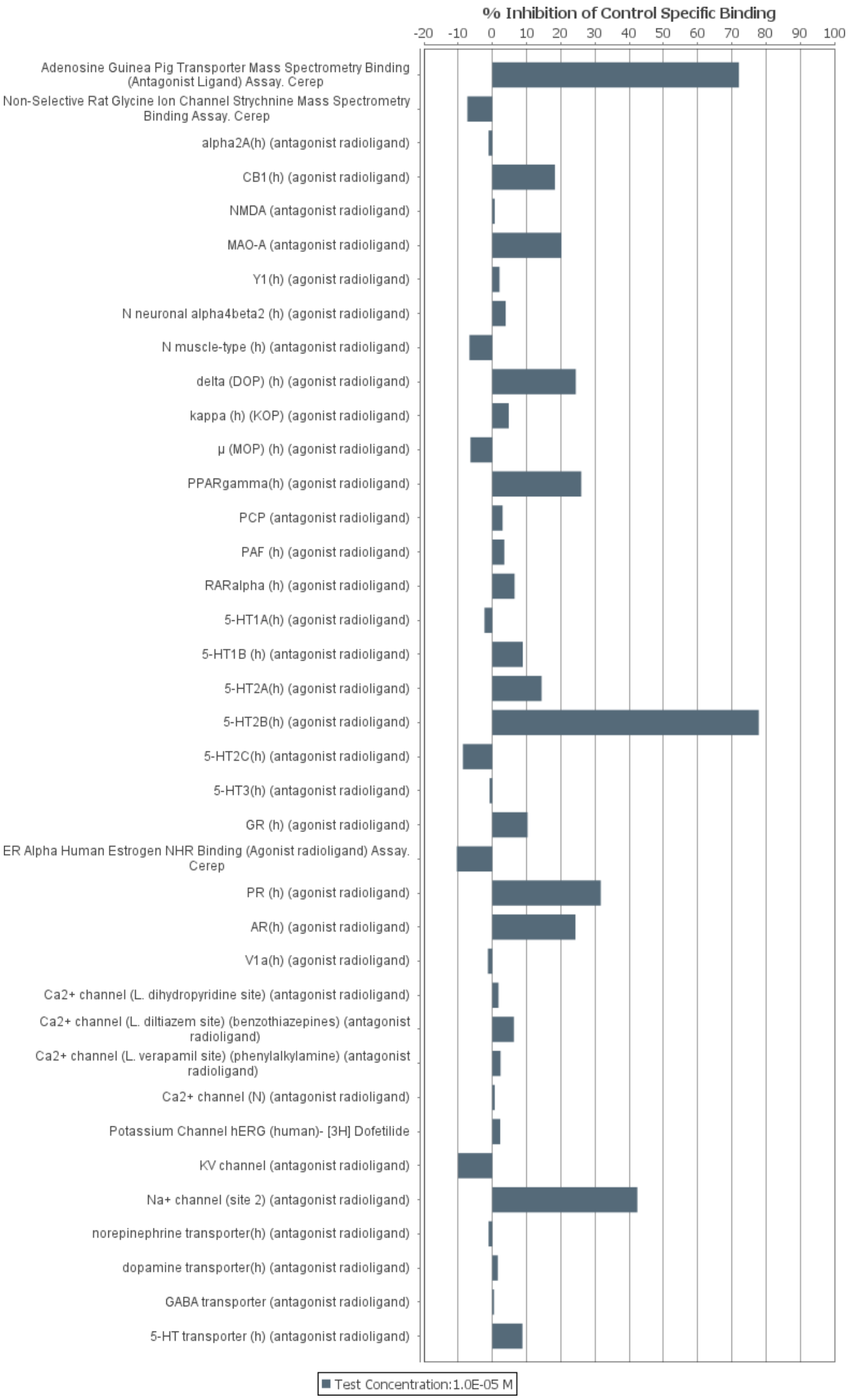

Figure S24. *In vitro* pharmacology results for prosthe6-15. Results showing an inhibition of 50 % or greater are considered to represent a significant effect of the compound.

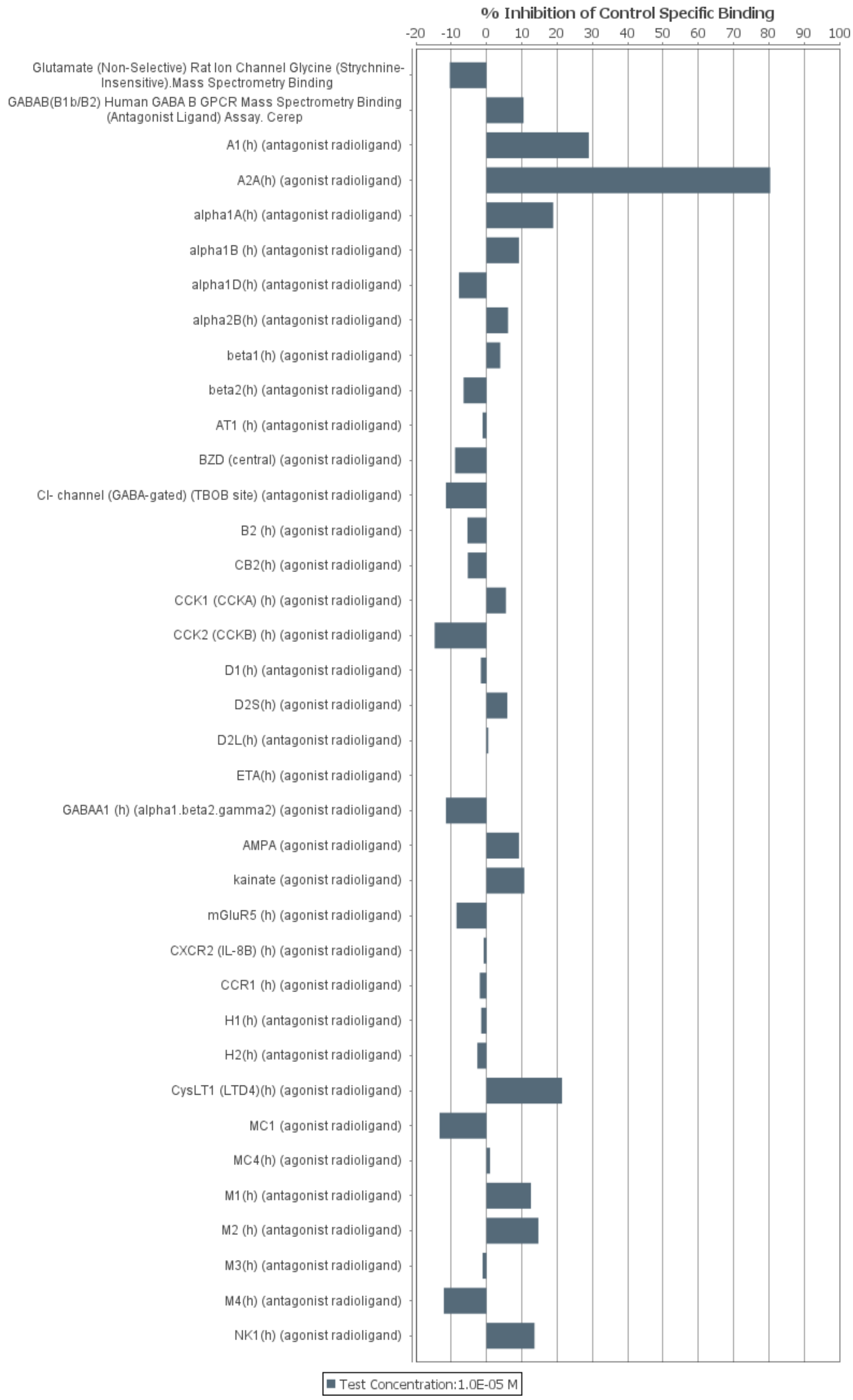

Figure S24 (cont.). ***In vitro*** **pharmacology results for prosthe6-15**. Results showing an inhibition of 50 % or greater are considered to represent a significant effect of the compound.

8. Light driven behavior in mouse models of macular degeneration (GA) and retinitis pigmentosa (RP)

Animals

<u>Opn4</u>$^{-/-}$**:** Knockout (KO) animal model with a mutation in the *Opn4* gene, responsible for melanopsin synthesis. The intrinsically photosensitive ganglion cells (ipRGCs) or melanopsin cells lack melanopsin photopigment and are therefore unable to respond to visual stimuli. This animal model was created by Hattar and colleagues (2002) by replacing the *Opn4* gene with a *tau-LacZ* gene insert. The genetic background consisted of a mixture of C57BL/6J and 129/SvJ, resulting in the KO *Opn4*$^{-/-}$ *model*.[7] This animal model was provided to our group by Dr. R. Foster, Circadian and Visual Neuroscience Group Nuffield Lab of Ophthalmology, University of Oxford. In this study, experimental groups consisting of 9 animals were used, with the follow distribution by sexes:

Prosthe6-12 - Blue Intravitreal			Prosthe6-12 - Blue Topical			Prosthe6-12 - White Topical			Prosthe6-15 - White Topical		
n	Sex	Age ($NaIO_3$ test)	n	Sex	Age ($NaIO_3$ test)	n	Sex	Age ($NaIO_3$ test)	n	Sex	Age ($NaIO_3$ test)
b.1	F	61	b.10	F	64	d.1	M	68	d.10	F	59
b.2	F	61	b.11	F	64	d.2	M	68	d.11	F	59
b.3	F	61	b.12	F	64	d.3	M	68	d.12	F	59
b.4	F	61	b.13	F	64	d.4	M	68	d.13	F	59
b.5	F	61	b.14	F	64	d.5	F	62	d.14	F	59
b.6	M	65	b.15	F	64	d.6	F	62	d.15	F	59
b.7	F	61	b.16	F	69	d.7	F	62	d.16	F	59
b.8	M	61	b.17	F	69	d.8	F	62	d.17	F	59
b.9	M	65	b.18	M	69	d.9	F	62	d.18	F	59

Table S4. *Opn4*$^{-/-}$ animals distribution. The table shows the number, sex, and age of the animals (according to Figure 5).

Opn4$^{-/-}$ animals underwent a control test of the light/dark transition test prior to the induction of geographic atrophic damage. After control tests, a dose of 65 mg/kg of sodium iodate ($NaIO_3$) was administered by intraperitoneal injection with a 25 G needle to damage the PhRs. The animals were then returned to their cages and kept calm (monitored) for 28 days, when they were again subjected to the transition test.

Animals that received intravitreal injections were anaesthetised using vaporised isoflurane (Isoflo©, Zoetis S.L., Madrid, Spain). For this purpose, a TEC 3 isoflurane vaporiser (MSS International Ltd, Keighley, UK) was used. A constant flow of 0.4 L/min of O2 was maintained during the procedures. To induce anaesthesia, the animal was placed in an airtight box and vaporised with a flow of 5 % isoflurane. After induction, the isoflurane flow was reduced to 1 % for the duration of the procedure (see ***Intravitreal Injection*** below), and the animal was switched to a mask holder adapted for mice.

<u>Pde6b</u>$^{rd10/rd10}$ **× *<u>Opn4</u>***$^{-/-}$**:** a combination of two animal models that lead to the loss of all photosensitive elements of the retina. The RP *Pde6b*$^{rd10/rd10}$ animal model is very well-characterized. It presents a mutation at the C-terminal end of the phosphodiesterase 6b (*Pde6b*) gene, specifically. The resulting truncated subunit is not able to hydrolyse cGMP, so cGMP-dependent channels cannot close. The flow of cations into the cellular interior cannot be stopped and eventually cell death pathways in photoreceptors are activated. This leads to the total loss of photoreceptors within 60 days postnatally.[8] However, the inner retina will not be severely affected by the loss of the outermost structures, with bipolar, amacrine and ganglion cells remaining. This type of neurodegeneration is similar to that which occurs in some cases of RP in humans, which makes this animal model a great tool for study. The combination of this *Pde6b* mutation with the *Opn4* gene deletion makes it possible to generate an animal model of absolute blindness but maintaining the structure of the innermost layers of the retina. In this way, this animal will not be able to detect any type of visual stimulus.[9] In this study, an experimental group consisting of 10 animals (P95) were used, with a homogeneous distribution of sexes (5 females and 5 males).

Intravitreal Injection

1 µL of the compounds (1.8 % DMSO) were administered by intravitreal injection with a 10 µL syringe (Nanofil™, World Precision Instruments, Florida, USA) with a 34 G needle (Nanofil™, NF35BV-2, World Precision Instruments, Florida, USA). The animal, anaesthetised with isoflurane, was placed under the magnifying glass (Wild Heerbrugg, Leica, Switzerland) to proceed with the injection, which was performed in the nasal area of the eyeball, behind the sclero-corneal limbus. In all cases the injection was performed in the right eye of the animals.

Topical Application

For topical administration, the animals were anaesthetised using isoflurane, as discussed above. A 10 µl drop was administered on the ocular surface of both eyes. The animals were anaesthetised for 5 min, favouring maximum absorption of the compound. After topical administration, animals were allowed to rest 2.5 hours before proceeding with the light/dark transition test, ensuring that there was no bias due to anaesthesia.

Light/Dark Transition Test

With this test we can measure the light sensitivity of an animal. They are introduced in a wooden experimental set consisting of two compartments, each 28.5 cm high and 28.5 cm on each side, and connected by a small opening in the wall, and with different light conditions (light intensities in lx measured with A.W. Sperry – SLM-110, A.W. Sperry Instruments, U.S.A; irradiances in $mW \cdot cm^{-2}$ measured with M101 - Power Meter Interface with USB, RS232, UART, and Analog Operation, Thorlabs GmbH): 470 nm, 385 lx and 0.095 $mW \cdot cm^{-2}$; white light, 400 lx and 0.018 $mW \cdot cm^{-2}$).

Illumination	Wavelength (nm)	Light Intensity (lx)	Light Intensity ($mW \cdot cm^{-2}$)	Reference
White Light		400	0.018	Ledflexi Inspire, Leroy Merlin, Spain
Blue Light	470	385	0.095	RS Components, 769-3549, Broadcom

Table S5. Illumination conditions in light/dark transition test.

The animals were introduced into the darkest compartment (the one with the minimum light intensity for each study), while the opening between the two compartments remained blocked, thus preventing the escape reflex of the animals. After 3 s, the passage between the two compartments was cleared and the animal was allowed to explore freely for 3 min, with the time spent in each compartment being timed. The procedure was repeated three times (allowing animals to rest for approximately one hour between repetitions) for each subject to ensure reproducibility. The different compartments were thoroughly cleaned, removing all faeces, and sprayed with a disinfectant agent to eliminate any odour that the animals could detect. In addition, after cleaning, the compartments were inverted, preventing the animals from recognising the spaces.

During the experimental time, the room was kept in total darkness and any noise or external influence from the operator was kept to a minimum to create the least stressful environment possible and to allow the animal to feel free to explore. All experiments were carried out during the nocturnal phase of the animals (highest activity), avoiding interference with circadian cycles.

Experimental Procedure

GA animals (*Opn4*$^{-/-}$) were subjected to the Light/Dark Transition Test prior to intraperitoneal administration of $NaIO_3$. When it was found that the animals did not show any visual alteration, $NaIO_3$

was administered systemically by intraperitoneal injection of a dose of 65 mg/kg, with a 25 G needle. The animals were returned to their respective cages and allowed to rest for 28 days approximately. In the case of the *Pde6b*$^{rd10/rd10}$ × *Opn4*$^{-/-}$, they were subjected to the test at 90 days postnatal, when the neurodegeneration was almost completed. In this case it was not possible to perform the test prior the neurodegeneration because animals were too young.

Prior to administration of the photoswitches, a new Light/Dark Transition Test was performed, checking that the animals did not show rejection of the compartments with higher light intensity. Subsequently, the animals were anaesthetised with isoflurane and the various photosensitive molecules were administered individually, by unilateral intravitreal injection, with a 10 µL syringe (NanofilTm, World Precision Instruments, Florida, USA) with a 34 G needle (NanofilTm, NF35BV-2, World Precision Instruments, Florida, USA), or by topical application of a 10 µL drop on the ocular surface of both eyes. The animals were allowed to rest for 2 h and 30 min and then re-introduced to the Light/Dark Transition Test.

To study the effect of photosensitive molecules in the presence of the antagonist 1-Bz-APDC, the animals (*Pde6b*$^{rd10/rd10}$ × *Opn4*$^{-/-}$) were left to rest for 7 days. After this time, the Light/Dark Transition Test was carried out again to ensure the loss of effectiveness of the photosensitive molecules. The animals were again anaesthetised and a monocular dose of 10 µM prosthe6-12 was applied topically. The test was performed in the same manner and then 1 µL of 1-Bz-APDC antagonist was injected into the same eye. The animals were allowed to recover for a few min and the Transition Test was performed again.

All animals were placed in the darkest compartment in all cases. The test was repeated three times for each animal, each time cleaning each compartment to avoid falsification of the results. All experimental procedures were carried out following very similar schedules, during the night phase of the animals, to ensure a higher activity of the animals, as well as a higher reproducibility. One *Pde6b*$^{rd10/rd10}$ x *Opn4*$^{-/-}$ male displayed an abnormal fearful behaviour in the Light/Dark Transition test (Figure S25, panel c) and was removed from the statistical analysis.

We studied the influence of sex on transition test results in the groups where the distribution was homogeneous. The results (Figure S25) show that there are no significant differences between females and males, using mixed-effects ANOVA analysis (Sidak's multiple comparisons). Note that Figure 5 combines both sexes.

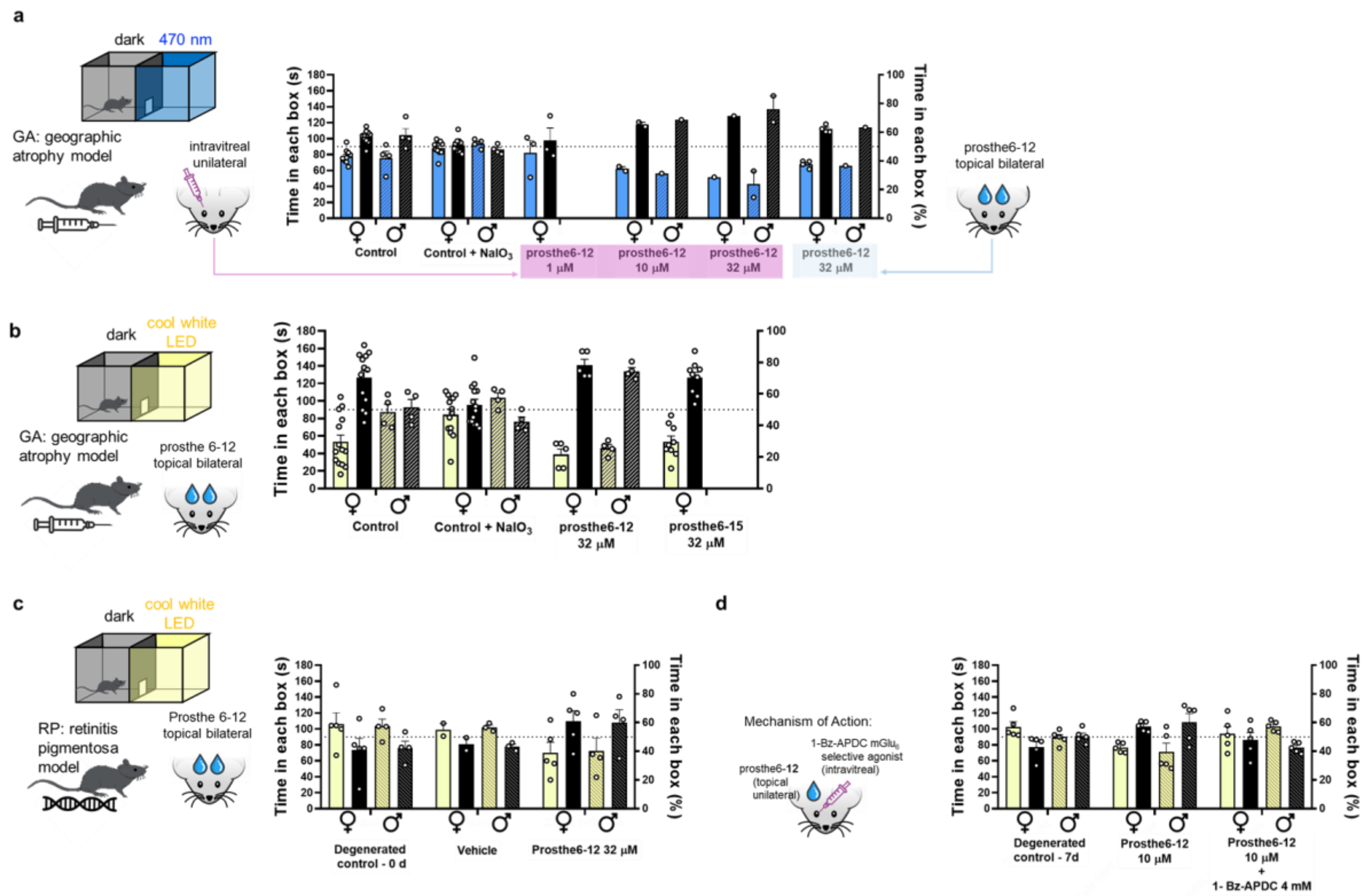

Figure S25. Prosthe6 restores light-driven behaviour in murine models of geographic atrophy (GA) and retinitis pigmentosa (RP), regardless of the animal's sex. (a) The administration of prosthe6-12 in the GA mouse model displayed a significant increase of preference for the dark room in all (intravitreal and topical) cases under blue light without being affected by the sex of the animals. **(b)** Similar results were observed under white light illumination in the GA model, and **(c-d)** in the RP model. Statistical analysis showed, in all cases, that there were no significant differences between sex in the time the animals spent in each compartment, indicating that this factor did not introduce experimental bias into the data. Statistical differences across all panels are determined by comparing the time recorded for each sex in the dark box, using mixed-effects ANOVA analysis (Sidak's multiple comparisons). Data are presented as mean ± SEM of the average times recorded for the dark and light boxes, to facilitate comparison of the results (see Figure 5 for sex combined data). One male outlier not considered in panel c (n= 5 females, 4 males, see Experimental Procedure).

9. Pharmacokinetic profile of prosthe6-12

Pharmacokinetic characterization of prosthe6-12 and development of the corresponding quantification method (LC-MS/MS MRM) were conducted by Creatio, Production and Validation Center of Advanced Therapies, University of Barcelona. Male New Zealand White Rabbits (10-12 weeks old) were administered prosthe6-12 either intravitreally (100 µL of 150 µM solution with 2 % DMSO) or topically (100 µL of 32 µM solution 2 % DMSO). Male rabbits were used in this early PK study due to their larger eye volume. Based on this validation, future studies will include both sexes. Plasma, vitreous humor and retina samples were obtained at 0.5h, 1, 3, 6 and 12 hours post intravitreal injection and at 1, 3, 6 and 12 hours post topical instillation (n=3 per timepoint and route of administration). Mild adverse clinical signs were observed in three rabbits following intravitreal injection, attributed to the administration procedure. Prosthe6-12 plasma concentrations were below the limit of quantification (LOQ, 0.104 ng·mL^{-1}) at all timepoints for both administration routes. LOQ in vitreous and retina samples was 0.33 ng·ml^{-1}. For representation purposes, datapoints below the LOQ were input as LOQ/2 in Figure S26.[10]

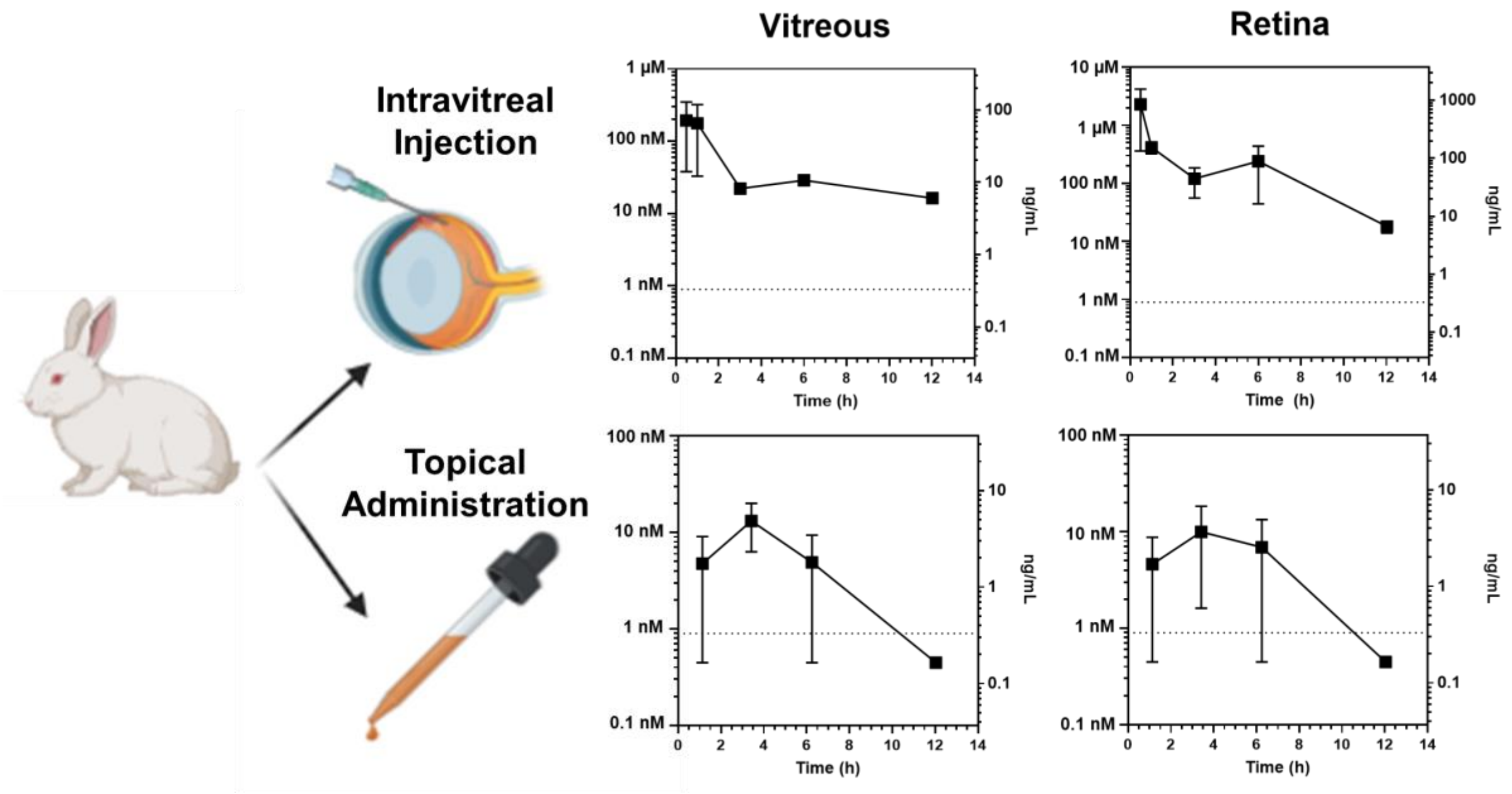

Figure S26. Prosthe6-12 pharmacokinetic profile. Quantification of prosthe6-12 in vitreous and retina after intravitreal or topical administration of the compound in New Zealand White Rabbits. Intravitreal injection (top row) was performed at 0.5, 1, 3, 6, and 12 hours post-dosing (N = 3 per time point), while topical administration (bottom row) was evaluated at 1, 3, 6, and 12 hours (N = 3 per time point). Dotted line: limit of quantification (LOQ). Data points below LOQ were input as LOQ/2 for representation purposes. Data are shown as mean ± SEM.

Route of administration	Sample	Time post administration (hh:mm)				
		00:30	01:00	03:00	06:00	12:00
Intravitreal injection (ng·mL^{-1})	Vitreous humor	186	9.7	7.61	8.36	6.42
		16.3	171	9.85	9.85	6.42
		11.8	14.6	7.01	13.7	5.22
	Retina	2264	85.9	90.8	13.6	4.96
		152	202	30.2	19.1	5.31
		115	174	12.1	233	9.71
	Plasma	<0.104	<0.104	<0.104	<0.104	<0.104
		<0.104	<0.104	<0.104	<0.104	<0.104
		<0.104	<0.104	<0.104	<0.104	<0.104
Topical instillation (ng·mL^{-1})	Vitreous humor	N/A	4.93	8.81	5.07	< 0.33
		N/A	< 0.33	5.52	< 0.33	< 0.33
		N/A	< 0.33	< 0.33	< 0.33	< 0.33
	Retina	N/A	4.75	9.8	7.27	< 0.33
		N/A	< 0.33	1.04	< 0.33	< 0.33
		N/A	< 0.33	< 0.33	< 0.33	< 0.33
	Plasma	N/A	<0.104	<0.104	<0.104	<0.104
		N/A	<0.104	<0.104	<0.104	<0.104
		N/A	<0.104	<0.104	<0.104	<0.104

Table S6: Prosthe6-12 pharmacokinetic profile. Prosthe6-**12** concentration (ng·mL^{-1}) after intravitreal and topical administration in vitreous humor, retina and plasma samples. Each datapoint corresponds to an individual White New Zealand rabbit. Limit of quantification in retina and vitreous was 0.33 ng·mL^{-1}, and 0.104 ng·mL^{-1} in plasma. 0.5 hours post administration sampling was only performed for intravitreal injections. N/A: not applicable.

10. Supplementary References

(1) Monn, J. A.; Valli, M. J.; Johnson, B. G.; Salhoff, C. R.; Wright, R. A.; Howe, T.; Bond, A.; Lodge, D.; Spangle, L. A.; Paschal, J. W.; et al. Synthesis of the Four Isomers of 4-Aminopyrrolidine-2,4-dicarboxylate: Identification of a Potent, Highly Selective, and Systemically-Active Agonist for Metabotropic Glutamate Receptors Negatively Coupled to Adenylate Cyclase. *Journal of Medicinal Chemistry* **1996**, *39* (15), 2990-3000. DOI: 10.1021/jm9601765. Valli, M. J.; Schoepp, D. D.; Wright, R. A.; Johnson, B. G.; Kingston, A. E.; Tomlinson, R.; Monn, J. A. Synthesis and metabotropic glutamate receptor antagonist activity of N1-substituted analogs of 2R,4R-4-aminopyrrolidine-2,4-dicarboxylic acid. *Bioorganic & Medicinal Chemistry Letters* **1998**, *8* (15), 1985-1990.
(2) Hampson, D. R.; Huang, X. P.; Pekhletski, R.; Peltekova, V.; Hornby, G.; Thomsen, C.; Thogersen, H. Probing the ligand-binding domain of the mGluR4 subtype of metabotropic glutamate receptor. *J Biol Chem* **1999**, *274* (47), 33488-33495. DOI: 10.1074/jbc.274.47.33488 From NLM Medline.
(3) Gomeza, J.; Mary, S.; Brabet, I.; Parmentier, M. L.; Restituito, S.; Bockaert, J.; Pin, J. P. Coupling of metabotropic glutamate receptors 2 and 4 to G alpha 15, G alpha 16, and chimeric G alpha q/i proteins: characterization of new antagonists. *Mol Pharmacol* **1996**, *50* (4), 923-930. From NLM Medline.
(4) Tox-Prediction. *ProTox 3.0 - Prediction Of Toxicity Of Chemicals*. 2024.
(5) Tochitsky, I.; Trautman, J.; Gallerani, N.; Malis, J. G.; Kramer, R. H. Restoring visual function to the blind retina with a potent, safe and long-lasting photoswitch. *Sci Rep* **2017**, *7*, 45487. DOI: 10.1038/srep45487 From NLM Medline.
(6) Lipinski, C. A.; Lombardo, F.; Dominy, B. W.; Feeney, P. J. Experimental and computational approaches to estimate solubility and permeability in drug discovery and development settings. *Adv Drug Deliv Rev* **2001**, *46* (1-3), 3-26. DOI: 10.1016/s0169-409x(00)00129-0 From NLM Medline.
(7) Hattar, S.; Liao, H. W.; Takao, M.; Berson, D. M.; Yau, K. W. Melanopsin-containing retinal ganglion cells: architecture, projections, and intrinsic photosensitivity. *Science* **2002**, *295* (5557), 1065-1070. DOI: 10.1126/science.1069609 From NLM.
(8) Veleri, S.; Lazar, C. H.; Chang, B.; Sieving, P. A.; Banin, E.; Swaroop, A. Biology and therapy of inherited retinal degenerative disease: insights from mouse models. *Dis Model Mech* **2015**, *8* (2), 109-129. DOI: 10.1242/dmm.017913 From NLM. Gargini, C.; Terzibasi, E.; Mazzoni, F.; Strettoi, E. Retinal organization in the retinal degeneration 10 (rd10) mutant mouse: a morphological and ERG study. *J Comp Neurol* **2007**, *500* (2), 222-238. DOI: 10.1002/cne.21144 From NLM. Barhoum, R.; Martínez-Navarrete, G.; Corrochano, S.; Germain, F.; Fernandez-Sanchez, L.; de la Rosa, E. J.; de la Villa, P.; Cuenca, N. Functional and structural modifications during retinal degeneration in the rd10 mouse. *Neuroscience* **2008**, *155* (3), 698-713. DOI: 10.1016/j.neuroscience.2008.06.042 From NLM. Chang, B.; Hawes, N. L.; Pardue, M. T.; German, A. M.; Hurd, R. E.; Davisson, M. T.; Nusinowitz, S.; Rengarajan, K.; Boyd, A. P.; Sidney, S. S.; et al. Two mouse retinal degenerations caused by missense mutations in the β-subunit of rod cGMP phosphodiesterase gene. *Vision Research* **2007**, *47* (5), 624-633. Pittler, S. J.; Baehr, W. Identification of a nonsense mutation in the rod photoreceptor cGMP phosphodiesterase beta-subunit gene of the rd mouse. *Proceedings of the National Academy of Sciences* **1991**, *88* (19), 8322-8326. DOI: doi:10.1073/pnas.88.19.8322.
(9) Milla-Navarro, S.; Pazo-Gonzalez, M.; Germain, F.; de la Villa, P. Phenotype Characterization of a Mice Genetic Model of Absolute Blindness. *Int J Mol Sci* **2022**, *23* (15). DOI: 10.3390/ijms23158152 From NLM Medline.

(10) Wijk, M.; Wasmann, R. E.; Jacobson, K. R.; Svensson, E. M.; Denti, P. A Pragmatic Approach to Handling Censored Data Below the Lower Limit of Quantification in Pharmacokinetic Modeling. *CPT Pharmacometrics Syst Pharmacol* **2025**, *14* (6), 1042-1049. DOI: 10.1002/psp4.70015 From NLM.
